# PLASMODESMATA-LOCATED PROTEIN 6 regulates plasmodesmal function in Arabidopsis vasculature

**DOI:** 10.1093/plcell/koae166

**Published:** 2024-06-06

**Authors:** Zhongpeng Li, Su-Ling Liu, Christian Montes-Serey, Justin W Walley, Kyaw Aung

**Affiliations:** Department of Genetics, Development, and Cell Biology, Iowa State University, Ames, IA 50011, USA; Department of Genetics, Development, and Cell Biology, Iowa State University, Ames, IA 50011, USA; Department of Plant Pathology, Entomology and Microbiology, Iowa State University, Ames, IA 50011, USA; Department of Plant Pathology, Entomology and Microbiology, Iowa State University, Ames, IA 50011, USA; Plant Sciences Institutes, Iowa State University, Ames, IA 50011, USA; Department of Genetics, Development, and Cell Biology, Iowa State University, Ames, IA 50011, USA

## Abstract

Plasmodesmata connect adjoining plant cells, allowing molecules to move between the connected cells for communication and sharing resources. It has been well established that the plant polysaccharide callose is deposited at plasmodesmata, regulating their aperture and function. Among proteins involved in maintaining callose homeostasis, PLASMODESMATA-LOCATED PROTEINSs (PDLPs) promote callose deposition at plasmodesmata. This study explored the function of PDLP5 and PDLP6 in different cell types. We discovered that PDLP5 and PDLP6 are expressed in nonoverlapping cell types in Arabidopsis (*Arabidopsis thaliana*). The overexpression of *PDLP5* and *PDLP6* results in the overaccumulation of plasmodesmal callose at different cell interfaces, indicating that PDLP5 and PDLP6 are active in different cell types. We also observed 2 distinct patterns of starch accumulation in mature leaves of *PDLP5* and *PDLP6* overexpressors. An enzyme-catalyzed proximity labeling approach was used to identify putative functional partners of the PDLPs. We identified SUCROSE SYNTHASE 6 (SUS6) as a functional partner of PDLP6 in the vasculature. We further demonstrated that PDLP6 physically and genetically interacts with SUS6. In addition, CALLOSE SYNTHASE 7 (CALS7) physically interacts with SUS6 and PDLP6. Genetic interaction studies showed that CALS7 is required for PDLP6 function. We propose that PDLP6 functions with SUS6 and CALS7 in the vasculature to regulate plasmodesmal function.

## Teaser

PLASMODESMATA-LOCATED PROTEIN 6 functions with SUCROSE SYNTHASE 6 and CALLOSE SYNTHASE 7 in the vasculature to regulate plasmodesmal function.

## Introduction

Different cell types have evolved specialized functions to support the coordinated functioning of multicellular organisms. While maintaining their distinct cellular identities, cells must communicate effectively with surrounding cells and tissues. Cell-to-cell communication occurs through membrane-lined channels found in all 3 domains of life. In plants, plasmodesmata serve as conduits that connect the plasma membrane (PM), endoplasmic reticulum (ER), and cytoplasm of adjacent cells, facilitating the exchange of signals and resources among cells (Roberts and Oparka 2003; Lucas et al. 2009; Maule et al. 2012; Brunkard et al. 2015; Tilsner et al. 2016; Sager and Lee 2018). The plasmodesmata-dependent communication among cells is fundamental for developmental regulation and stress responses in plants (Lee and Lu 2011; Jackson 2015; Sevilem et al. 2015; Cheval and Faulkner 2018; Reagan and Burch-Smith 2020; Miras et al. 2022). In addition to allowing intercellular movement of signaling molecules, plasmodesmata serve as conduits for the movement of metabolites, including sugar, between adjoining cells (Braun 2022).

It is well documented that the plant polysaccharide callose (β-1,3-glucan) is deposited at plasmodesmata within cell walls to restrict their aperture (De Storme and Geelen 2014; Wu et al. 2018; German et al. 2023), regulating plasmodesmal function. CALLOSE SYNTHASEs (CALSs) catalyze callose biosynthesis. It has been proposed that CALSs form multisubunit enzyme complexes with SUCROSE SYNTHASEs (SUSs; Amor et al. 1995), which generate uridine diphosphate glucose (UDPG), and other proteins. CALSs utilize UDPG as a glucose donor to synthesize callose (Li et al. 2003; Brownfield et al. 2007). In addition, recent findings highlighted the roles of PLASMODESMATA-LOCATED PROTEINSs (PDLPs) in modulating plasmodesmal function. The Arabidopsis (*Arabidopsis thaliana*) genome encodes 8 PDLPs, sequentially named PDLP1 to PDLP8 (Thomas et al. 2008). PDLPs are type I membrane proteins containing a signal peptide, 2 domains of unknown function 26 (DUF26), and a transmembrane domain followed by a short stretch of cytoplasmic tail (Thomas et al. 2008). The extracellular domain of PDLPs exhibits high structural similarity to fungal lectins, which bind mannose; however, the plant polysaccharide binding ability of PDLPs is unclear (Vaattovaara et al. 2019). Recent research reported that PDLP5 forms protein complexes with NON-RACE SPECIFIC DISEASE RESISTANCE/HIN1 HAIRPIN-INDUCED-LIKE PROTEIN 3 (NHL3) to transmit multiple immune signals to activate CALS1 and promote plasmodesmal callose deposition in Arabidopsis (Tee et al. 2023).

Misexpression of different *PDLP* members has been demonstrated to impact plant growth, development, and defense. The expression of *PDLP5* exhibits a significant correlation with callose accumulation at plasmodesmata in both Arabidopsis (Lee et al. 2011) and *Nicotiana benthamiana* (Li et al. 2021). The overexpression of *PDLP5* inhibits plasmodesmal function, while the *pdlp5* knockout mutant has enhanced movement of molecules through plasmodesmata (Lee et al. 2011; Li et al. 2021). Arabidopsis transgenic plants overexpressing *PDLP1* (Thomas et al. 2008) and *PDLP5* (Lee et al. 2011) have delayed plant growth. Furthermore, the spatiotemporal expression of *PDLP5* is governed by auxin, specifically influencing the regulation of lateral root emergence in Arabidopsis (Sager et al. 2020). During *Hyaloperonospora arabidopsidis* infection, PDLP1 is targeted to haustoria-associated membranes and involved in callose deposition (Caillaud et al. 2014). In response to bacterial infection, the expression of *PDLP5* transcripts is upregulated, and PDLP5 protein accumulation increases (Lee et al. 2008, 2011). In addition, the overexpression of *PDLP5* leads to an overaccumulation of salicylic acid, a major defense hormone, alongside various growth defects in Arabidopsis (Lee et al. 2011). Moreover, PDLP1 and PDLP5 are integral for systemic acquired resistance (Lim et al. 2016) and reactive oxygen species waves in systemic tissues during biotic and abiotic stress (Fichman et al. 2021). While Arabidopsis transgenic plants overexpressing *PDLP5* show compromised calcium waves induced by wounding (Toyota et al. 2018), the temporary closure of plasmodesmata resulting from inducible callose accumulation in *LexA::icals3m* transgenic plants fails to impede wound-induced calcium waves in Arabidopsis (Bellandi et al. 2022). These findings underscore the diverse roles of PDLPs in plants, simultaneously raising further questions to answer.

Here, we characterized the functions of PDLPs by overexpressing them in Arabidopsis. The overexpression of *PDLP5* and *PDLP6* resulted in starch hyperaccumulation in mature leaves and delayed plant growth. We further demonstrated that PDLP5 and PDLP6 accumulate and function at different cell interfaces. In addition, we identified functional partners of PDLP6 in regulating plasmodesmal callose accumulation in the vasculature. Our findings suggest that PDLP6 functions with SUS6 and CALS7 in the phloem, likely in sieve elements (SE), to regulate plasmodesmal function.

## Results

### The overexpression of *PDLP6* affects plant growth and starch accumulation

**Figure 1.**
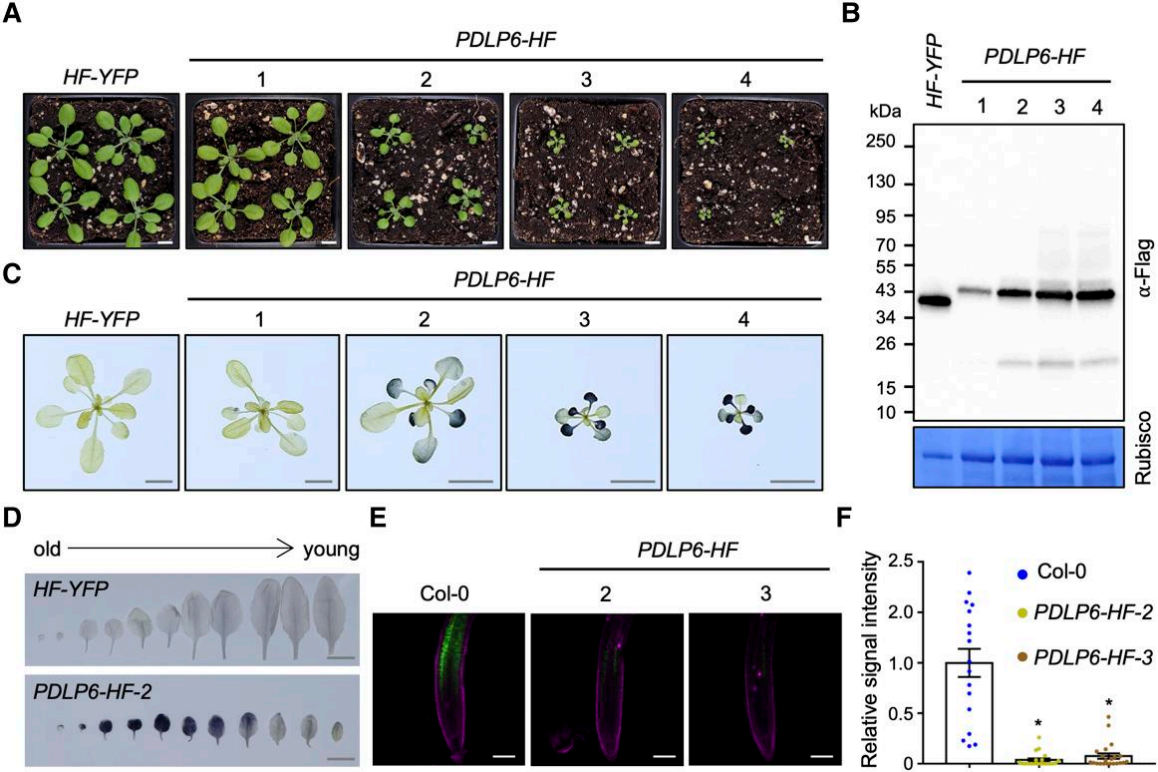
The overexpression of PDLP6 leads to growth defects, starch overaccumulation, and reduced long-distance phloem transportation. **A)** Three-week-old Arabidopsis plants were grown under a light intensity used for a standard Arabidopsis growth (110 *µ*mol/m^2^/s). Images were taken using the same magnification. Scale bar = 1 cm. Four independent transgenic lines (1 to 4) were shown. *HF-YFP* and *PDLP6-HF* refer to *Pro35S:HF-YFP* and *Pro35S:PDLP6-HF*, respectively. **B)** Immunoblot analysis detects the expression of PDLP6-HF in 4 independent transgenic plants. An anti-Flag antibody was used to detect the expression of Flag fusion proteins. Rubisco serves as a loading control. Numbers on the side indicate molecular weights in kilodaltons (kDa). **C)** Starch accumulation of plants shown in **A)**. Tissues were harvested at the end of the night and stained using Lugol's iodine solution. Images were taken using the same magnification. Scale bars = 1 cm. **D)** Starch staining of leaves from Col-0 and *PDLP6-HF*. Leaves are arranged according to their age. Scale bars = 1 cm. **E)** A CFDA loading assay determined long-distance phloem transport. CF signals were detected in root tips. PI was used as a root cell wall stain. Scale bars = 100 *µ*m. **F)** CF fluorescent signal intensity in roots was quantified using Fiji. Each dot represents the relative signal intensity of CF from an individual root. The plot shows the mean with SEM. Col-0, *n* = 17; *PDLP6-HF-2*, *n* = 20; and *PDLP6-HF-3*, *n* = 22. Asterisks indicate statistically significant differences analyzed with a 2-tailed *t-*test (*P < 0.01).

To determine the function of PDLPs, we individually overexpressed all 8 *PDLP* genes (*PDLP1* to *PDLP8*) in Arabidopsis wild-type Col-0 using a 35S promoter (*Pro35S*). The PDLPs were fused with His and Flag (HF) tags. We also generated Arabidopsis transgenic plants overexpressing free yellow fluorescent protein (YFP) as a control (*Pro35S:HF-YFP*, hereafter *HF-YFP*). From our first round of screening, we isolated Arabidopsis transgenic plants overexpressing *PDLP5* and *PDLP6* (*Pro35S:PDLP5-HF* and *Pro35S:PDLP6-HF*, hereafter *PDLP5-HF* and *PDLP6-HF*) with a delayed growth phenotype (Fig. 1A; Supplementary Fig. S1A). Immunoblot analyses showed that the transgenic plants with more severe delayed growth accumulated a much higher level of PDLP6-HF compared with the transgenic lines with more normal plant growth (Fig. 1, A and B). A similar trend was also observed for *PDLP5-HF* (Supplementary Fig. S1, A and B; Lee et al. 2011). In addition, *PDLP6-HF* accumulated more anthocyanin in mature leaves and exhibited late-flowering phenotypes (Supplementary Fig. S1, C to G). The observed growth phenotypes are similar to Arabidopsis mutants *suc2* and *sweet11;12*, which are compromised in sugar transport from mature leaves to sink tissues, including young leaves, roots, flowers, and seeds.

In addition to the growth phenotype, these mutants overaccumulated starch in mature leaves (Gottwald et al. 2000; Srivastava et al. 2008; Chen et al. 2012; Wippel and Sauer 2012). We hypothesize that the overexpression of the *PDLP*s inhibits plasmodesmata-dependent movement of sugar in mature leaves, leading to starch hyperaccumulation. We thus determined starch content in the transgenic plants using a Lugol's iodine staining method (Tran et al. 2019). Tissues were harvested at the end of the night for starch staining when overall starch accumulation in mature leaves was at its lowest (Graf et al. 2010). Under a light intensity used for standard Arabidopsis growth (110 *µ*mol/m^2^/s), we found that *PDLP6-HF* hyperaccumulated starch in old and mature rosette leaves, exhibiting a dark blue color (Fig. 1, C and D). Like the plant growth phenotype, transgenic plants with a higher expression level of *PDLP6* exhibited darker color in their mature leaves (Fig. 1, B and C).

We next determined the effect of *PDLP6* overexpression on shoot-to-root long-distance phloem trafficking using 5(6)-carboxyfluorescein diacetate (CFDA) feeding assay (Oparka et al. 1994; Knoblauch et al. 2015; Huang et al. 2019). CFDA was applied to a half-clipped cotyledon of 7-d-old Arabidopsis seedlings. Despite delayed plant growth (Fig. 1A), no severe growth defects were observed for *PDLP6-HF* seedlings compared with wild-type Col-0 at this developmental stage (Supplementary Fig. S1H). Plant cells can rapidly take up CFDA and cleave it into carboxyfluorescein (CF), a polar fluorescent compound, using intracellular esterases. Subsequently, CF moves from leaf to root via phloem. The presence of CF in the root tips of CFDA-fed seedlings was detected and quantified. Compared with wild-type Col-0, we observed a significant decrease in CF signals in the root tips of *PDLP6-HF* lines (Fig. 1, E and F). The findings suggest that the overexpression of *PDLP6-HF* hinders long-distance phloem transport.

To further characterize the function of PDLP6, we generated *pdlp6-1* and *pdlp6-2* mutants using CRISPR/Cas9 technology (Tsutsui and Higashiyama 2017). We isolated *pdlp6-1* and *pdlp6-2* mutants carrying 26 bp deletion and a G to T mutation around a guide RNA target site, respectively (Supplementary Fig. S2A). The deletion and mutation led to a frameshift and premature termination of PDLP6 translation. The development and growth of *pdlp6* mutants were indistinguishable from that of Col-0 (Supplementary Fig. S2B). Similarly, no differences in starch content were observed between Col-0 and *pdlp6* mutants using a Lugol's solution staining method (Supplementary Fig. S2, C and D). However, we observed fewer starch grains in chloroplasts within mature leaves of *pdlp5* and *pdlp6-1* mutant compared with the wild-type Col-0 using transmission electron microscopy (TEM; Supplementary Fig. S2E). The minor differences observed between *pdlp6-1* and Col-0 could be attributed to the functional redundancy of PDLPs (Supplementary Fig. S2, F and G).

### The overexpression of *PDLP5* or *PDLP6* leads to distinct starch hyperaccumulation patterns

**Figure 2.**
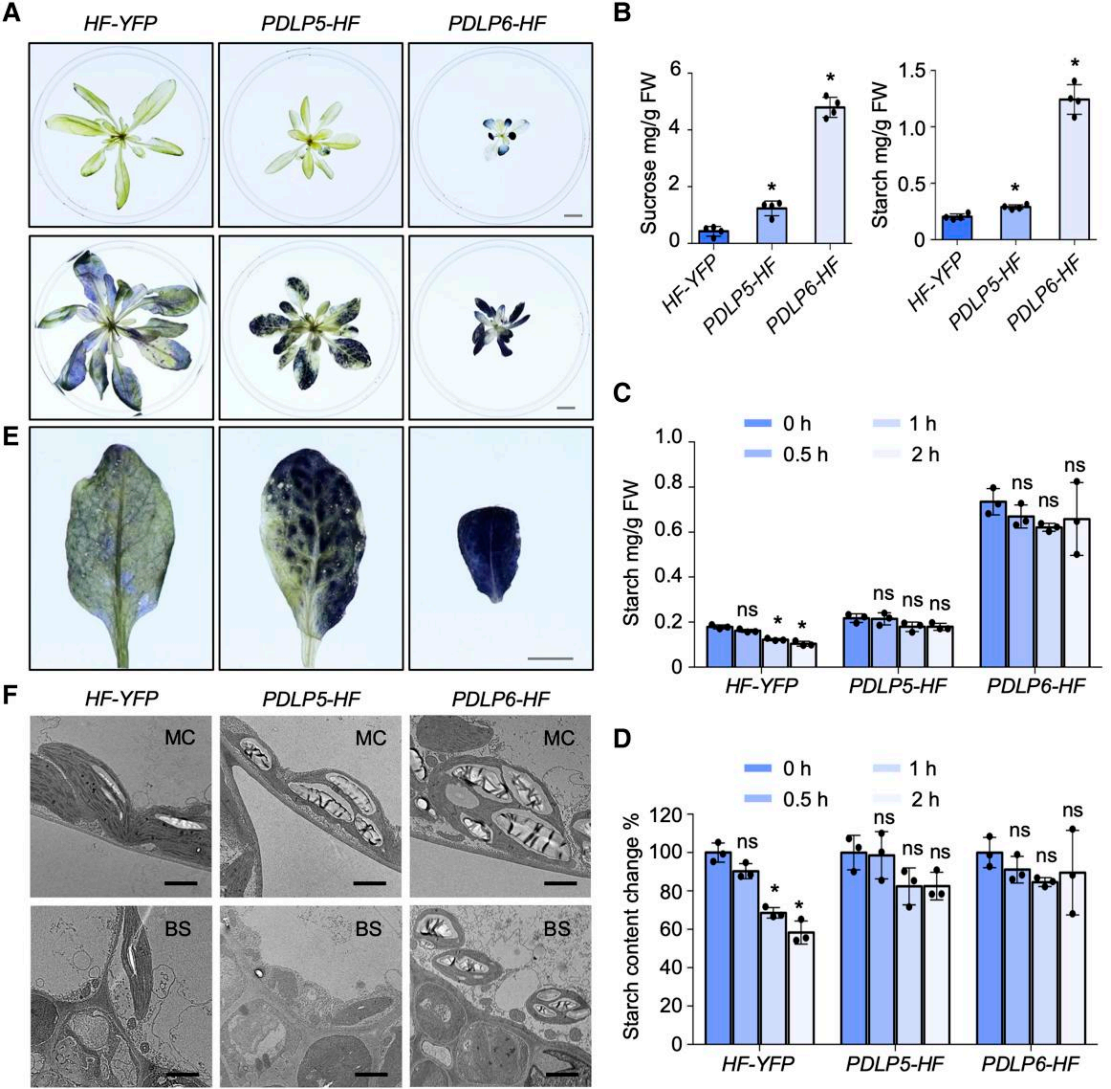
The overexpression of PDLP5 and PDLP6 leads to different starch overaccumulation patterns. **A)** Starch staining of Arabidopsis plants. Top panel: 4-wk-old plants grown under a standard light intensity used for Arabidopsis growth condition (110 *µ*mol/m^2^/s); lower panel: 5-wk-old plants that were irradiated with high light (200 *µ*mol/m^2^/s) for 10 d before staining. Samples were collected at the end of the night for starch staining using Lugol's iodine solution. Images were taken using the same magnification. Scale bar = 1 cm. **B)** Quantification of sucrose and starch contents in leaves of high light-treated plants at the end of the night. Mature leaves from 3 plants were combined to form a single replicate. The plots show the mean with Sd (*n* = 4). Asterisks indicate statistically significant differences analyzed with a 2-tailed *t-*test (*P < 0.01). **C)** Quantification of starch content in leaves of high light-treated plants at various time points. The “0 h” designation represents the end of the day; 0.5, 1, and 2 h indicate time points following the end of the day. Mature leaves from 3 plants were combined to form a single replicate. The plots show the mean with Sd (*n* = 3). The values at 0.5, 1, and 2 h time points were compared with that at 0 h for each genotype. Asterisks denote statistically significant differences analyzed with a 2-tailed *t-*test (*P < 0.01). ns, no significance. **D)** Changes in starch content relative to the end of the day at different time points. The plots show the mean with Sd (*n* = 3). The values at 0.5, 1, and 2 h time points were compared with that at 0 h for each genotype. Asterisks denote statistically significant differences analyzed with a 2-tailed *t-*test (*P < 0.01). ns, no significance. **E)** Starch accumulation patterns in source leaves of different genotypes. Images were captured with the same magnification. Scale bar = 0.5 cm. **F)** TEM images show the starch granules in chloroplasts in mesophyll and bundle sheath cells of mature leaves. MC, mesophyll cell; BS, bundle sheath cell. Scale bars = 2 *µ*m.

Under a light intensity used for a standard Arabidopsis growth (110 *µ*mol/m^2^/s), we observed starch hyperaccumulation in *PDLP6-HF* (*#2*), but not in *PDLP5-HF* (*#2*) (Fig. 2A, upper panel). Similar to *sweet11;12* (Chen et al. 2012), the starch hyperaccumulation phenotype became apparent in *PDLP5-HF* after the plants were irradiated with high light intensity (200 *µ*mol/m^2^/s) for 10 d before the starch staining (Fig. 2A, lower panel). We also quantified the sucrose and starch content in mature leaves using a biochemical approach (Leach and Braun 2016). Compared with *HF-YFP*, a higher level of sucrose and starch was detected in *PDLP5-HF* and *PDLP6-HF* at the end of the night (Fig. 2B). We determined the changes in starch content at various time points after the end of the day. During this period, starch is degraded and sucrose is synthesized, which is then transported out from the mature leaves. Col-0 exhibited an approximately 40% decrease in starch content within 2 h of darkness, while *PDLP5-HF* and *PDLP6-HF* did not show a significant reduction in starch content during the dark period (Fig. 2, C and D). Our findings suggest that the overexpression of PDLP5 and PDLP6 compromises sugar transportation in mature leaves.

In addition, we observed distinct starch accumulation patterns in the mature leaves of *PDLP5-HF* and *PDLP6-HF*. *PDLP6-HF* accumulated starch evenly, whereas *PDLP5-HF* lacked starch accumulation in and around vascular tissues in mature leaves (Fig. 2E). To determine starch accumulation in different cell types, leaf discs from high light-treated plants were subjected to histological sectioning and starch staining. Periodic acid/Schiff (PAS) reagent labels polysaccharides in the cell wall and starch grains in chloroplasts. Consistent with the whole tissue staining (Fig. 2E), *PDLP5-HF* and *PDLP6-HF* exhibited darker PAS-stained starch grains in chloroplasts within mesophyll cells compared with *HF-YFP* (Supplementary Fig. S3A). In line with our prediction, *PDLP5-HF* contained less starch in bundle sheath cells, whereas *PDLP6-HF* contained many PAS-stained starch grains in their bundle sheath cells (Supplementary Fig. S3A). We also performed a TEM analysis to visualize starch grains in different cell types within mature leaves. *PDLP5-HF* and *PDLP6-HF* contained larger starch grains in chloroplasts within mesophyll cells compared with their control, whereas enlarged starch grains were only observed in bundle sheath cells of *PDLP6-HF* (Fig. 2F; Supplementary Fig. S3B). Together, our findings suggest that the overexpression of *PDLP5* results in starch hyperaccumulation in mesophyll cells within mature leaves, while the overexpression of *PDLP6* leads to starch hyperaccumulation in both mesophyll and bundle sheath cells.

### *PDLP5* and *PDLP6* are expressed in different cell types

**Figure 3.**
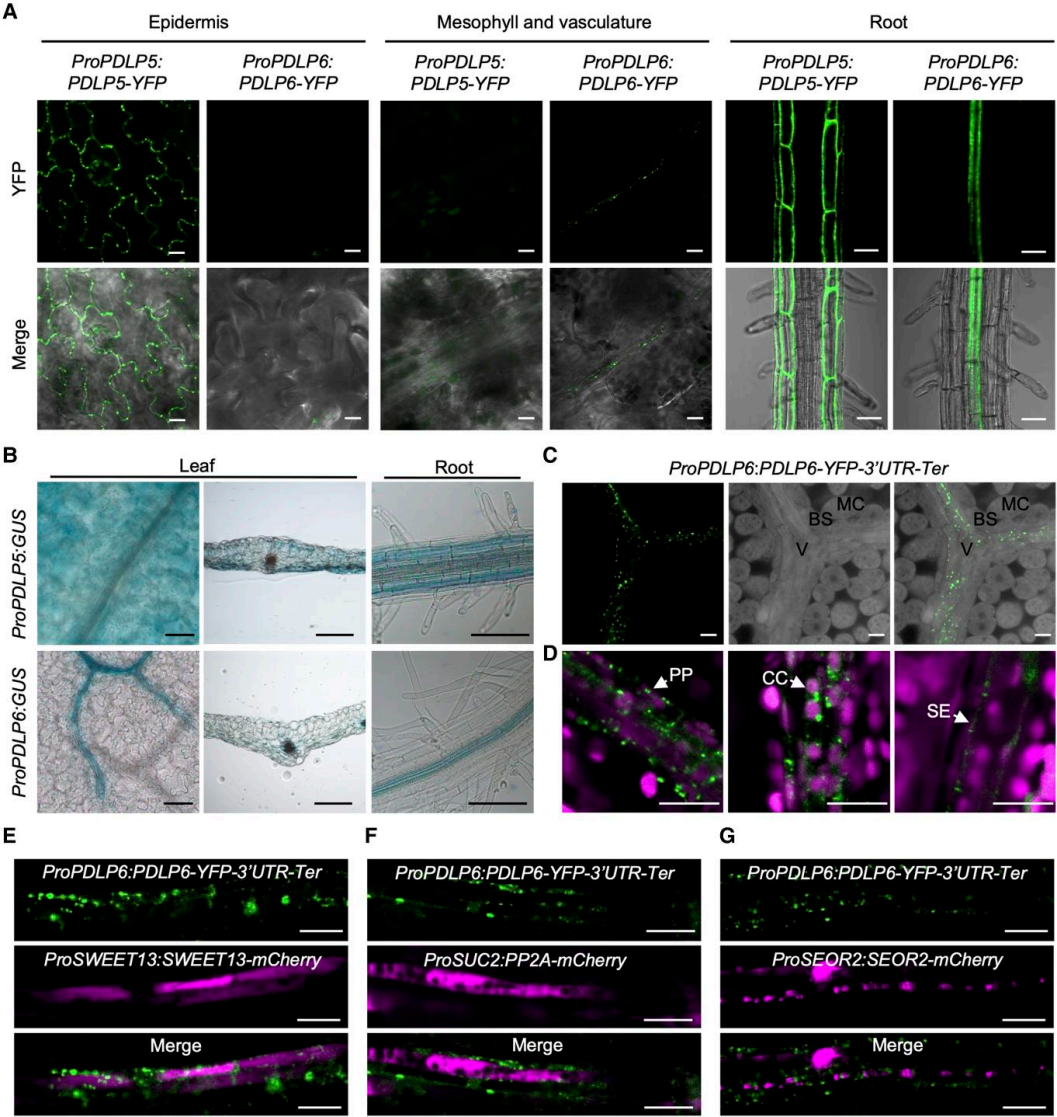
PDLP5 and PDLP6 are expressed in different cell types. **A)** The cell type–specific expression of PDLP5-YFP and PDLP6-YFP. The fusion proteins were driven by their own native promoter. Confocal images were captured from 2-wk-old Arabidopsis seedlings. The signal of the YFP fusion proteins was detected in different cell types (top panel). Merged images show the signals from YFP and bright-field (lower panel). Scale bars for epidermis and vasculature = 10 *µ*m. Scale bars for root = 50 *µ*m. **B)** Histochemical staining of GUS activity in Arabidopsis *ProPDLP5*:*GUS* and *ProPDLP6*:*GUS* transgenic plants. Images were captured from the leaf surface, leaf transverse sections, and roots. Scale bars = 100 *µ*m. **C)** PDLP6-YFP proteins were observed in the vasculature. The leaf sample was cleared by ClearSee solution. MC, mesophyll cell; BS, bundle sheath cell; V, vasculature. Scale bars = 10 *µ*m. **D)** PDLP6-YFP proteins were detected in PP cells, CC, and SE. Cell types were determined based on their sizes, chloroplast arrangement, and cell wall ingrowth phenotype as previously described (Cayla et al. 2015). Chlorophyll autofluorescence was also shown. Scale bar = 10 *µ*m. **E to G)** The colocalization of PDLP-YFP signals with various cell type markers. **E)***ProSWEET13:SWEET13-mCherry* marks PP cells. **F)***ProSUC2:PP2A1-mCherry* marks CC. **G)***ProSEOR2:SEOR2-mCherry* makes SE. Scale bars = 10 *µ*m.

Single-cell RNA sequencing analysis revealed that *PDLP* transcripts showed distinct expression patterns in different cells in the Arabidopsis leaf. Especially, *PDLP6* was detected in phloem parenchyma (PP) cells (Kim et al. 2021). To determine the cell type–specific expression of PDLP5 and PDLP6, we constructed *ProPDLP5:PDLP5-YFP* and *ProPDLP6:PDLP6-YFP*. We amplified the native promoter and ORF (including 5′ UTR and introns) of *PDLP5* and *PDLP6* to tag YFP at their C-terminus. We used confocal microscopy to detect the expression of YFP fusion proteins in Arabidopsis transgenic plants carrying *ProPDLP5:PDLP5-YFP* or *ProPDLP6:PDLP6-YFP*. The expression of PDLP5-YFP was mainly detected between epidermal cells, whereas no PDLP5-YFP signals were observed in leaf vasculature (Fig. 3A). PDLP6-YFP, on the other hand, was detected predominantly in leaf vasculature. Similarly, PDLP6-YFP was expressed predominantly in root vasculature, whereas PDLP5-YFP was detected in the epidermis and cortex (Fig. 3A). We also used native promoters and 5′ UTR of *PDLP5* or *PDLP6* (*ProPDLP5* or *ProPDLP6*) to drive the expression of GUS. Histochemical staining showed that the PDLP5 promoter was active in the leaf epidermis and mesophyll and the root epidermis and cortex. PDLP6 promoter, on the other hand, was active specifically in leaf and root phloem (Fig. 3B).

We noted that the expression of PDLP6-YFP was difficult to detect in leaves of *ProPDLP6:PDLP6-YFP* using confocal microscopy. Since the noncoding region of *SWEET11* plays a crucial role in PP-specific expression of the SWEET11 protein (Zhang et al. 2021), we generated a new construct to include 3′ UTR and terminator of *PDLP6* (hereafter *ProPDLP6:PDLP6-YFP-3′UTR-Ter*). In the transgenic lines, we detected the expression of PDLP6-YFP in the vasculature (Fig. 3C), specifically in PP cells, companion cells (CC), and SE in leaves (Fig. 3D). We determined the cell types based on their size, chloroplast arrangement, and cell wall ingrowth phenotype as previously described (Cayla et al. 2015). Furthermore, we observed the colocalization of PDLP6-YFP signals with various cell-type markers (Fig. 3, E to G). PP cells, CC, and SE were marked using *ProSWEET13:SWEET13-mCherry* (Kim et al. 2021), *ProSUC2:PP2A1-mCherry* (Cayla et al. 2015), and *ProSEOR2:SEOR2-mCherry* (Cayla et al. 2015), respectively.

### The overexpression of PDLP5 and PDLP6 affects plasmodesmal function at different cell interfaces

**Figure 4.**
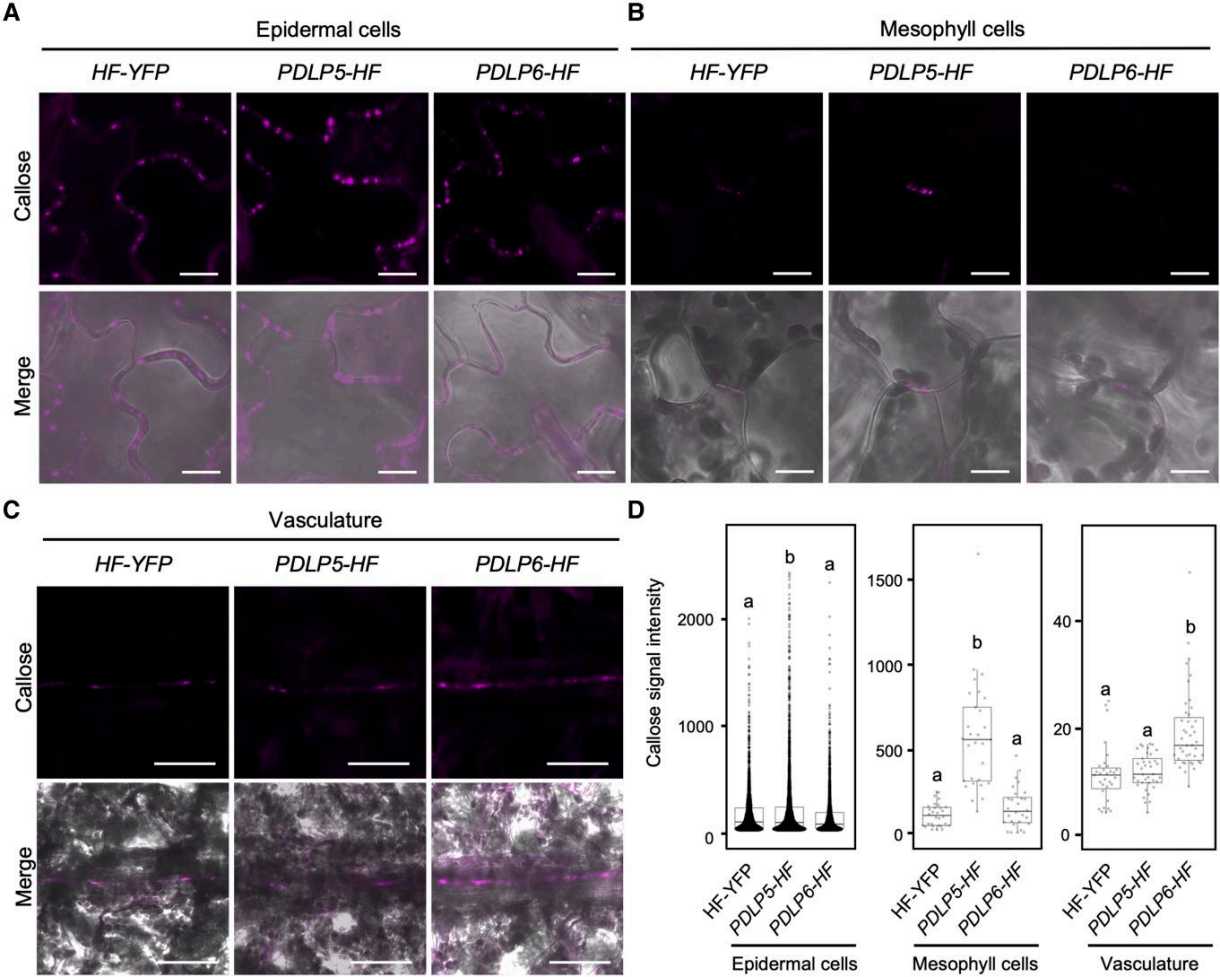
PDLP5 and PDLP6 regulate PD callose accumulation at different cell interfaces. **A)** Callose accumulation between leaf epidermal. Puncta signals represent aniline blue–stained callose at plasmodesmata (top panel). Merged images show the signals from callose and bright-field (lower panel). Scale bars = 10 *µ*m. **B)** Callose accumulation between mesophyll cells. **C)** Callose accumulation in the leaf vasculature. Scale bars = 50 *µ*m. **D)** Quantitative data show callose accumulation between epidermal cells, mesophyll cells, and the vasculature. Each dot represents aniline blue–stained callose in the epidermal and mesophyll cells. Mean signal intensity in the vasculature was determined by measuring the vasculature area and total signal intensity. The box plots show the mean with Sd. *HF-YFP*, *n* = 2,937 (50 images); *PDLP5-HF*, *n* = 4,205 (49 images); and *PDLP6-HF*, *n* = 2,499 (41 images) for epidermis. *HF-YFP*, *n* = 30 (25 images); *PDLP5-HF*, *n* = 29 (20 images); and *PDLP6-HF*, *n* = 28 (21 images) for mesophyll cell. *HF-YFP*, *n* = 31 (31 images); *PDLP5-HF*, *n* = 37 (37 images); and *PDLP6-HF*, *n* = 43 (43 images) for the vasculature. Images were captured from 3 leaves, each from a different plant. Different letters on the bar indicate statistically significant differences analyzed with 1-way ANOVA (*P* < 0.0001).

The overexpression of *PDLP5* induced callose overaccumulation at plasmodesmata between epidermal cells in Arabidopsis and *N. benthamiana* (Lee et al. 2011; Li et al. 2021). Given the distinct cell type expression of PDLP5 and PDLP6 as well as the distinct starch hyperaccumulation patterns in *PDLP5* and *PDLP6* overexpression lines, we examined the effect of PDLP overexpression on the accumulation of plasmodesmal callose at different cell interfaces. The transgenic plants were subjected to aniline blue staining to detect plasmodesmal callose. Consistent with the previous report (Lee et al. 2011), we observed a higher callose accumulation between epidermal cells in *PDLP5-HF* leaves compared with *HF-YFP* (Fig. 4, A, B, and D). We also detected a higher callose level at plasmodesmata connecting mesophyll cells in *PDLP5-HF* leaves (Fig. 4, A, B, and D). The overexpression of PDLP6, on the other hand, did not affect plasmodesmal callose accumulation between epidermal and mesophyll cells in leaves (Fig. 4, A, B, and D). As native *PDLP6* is specifically expressed in the vasculature, we examined the callose accumulation in the leaf vasculature. *PDLP6-HF* exhibited higher callose accumulation in vascular tissues, likely SE, compared with *HF-YFP* and *PDLP5-HF*, while there were no significant differences between *HF-YFP* and *PDLP5-HF* (Fig. 4, C and D). These findings support that the overexpression of PDLP5 and PDLP6 leads to a higher plasmodesmal callose accumulation at specific cell interfaces.

**Figure 5.**
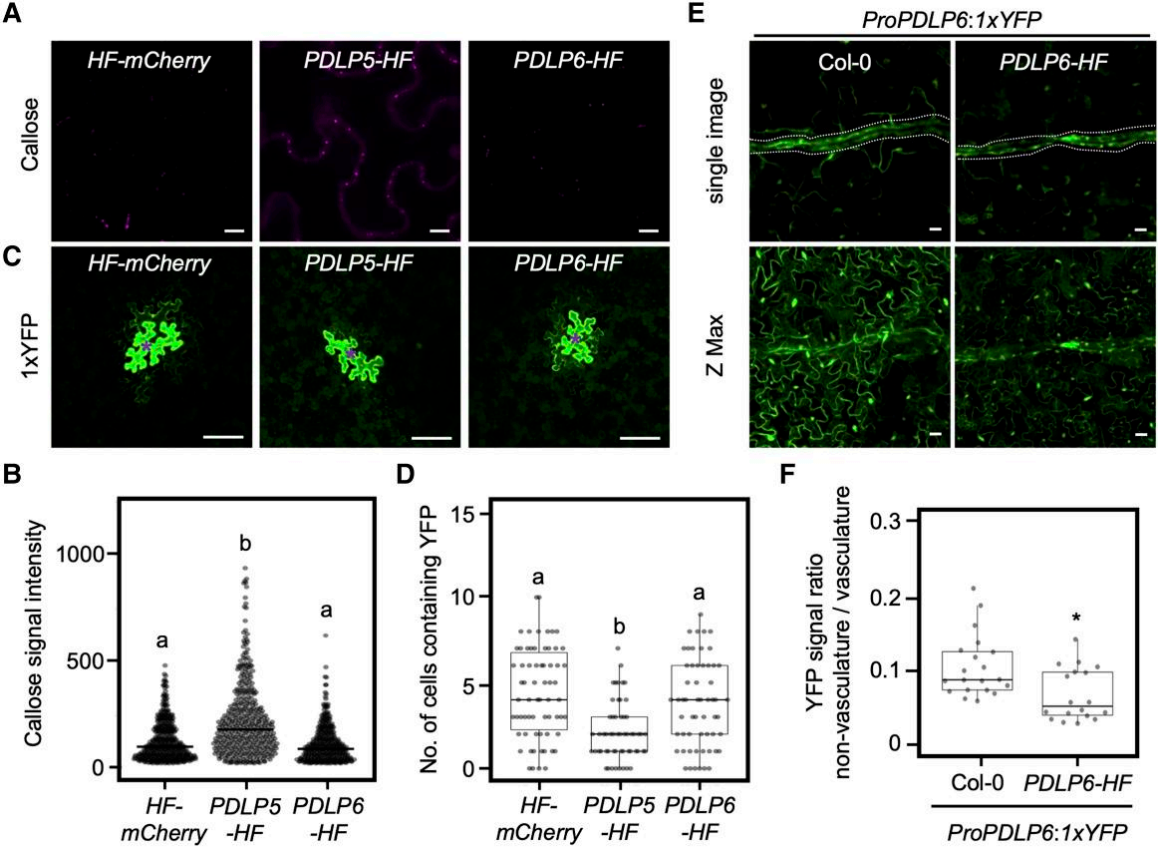
PDLP5 and PDLP6 regulate plasmodesmal function at different cell interfaces. **A)** Plasmodesmal callose accumulation in *N. benthamiana* leaves transiently overexpressing HF-mCherry, PDLP5-HF, or PDLP6-HF. Confocal images show aniline blue–stained callose between epidermal cells. Scale bars = 20 *µ*m. **B)** Quantitative data present plasmodesmal callose accumulation. Each dot represents an aniline blue–stained callose. The box plots show the mean with Sd. *HF-mCherry*, *n* = 425; *PDLP5-HF*, *n* = 428; and *PDLP6-HF*, *n* = 362. The number of images used for quantification is as follows: *HF-mCherry*, 13; *PDLP5-HF*, 12; and *PDLP6-HF*, 14. Images were captured from 3 leaves. Different letters on the bar indicate statistically significant differences analyzed with 1-way ANOVA (*P* < 0.0001). **C)** Plasmodesmata-dependent movement of YFP in *N. benthamiana* leaves transiently overexpressing HF-mCherry, PDLP5-HF, or PDLP6-HF. Confocal images show the diffusion of 1xYFP from the transformed cells (indicated by asterisks) to the surrounding pavement cells. Scale bars = 100 *µ*m. **D)** Quantitative data present plasmodesmata-dependent movement of 1xYFP. Each dot represents an individual transformed cell. The box plots show the mean with Sd. *HF-mCherry*, *n* = 66; *PDLP5-HF*, *n* = 56; and *PDLP6-HF*, *n* = 57. Images were captured from 4 to 5 leaves. Different letters on the bar indicate statistically significant differences analyzed with 1-way ANOVA (*P* < 0.0001). **E)** The diffusion of YFP from the vasculature to mesophyll and pavement cells in leaves of Col-0 and *PDLP6-HF* expressing *ProPDLP6*:*1xYFP*. Signals were detected in cotyledons of 14-d-old seedlings. Scale bar = 20 *µ*m. **F)** Quantification of the YFP signals diffused from the vasculature to the other cell types. Mean signal intensity was determined in the vasculature (dotted area) and nonvasculature near the leaf tip. The ratio between nonvasculature and vasculature was calculated. A cotyledon from each seedling was used for imaging. The box plots show the mean with Sd. Col-0, *n* = 20; *PDLP6-HF*, *n* = 18. The asterisk indicates statistically significant differences analyzed with a Mann–Whitney *U* test (*P < 0.05).

To further investigate the functions of PDLP5 and PDLP6 in distinct cell types, we transiently overexpressed them in *N. benthamiana.* Consistent with our previous report (Li et al. 2021), the transient overexpression of PDLP5 resulted in a higher callose deposition at plasmodesmata between epidermal cells and inhibits the plasmodesmata-dependent YFP diffusion (Fig. 5, A to D). On the other hand, the overexpression of PDLP6 did not affect plasmodesmal function in epidermal cells (Fig. 5, A to D). To determine the function of PDLP6 in regulating the plasmodesmata-dependent movement of molecules in the vasculature, we generated Arabidopsis transgenic plants carrying *ProPDLP6:1xYFP* construct in wild-type Col-0 and *PDLP6-HF* backgrounds to express 1xYFP specifically in the phloem. A similar strategy using *ProSUC2:1xGFP* was applied to determine the PD-dependent movement of fluorescent proteins from phloem to surrounding cells (Imlau et al. 1999). Figure 5, E and F showed that YFP levels in nonvascular cells were significantly higher in Col-0 compared with *PDLP6-HF*. These results suggest that the overexpression of PDLP6 inhibits the plasmodesmata-dependent movement of molecules from the vasculature to the surrounding cells. These findings also suggest that plasmodesmal function is inhibited in epidermal and mesophyll cells in *PDLP5-HF* plants, while in *PDLP6-HF* plants, it is inhibited in the vasculature. This result corresponds with the distinct patterns of starch hyperaccumulation observed in these transgenic plants.

Our findings raise an intriguing question of how the ubiquitous expression of the PDLP proteins gives rise to the function of the proteins in distinct cell types. Using a constitutive promoter *Pro35S*, PDLP5-HF and PDLP6-HF are expected to express in most cell types. The assumption was supported by the findings that PDLP5-YFP and PDLP6-YFP were detected in most cell types in leaves and roots of Arabidopsis transgenic plants, *Pro35S:PDLP5-YFP* and *Pro35S:PDLP6-YFP* (Supplementary Fig. S4). The distinct starch accumulation patterns (Fig. 2) and the differential regulation of plasmodesmal function in various cell types (Fig. 5) in *PDLP5-HF* and *PDLP6-HF* transgenic lines suggest that the ubiquitous expression of PDLPs does not uniformly impact plasmodesmal function in all cell types. The findings also show that the impact of overexpressing PDLP5 and PDLP6 is most pronounced in the cell types where they are naturally expressed. The ubiquitous expression of PDLP5 does not lead to the inhibition of plasmodesmal function in the vasculature. Similarly, the ubiquitous expression of PDLP6 does not affect plasmodesmal function in epidermal and mesophyll cells. As PDLPs are not predicted to catalyze callose biosynthesis, the enzymes or proteins that function together with the PDLPs in synthesizing callose might also be expressed in the same cell types.

### Identification of putative PDLP5 and PDLP6 functional partners using an enzyme-catalyzed proximity labeling approach

**Figure 6.**
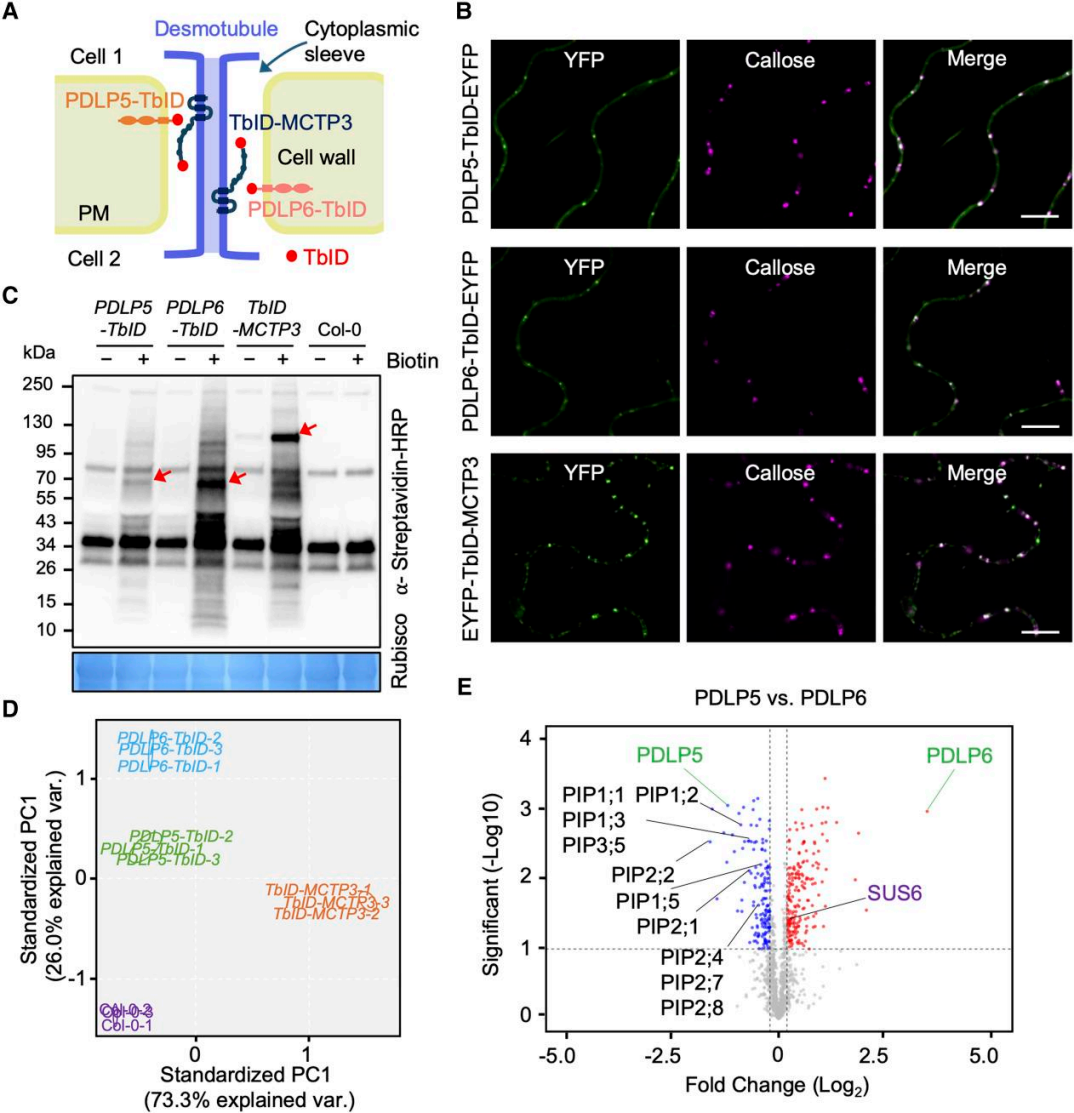
A PL assay identifies functional partners of plasmodesmal proteins. **A)** A diagram shows the localization of PDLP5-TbID, PDLP6-TbID, and TbID-MCTP3 fusion proteins to different membranes of plasmodesmata. **B)** Plasmodesmal localization of PDLP5-TbID-EYFP, PDLP6-TbID-EYFP, and EYFP-TbID-MCTP3. Agrobacteria harboring *ProUBQ10:PDLP5-TurboID-EYFP-3xFlag* (PDLP5-TbID-EYFP), *ProUBQ10:PDLP6-TurboID-EYFP-3xFlag* (PDLP6-TbID-EYFP), and *ProUBQ10:3xFlag-EYFP-TurboID-MCTP3* (EYFP-TbID-MCTP3) were infiltrated into *N. benthamiana* to transiently overexpress the EYFP fusion proteins. The plasmodesmal localization of the EYFP fusion proteins was imaged using confocal microscopy. YFP indicates the signal of the EYFP fusion proteins. Callose indicates the aniline blue–stained callose signal. Merged images show the plasmodesmal localization of the EYFP fusion proteins. Scale bars = 10 *µ*m. **C)** Two-week-old seedlings of wild-type Col-0 and Arabidopsis transgenic plants, *ProUBQ10:PDLP5-TurboID-3xFlag* (*PDLP5-TbID*), *ProUBQ10:PDLP6-TbID-3xFlag* (*PDLP6-TbID*), and *ProUBQ10:3xFlag-TbID-MCTP3* (*TbID-MCTP3*), were subjected to biotin treatment and immunoblot analysis using a Streptavidin-HRP antibody to determine the activity of the TbID fusion proteins. Rubisco serves as a loading control. Arrows indicate the biotinylated TbID fusion proteins. Numbers on the side indicate molecular weights in kDa. **D)** PCA was performed and visualized using the ggbiplot R package as part of the TMT-NEAT analysis pipeline. Three replicates (1 to 3) were analyzed for each genotype. **E)** Volcano plots show significantly enriched proteins in PDLP5 and PDLP6 samples. Candidates were filtered using cutoffs log_2_FC > 0.2 or <−0.2 and *q* < 0.1. Plots were generated using VolcaNoseR. PIPs and SUS6 proteins are labeled.

To identify functional partners of PDLP5 and PDLP6, we conducted an enzyme-catalyzed proximity labeling (PL) assay. Recent studies have successfully used the method to identify functional partners of plant proteins, including nuclear and Golgi membrane proteins (Mair et al. 2019; Zhang et al. 2019; Huang et al. 2020; Zhou et al. 2023). We also included MULTIPLE C2 DOMAINS AND TRANSMEMBRANE REGION PROTEIN 3 (MCTP3), as it is targeted to the ER membrane of PD (PD-ER; also known as desmotubule; Brault et al. 2019), in contrast to PDLP5 and PDLP6, which are localized to the PM within PD (PD-PM; Fig. 6A; Thomas et al. 2008; Lee et al. 2011). To maximize our chance of identifying the functional partners of the bait proteins, we chose to express the proteins with a constitutive promoter *UBQ10*. We generated the following transgenic lines: *ProUBQ10:PDLP5-TurboID-3×Flag*, *ProUBQ10:PDLP6-TurboID-3×Flag*, and *ProUBQ10:3×Flag-TurboID-MCTP3* (hereafter *PDLP5-TbID*, *PDLP6-TbID*, and *TbID-MCTP3*; Fig. 6A). We observed that the transgenic plants expressing PDLP6-TbID exhibited delayed plant growth (Supplementary Fig. S5, A and B). In parallel, we confirmed the expression and plasmodesmal localization of PDLP5-TbID-EYFP, PDLP6-TbID-EYFP, and TbID-MCTP3-EYFP in *N. benthamiana* (Fig. 6B; Supplementary Fig. S5C).

We conducted the PL assay on *PDLP5-TbID*, *PDLP6-TbID*, *TbID-MCTP3*, and wild-type Col-0. We included Col-0 to detect the baseline of biotinylated proteins in Arabidopsis (Supplementary Fig. S5). The expression and enzymatic activity of the TbID fusion proteins in the transgenic plants were detected using immunoblot analysis (Fig. 6C; Supplementary Fig. S5B). Total biotinylated proteins were enriched and subjected to LC with tandem mass spectrometry for protein identification. Principal component analysis (PCA) showed a clear separation of the samples by genotype (Fig. 6D). The bait proteins were specifically enriched in the transgenic plants expressing the corresponding fusion proteins (Supplementary Fig. S5, D to I and Data Set 1). Many known plasmodesmata-localized proteins were enriched by PDLP5-TbID, PDLP6-TbID, and/or TbID-MCTP3 (Supplementary Fig. S5, D to I). Several proteins with potential functions at PD-ER (e.g. MCTP4, RTNLB6, and HVA22C; Knox et al. 2015; Brault et al. 2019) were significantly enriched by TbID-MCTP3 (Supplementary Fig. S5, D to H). PDLP5-TbID and PDLP6-TbID, on the other hand, enriched many confirmed plasmodesmal proteins targeted to PD-PM (e.g. CLV1, REM1.2, and REM1.3; Stahl et al. 2013; Huang et al. 2019; Supplementary Fig. S5, D to H). Our findings suggest that the PL assay is a powerful tool for identifying functional partners of plasmodesmal proteins. Given the juxtaposition of the 2 membrane systems, the PM and the ER membrane, the PL assay shows promise in resolving the functional protein complexes at a nanometer resolution within plasmodesmata.

### PDLP6 functions together with SUS6

**Figure 7.**
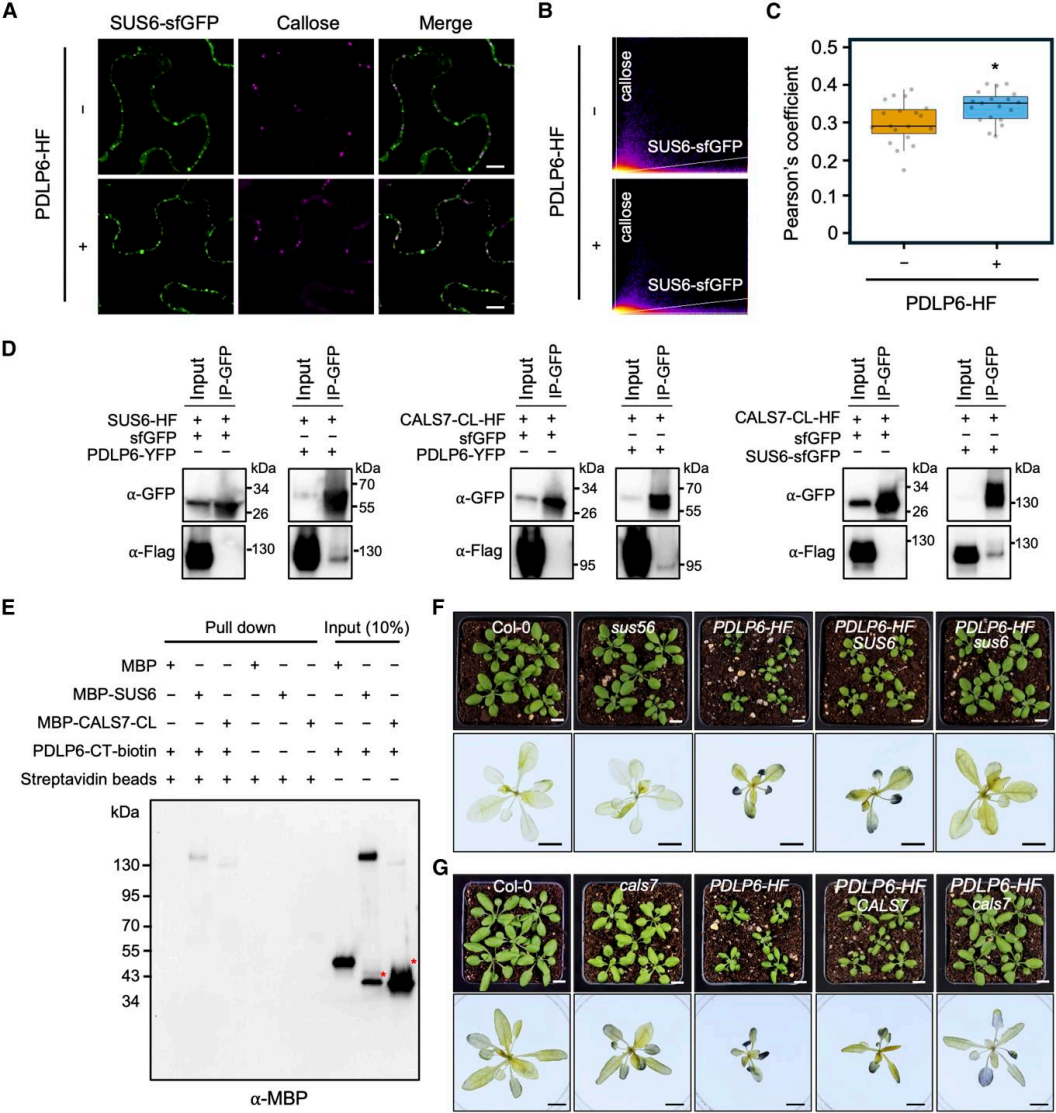
PDLP6 functions together with SUS6 and CALS7. **A)** Confocal images show plasmodesmal localization of SUS6-sfGFP with or without overexpressing PDLP6-HF. The fusion proteins were transiently overexpressed in *N. benthamiana*. Aniline blue–stained callose marked plasmodesmata. Scale bars = 10 *µ*m. **B** and **C)** Pearson's coefficient analysis shows that the expression of PDLP6 increases the plasmodesmal localization of SUS6-sfGFP. Each dot represents Pearson's coefficient value calculated for a merged image. Two sets of 19 images were analyzed to quantify the plasmodesmal localization of SUS6-sfGFP, comparing samples with and without overexpression of PDLP6-HF. Images were captured from 3 leaves. The box plots show the mean with Sd (*n* = 19). An asterisk indicates statistically significant differences analyzed with a Mann–Whitney *U* test (*P < 0.05). **D)** Co-IP assay indicates the interaction between PDLP6-SUS6, PDLP6-CALS7, and SUS6-CALS7. Agrobacteria harboring different plasmids were co-infiltrated into *N. benthamiana*. Samples were collected 2 d after infiltration and subjected to co-IP. A GFP antibody was used to detect the expression of sfGFP fusion protein and the enrichment of sfGFP fusion proteins by GFP-Trap. A Flag antibody was used to detect the expression of HF fusion protein and the interaction between HF and sfGFP fusion proteins. A free sfGFP fusion protein was included as a negative control. Numbers on the side indicate molecular weights in kDa. **E)** In vitro pull-down assay shows the direct interaction between PDLP6, SUS6, and CALS7. The biotinylated C-terminal tail of PDLP6 (PDLP6-CT-biotin) was incubated with recombinant proteins, MBP, MBP-SUS6, or MBP-CALS7. Magnetic beads coupled with streptavidin were used to pull down PDLP6-CT-biotin and the interacting proteins. A MBP antibody was used to detect the interaction between PDLP6 and MBP fusion proteins. Asterisks indicated nonspecific bands for MBP-SUS6 and MBP-CALS7-CL. Numbers on the side indicate molecular weights in kDa. **F)** Genetic interactions between *PDLP6* and *SUS6*. **G)** Genetic interactions between *PDLP6* and *CALS7*. Homozygous lines were isolated from F_3_ progenies of the genetic crosses of *sus56* with *PDLP6-HF-2* and *cals7* with *PDLP6-HF-2*. Images in the lower panel show the starch accumulation phenotype. Plants were collected at the end of the night for starch staining using Lugol's iodine solution. Images were taken using the same magnification. Scale bar = 1 cm.

PDLP5-TbID and PDLP6-TbID enriched similar proteins as their putative functional partners (Supplementary Fig. S5, D to H and Data Set 1). Despite the similarity, PDLP5 and PDLP6 significantly enriched subsets of proteins. PDLP5 enriched several members of PLASMA MEMBRANE INTRINSIC PROTEINs (PIPs; also known as AQUAPORINs), while PDLP6 significantly enriched SUCROSE SYNTHASE 6 (SUS6; Fig. 6E). It is noted that PDLP5-TbID and TbID-MCTP3 also significantly enriched SUS6 when compared with the Col-0 control; however, PDLP6 showed the highest level of enrichment (Supplementary Fig. S5, D to I). Furthermore, PDLP6-TbID showed a significant enrichment of SUS6 compared with both PDLP5-TbID and TbID-MCTP3, whereas PDLP5-TbID did not exhibit a significant enrichment of SUS6 compared with TbID-MCTP3 (see Supplementary Fig. S5, D and E). Given the potential roles of SUS6 in callose biosynthesis in Arabidopsis SE (Barratt et al. 2009), we further characterized the relationship between PDLP6 and SUS6. The Arabidopsis genome encodes 6 members of SUSs, sequentially named SUS1 to SUS6 (Baud et al. 2004). Among them, SUS6 was expressed specifically in SE (Yao et al. 2020). The findings suggest that PDLP6 might function with SUS6 to regulate callose biosynthesis in vascular tissues, likely in SE. We first determined the plasmodesmal association of SUS6 by transiently expressing SUS6-superfolder GFP (sfGFP) in *N. benthamiana*. We observed that a portion of SUS6-sfGFP signals colocalized with aniline blue–stained callose (Fig. 7A), suggesting that SUS6 can localize to plasmodesmata. Moreover, the transient overexpression of PDLP6-HF increased plasmodesmal association of SUS6-sfGFP (Fig. 7, A to C; Supplementary Fig. S6A).

We next confirmed the presence of PDLP6 and SUS6 within the same protein complex using a co-immunoprecipitation (co-IP) assay. PDLP6-YFP and SUS6-HF were transiently expressed in *N. benthamiana*. As shown in Fig. 7D, PDLP6-YFP enriched SUS6-HF, while a free sfGFP did not. The findings suggest that SUS6 is present in PDLP6-containing protein complexes. To further determine the direct physical interaction between PDLP6 and SUS6, we conducted an in vitro pull-down assay. Figure 7E showed that a biotinylated C-terminal tail of PDLP6 (PDLP6-CT-biotin), which faces the cytoplasmic sleeve, specifically pulled down the maltose binding protein fused SUS6 (MBP-SUS6). We also detected the physical interaction between PDLP5 and SUS6 using the same co-IP and in vitro pull-down assay (Supplementary Fig. S7, A and C). PDLP7 shares higher amino acid similarity with PDLP5 and PDLP6 than other PDLP members (Thomas et al. 2008). In addition, *PDLP7* transcripts were detected in PP cells (Kim et al. 2021). We thus tested the physical interaction between PDLP7 and SUS6 using the co-IP and in vitro pull-down assay. We did not detect the presence of PDLP7 and SUS6 in the same protein complex using a co-IP assay (Supplementary Fig. S7D). As PDLP7-CT-biotin bound to a free MBP, the findings from the assay are inconclusive (Supplementary Fig. S7F). The findings confirmed the physical interaction between SUS6 and PDLP6 (also PDLP5).

To elucidate the relationship between SUS6 and PDLP6, we introduced *sus6* mutation into *PDLP6-HF* via a genetic cross. We first generated a cross between the *sus56* double mutant (Bieniawska et al. 2007; Baroja-Fernández et al. 2012) and *PDLP6-HF*. F_2_ segregating progenies were screened for *PDLP6* overexpressor in *SUS6* wild-type background and *sus6* mutant background (hereafter *PDLP6-HF SUS6* and *PDLP6-HF sus6*; Fig. 7F; Supplementary Fig. S6B). We performed a PCR genotyping assay to identify the genotypes of SUS5 and SUS6 (Supplementary Fig. S6C). The expression level of PDLP6-HF was determined using immunoblot analysis. The selected lines expressed a comparable level of PDLP6-HF (Supplementary Fig. S6D). *PDLP6-HF SUS6* exhibited a delayed plant growth phenotype like *PDLP6-HF*, while *sus6* largely suppressed the plant growth phenotype of *PDLP6-HF* (Fig. 7F; Supplementary Fig. S6, B and E). In addition, *sus6* also suppressed starch hyperaccumulation in mature leaves of *PDLP6-HF* (Fig. 7F). The findings suggest that SUS6 is required for PDLP6's function.

### PDLP6 functions together with CALS7

Callose biosynthesis is catalyzed by CALSs (De Storme and Geelen 2014; Wu et al. 2018; German et al. 2023). CALS1 has been demonstrated to play an important role in regulating callose biosynthesis and plasmodesmal function in Arabidopsis (Cui and Lee 2016; Tee et al. 2023). Our PL assay showed that TbID-MCTP3, PDLP5-TbID, and PDLP6-TbID significantly enriched a CALS protein group, including CALS1, CALS2, and CALS4, compared with wild-type Col-0. However, PDLP5-TbID and PDLP6-TbID did not significantly enrich the CALS protein group compared with TbID-MCTP3 (Supplementary Fig. S5I and Data Set 1). To examine the physical interaction among CALS1, PDLP5, and PDLP6, we performed an in vitro pull-down assay. As the cytoplasmic loop (CL) of CALSs was predicted to interact with SUS6 to form callose synthase complex (Verma and Hong 2001; De Storme and Geelen 2014), we cloned and purified the CL of MBP-CALS1 (MBP-CalS1-CL). Supplementary Fig. S7G showed that PDLP5-CT-biotin and PDLP6-CT-biotin did not pull down MBP-CALS1-CL. The findings suggest that CALS1 might not be a strong interacting protein of PDLP5 and PDLP6, at least not with the CALS1-CL.

As *CALS7* was previously reported to be expressed specifically in SE (Barratt et al. 2011; Xie et al. 2011; Kalmbach et al. 2023), we hypothesized that PDLP6 functions together with CALS7 and SUS6 in the phloem, likely in SE. Although we did not identify CALS7 from our PL assay, we examined the interactions among PDLP6, SUS6, and CALS7 to test the hypothesis. To this end, we cloned *pro35S:CALS7-CL-HF* and *MBP-CALS7-CL* for a co-IP assay and an in vitro pull-down assay, respectively. Using a co-IP assay, we detected the presence of PDLP6 and CALS7-CL within the same protein complex (Fig. 7D). We also detected CALS7-CL and SUS6 in the same protein complex (Fig. 7D). Using an in vitro pull-down assay, we observed the direct physical interaction between PDLP6-CT-biotin and MBP-CALS7-CL (Fig. 7E). We also detected the physical interaction between PDLP5 and CALS7-CL using the same co-IP and in vitro pull-down assay (Supplementary Fig. S7, B and C). However, CALS7-CL was not detected in the PDLP7-containing protein complexes (Supplementary Fig. S7, E and F). The findings confirmed the physical interaction between CALS7 and PDLP6 (also PDLP5).

To further characterize the functions of *CALS7*, we examined the genetic interaction between *PDLP6* and *CALS7*. Using the same strategy mentioned above, we isolated *PDLP6-HF cals7* and *PDLP6-HF CALS7* (Supplementary Fig. S6, C and D). *cals7* mutant largely suppresses plant growth and starch accumulation phenotypes of *PDLP6-HF* (Fig. 7G; Supplementary Fig. S6, B and F), suggesting the genetic interaction between the 2 genes. Our findings suggest that SUS6 and CALS7 function with PDLP6, regulating callose accumulation in the vasculature, likely in SE.

## Discussion

This report presents findings that 2 PDLP proteins, PDLP5 and PDLP6, regulate plasmodesmal function at different cell interfaces. We draw the conclusion based on the following evidence: (i) PDLP5 is expressed in epidermal cells and likely also in mesophyll cells, whereas PDLP6 is expressed predominantly in phloem (Fig. 3); (ii) the overexpression of PDLP5 increases callose accumulation at plasmodesmata between epidermal cells and mesophyll cells, whereas the overexpression of PDLP6 increases callose accumulation mainly in vascular tissues (Fig. 4); and (iii) the overexpression of PDLP5-HF and PDLP6-HF predominantly affects plasmodesmal function (Fig. 5) and starch hyperaccumulation (Fig. 2) at distinct cells and cell–cell interfaces. Our studies led to an intriguing observation that the ubiquitous overexpression of PDLP5 and PDLP6 impacts the plasmodesmal callose accumulation and function at distinct cell–cell interfaces. Using a combination of cell biology, biochemical, and genetic analyses, we provided evidence showing that PDLP6 functions with SUS6 and CALS7 to regulate the plasmodesmal function in phloem.

We hypothesize that for PDLP6 to function predominantly in phloem, 2 key regulations are necessary: (i) transcriptional regulation of *PDLP6* expression in phloem and (ii) expression of PDLP6's functional partners in the same cell types. A previous report has shown that different members of *PDLP* transcripts were detected in distinct Arabidopsis leaf cell types, with PDLP6 identified in PP cells (Kim et al. 2021). We detected PDLP6-YFP fusion protein in phloem, including PP cells, CC, and SE (Fig. 3, C to G). Conversely, PDLP5 was primarily observed in epidermal cells (Fig. 3, A and B). These findings support the transcriptional regulation of PDLPs to express them predominantly in distinct cell types. Using a PL assay, we identified SUS6 as a potential functional partner of PDLP6 (Fig. 6E). Biochemical approaches confirmed the physical interaction between PDLP6 and SUS6 (Fig. 7, D and E), and genetic analysis established the dependence of PDLP6 function on SUS6 (Fig. 7F). Additionally, we demonstrated physical and genetic interactions between PDLP6 and CALS7 (Fig. 7, D, E, and G). Given that SUS6 and CALS7 are predominantly expressed in the phloem (Barratt et al. 2011; Xie et al. 2011; Yao et al. 2020; Kalmbach et al. 2023), our findings suggest that PDLP6 functions with SUS6 and CALS7 to regulate plasmodesmal function in the vasculature. In *PDLP6-HF* transgenic plants, PDLP6 was likely ubiquitously expressed in every cell (Supplementary Fig. S4). However, the ubiquitous expression of PDLP6 did not affect callose accumulation (Fig. 4) and plasmodesmal function (Fig. 5) in epidermal and mesophyll cells, possibly due to the absence of SUS6 and CALS7 or other functional partners of PDLP6 in these cells.

We consider PDLP5 and PDLP6 functionally equivalent in promoting callose accumulation at plasmodesmata. Due to the unknown biochemical activity of PDLPs, we can’t determine whether PDLP5 and PDLP6 are biochemically equivalent. However, PDLP5 and PDLP6 could be molecularly distinct, possibly interacting with different functional partners in distinct cell types to exert their function to regulate callose accumulation. While we detected the physical interaction among PDLP5, SUS6, and CALS7 using co-IP and in vitro pull-down assays (Supplementary Fig. S7), the overexpression of PDLP5 does not appear to promote callose accumulation in the vasculature (Fig. 4). If the overexpressed PDLP5 can interact with SUS6 and CALS7 in planta, the physical interaction alone may not be sufficient for PDLP5 to function with SUS6 and CALS7 to regulate callose accumulation in the vasculature. Given that PDLP5 is predominantly expressed in nonoverlapping cell types compared with SUS6 and CALS7, the observed physical interactions may not hold biological relevance under their native conditions. PDLP5 might rely on the cytosolic invertase (CINV) pathway, involving multiple steps to convert sucrose into UDPG for callose synthesis to regulate callose accumulation in the epidermal and mesophyll cells.

Recent reports showed that none of the SUSs is ubiquitously expressed in Arabidopsis (Yao et al. 2020). SUS5 and SUS6 express predominantly in SE (Yao et al. 2020). In line with the tissue type–specific expression, *sus5;6* double mutant exhibits a lower level of callose in SE (Barratt et al. 2009). Here, we demonstrated the role of SUS6 in functioning together with PDLP6 and CALS7 (Fig. 7). Further research is needed to determine the role of SUS5 in functioning with PDLP6 and CALS7 to regulate callose accumulation and plasmodesmal function in SE.

In Arabidopsis, 2 metabolic pathways have been proposed for generating UDPG, which is used as a glucose donor for callose and cellulose biosynthesis (Kleczkowski et al. 2004). SUS can directly convert sucrose to UDPG and fructose. The pathway is energetically more economical; however, SUSs do not play a major role in UDPG production during cellulose biosynthesis (Wang et al. 2022). Since SUSs do not express in most tissues (Yao et al. 2020), including epidermal and mesophyll cells, a SUS-dependent UDPG production pathway might not be a dominant metabolic pathway in most cell types. Alternatively, UDPG can be generated through a CINV-dependent pathway (Barratt et al. 2009; Barnes and Anderson 2018). In addition to CINV, hexokinase, phosphoglucoisomerase, and UDP-glucose pyrophosphorylase (UGP) are required to generate UDPG from sucrose (Kleczkowski et al. 2004). During cellulose biosynthesis, a CINV-dependent pathway is considered the major metabolic pathway in generating UDPG (Barnes and Anderson 2018). In this pathway, UGP converts glucose-1-phosphate into UDPG. Further research is needed to determine whether the CINV pathway is crucial for a PDLP5-containing callose synthase complex in epidermal and mesophyll cells.

Interfering with the movement of molecules through plasmodesmata can severely impact plant growth and development (Kim et al. 2005; Thomas et al. 2008; Guseman et al. 2010; Zavaliev et al. 2010; Lee et al. 2011; Benitez-Alfonso et al. 2013; Dettmer et al. 2014; Brunkard et al. 2020). For instance, the overexpression of the plasmodesmal-associated class 1 reversibly glycosylated polypeptide in Arabidopsis results in suppressed plasmodesmal function, inhibited plant growth, and a hyperaccumulation of starch in *Nicotiana tabacum* (Zavaliev et al. 2010). Here, we reported that the overexpression of PDLP6 leads to delayed plant growth in Arabidopsis (Fig. 1A). A similar effect has been reported in Arabidopsis transgenic plants overexpressing PDLP1 (Thomas et al. 2008) and PDLP5 (Lee et al. 2011; Supplementary Fig. S1A); however, it is unknown whether the overexpression of PDLP1 also leads to starch hyperaccumulation. We further demonstrated that the delayed plant growth phenotype correlates with the PDLP protein expression level (Fig. 1B; Supplementary Fig. S1B). We thus hypothesize that an extensive screen of Arabidopsis transgenic lines overexpressing different PDLPs will identify transgenic plants with the delayed plant growth phenotype. In combination with determining cell type–specific expression of PDLPs, further characterization of the transgenic plants will uncover the function of the PDLPs in regulating plasmodesmata at distinct cell interfaces.

This study demonstrated the application of an enzyme-catalyzed PL assay to identify plasmodesmal proteins. As shown in Supplementary Data Set 1, we identified many proteins enriched significantly by PDLP5, PDLP6, or MCTP3. The identification of candidate proteins with potential functions at plasmodesmata greatly boosts confidence in confirming and determining the roles of other candidate proteins with no clear functions at plasmodesmata (Supplementary Data Set 1). We identified several putative functional partners of PDLP5 and PDLP6, including LEUCINE-RICH REPEATS RECEPTOR-LIKE KINASEs and CYSTEINE-RICH RECEPTOR-LIKE KINASEs (Supplementary Data Set 1). It is tantalizing to hypothesize that the plasmodesmata-localized receptor-like kinases regulate the activity of callose synthase complexes through phosphorylation. Despite using a *UBQ10* promoter, which likely expresses the PDLPs ubiquitously, we identified several members of PIPs (AQUAPORINs) as specific functional partners of PDLP5 (Fig. 6E). Recent findings showed that PIP1;4 and PIP2;1 can transport hydrogen peroxide (Rodrigues et al. 2017; Wang et al. 2020; Groszmann et al. 2023). In addition, *pdlp5* and *pip2;1* mutants cannot trigger reactive oxygen species waves in systemic tissues in response to abiotic stress (Fichman et al. 2021). Given the functional roles of PDLP5 and reactive oxygen species in plant immunity, functional characterization of the PIPs has the potential to reveal the functions of PDLP5–PIP complexes in regulating plasmodesmata during microbial defense. To date, several attempts have been made to catalog plasmodesmal proteome from purified plasmodesmata and co-IP assays (Fernandez-Calvino et al. 2011; Kraner et al. 2017; Leijon et al. 2018; Brault et al. 2019). Our findings demonstrated that the PL assay is another powerful tool for identifying plasmodesmal proteins to better understand plasmodesmal biology.

Recent advancements in single-cell transcriptomic, proteomic, and metabolomic techniques have provided unprecedented insights into plant cellular processes. However, our knowledge of the regulatory mechanisms governing communication between distinct cell types is still limited. This study identified a protein complex that regulates plasmodesmal function in the vasculature. To investigate the regulation of plant cell-to-cell communication at these interfaces, we established an experimental pipeline that combines cellular, genetic, biochemical, and histological approaches. Our findings not only shed light on the intricate regulation of communication channels in multicellular organisms but also open new avenues of research in understanding how these organisms operate as cohesive units.

## Materials and methods

### Plant material, growth conditions, transformation, and plant selection

Arabidopsis (*A. thaliana*) and *N. benthamiana* plants were grown at 22 °C with 50% humidity and irradiated with 110 *µ*mol/m^2^/s white light (fluorescent bulbs) for 16 h per day. To grow plants under high light intensity, plants were irradiated with 200 *µ*mol/m^2^/s white light. Arabidopsis T-DNA insertion mutants, *cals7* (SALK_048921) and *pdlp5* (SAIL_46_E06.v1), were obtained from ABRC (Columbus, OH). *sus56* (SALK_152944 and SALK_107491) is a gift from Dr. Alison M. Smith's lab. *pdlp6-1* and *pdlp6-2* were generated using CRISPR/Cas9 technology in this study. Primers used for mutant genotyping are listed in Supplementary Table S1. Transgenic Arabidopsis plants were generated using the simplified transformation method (https://plantpath.wisc.edu/simplified-arabidopsis-transformation-protocol/). To select transgenic plants harboring transgenes containing the hygromycin resistance gene, T_1_ seeds were germinated on a 1/2 Linsmaier and Skoog (LS) medium containing 25 *µ*g/mL hygromycin. For those harboring transgenes containing the glufosinate resistance gene, T_1_ seeds were germinated on soil and sprayed with 0.1% (*v/v*) Finale herbicide (Bayer) and 0.05% (*v/v*) Silwet L-77 (PhytoTech) around 10 d after germination. The T_2_ or T_3_ plants were selected on a 1/2 LS medium containing 10 *µ*g/mL Glufosinate-ammonium (MilliporeSigma).

### Gene cloning and plasmid construction

If not specified otherwise, constructs generated in this work used a standard Gateway cloning system (Invitrogen). Constructs with an MBP tag were cloned by restriction digestion and NEBuilder HiFi DNA Assembly (New England Biolabs). Coding sequences, promoters, or genomic DNA fragments were amplified with Gateway-compatible primers from cDNA synthesized from total RNA extracted from wild-type Col-0 seedlings or total genomic DNA extracted from Col-0 using Phusion High-Fidelity DNA Polymerase (Thermo Fisher). PCR fragments were first cloned into the pDONR 207 entry vector and subsequently cloned into different destination vectors. CRISPR-P 2.0 (Liu et al. 2017) was used to design the guide sequence for PDLP6 (http://crispr.hzau.edu.cn/CRISPR2/). All primers and vectors used for cloning are listed in Supplementary Tables S2 and S3. In this study, we reported the following constructs: *Pro35S:PDLP5-HF*, *Pro35S:PDLP6-HF*, *Pro35S:HF-YFP*, *Pro35S:PDLP5-YFP*, *Pro35S:PDLP6-YFP*, *Pro35S:PDLP7-YFP*, *ProPDLP5:PDLP5-YFP*, *ProPDLP6:PDLP6-YFP*, *ProPDLP5:GUS*, *ProPDLP6:GUS*, *pKIR1.1-CRISPR-PDLP6*, *ProPDLP6:PDLP6-YFP-3′UTR-Ter*, *Pro35S:SUS6-sfGFP*, *ProSWEET13:SWEET13-mCherry*, *ProSUC2:PP2A1-mCherry*, *ProSEOR2:SEOR2-mCherry*, *pET-MBP-SUS6*, *pET-MBP-CALS1-CL*, *pET-MBP-CALS7-CL*, *ProPDLP6:1xYFP*, *ProUBQ10:PDLP5-TbID-3xFlag*, *ProUBQ10:PDLP6-TbID-3xFlag*, *ProUBQ10: 3xFlag-TbID-MCTP3*, *ProUBQ10:PDLP5-TbID-EYFP-3xFlag*, *ProUBQ10:PDLP6-TbID-EYFP-3xFlag*, and *ProUBQ10:3xFlag-EYFP-TbID-MCTP3*.

### Whole tissue starch staining

The aerial portion of 3- to 4-wk-old Arabidopsis plants grown under regular light was harvested at the end of the night. For high light-treated plants, 4-wk-old Arabidopsis plants grown under a regular light intensity (110 *µ*mol/m^2^/s) were treated with higher light intensity (200 *µ*mol/m^2^/s) for 7 to 10 d. The inflorescence was removed, and the rosette leaves were decolored with 95% ethanol. The samples were then washed with ddH_2_O and stained with Lugol's iodine solution (Sigma-Aldrich) for 5 min and rinsed with ddH_2_O. The images were captured with a Canon camera 1 to 2 h after destaining.

### CFDA feeding assay

Seedlings were grown vertically on 1/2 LS medium for 7 to 10 d. A strip of new 1/2 LS medium was removed, and seedlings were transferred with only root and part of hypocotyl on the medium. One cotyledon from each seedling was half-clipped. Twenty microliters of 1 mm CFDA (100 mm stock in DMSO) was applied to the clipped cotyledons. Seedlings were incubated in darkness for 1 h, and fluorescent signals in root tips were detected using confocal microscopy. Propidium iodide (PI) was used as a root cell wall stain. Roots were immersed in 10 *μ*g/mL of PI solution for 1 min and then washed in ddH_2_O before being mounted onto slides for confocal imaging.

### Tissue sectioning and starch staining

Leaf punch samples were collected and fixed in FAA fixative (5% formaldehyde, 5% glacial acetic acid, 50% ethyl alcohol). Samples were dehydrated through graded ethanol series (70, 85, 95, and 100%) for 3 to 6 h for each concentration. Samples were infiltrated into LR White hard grade resin (Electron Microscopy Sciences) and polymerized at 55 °C for 48 h. Sections were made using a Leica UC6 ultramicrotome at 1.5 *µ*m thickness. Sections were stained for nonsoluble polysaccharides as the following: slides with sections were immersed in periodic acid for 5 min, rinsed in distilled water for 5 min, stained in Schiff's reagent (Electron Microscopy Sciences) for 10 min, rinsed in running tap water for 5 min, and air-dried. Dry slides were coverslipped using Permount mounting media (Fisher Scientific). Images were captured using Axio Imager A2.

### TEM

Leaves were dissected using a leaf punch, and samples were fixed with 3% glutaraldehyde (*w/v*) and 1% paraformaldehyde (*w/v*) in 0.1 M cacodylate at 4 °C. Samples were rinsed 3 times in 0.1 M cacodylate buffer at room temperature. The samples were post-fixed in 1% osmium tetroxide in 0.1 M cacodylate for 1 h, followed by a 5-min wash in dH_2_O and en bloc staining with 2% uranyl acetate for 1 h. The samples were then dehydrated in a graded ethanol series, cleared with ultrapure acetone, and infiltrated/embedded using SPURR's epoxy resin (Electron Microscopy Sciences). Resin blocks were polymerized for 48 h at 70 °C. Thick and ultrathin sections were made using a Leica UC6 ultramicrotome. Ultrathin sections (60 to 70 nm) were collected onto copper grids, and images were captured using a JEOL JSM2100 scanning and TEM.

### Anthocyanin extraction and quantification

Fresh tissues were frozen with liquid nitrogen and homogenized with 1600 miniG (SPEX). One milliliter of extraction buffer (45% methanol and 5% acetic acid) was added to the homogenized tissues. The samples were incubated at 4 °C for 30 min and centrifuged at 14,000 × g for 5 min at room temperature. The supernatant was transferred into a new 1.5 mL tube. A total of 200 *μ*L of the supernatant was pipetted to 96-well plates, and the absorbances at 530 and 657 nm were determined using a plate reader (SpectraMax iD3). Anthocyanin content was calculated by Abs_530_ − (0.25 × Abs_657_)/g fresh weight.

### Starch and soluble sugar extraction and quantification

The whole rosettes from 5-wk-old Arabidopsis plants treated with high light for a week were collected at the end of the night and immediately frozen in liquid nitrogen before being stored at −80 °C until quantification. Soluble sugar and starch were extracted according to Leach and Braun (2016). A methanol/chloroform/water (MCW)-based extraction was performed to isolate soluble sugars. Samples were homogenized in liquid nitrogen, and 100 mg of powder was transferred into 1.5 mL Eppendorf tubes. One milliliter of MCW extraction buffer was added. The samples were vortexed briefly, incubated in a 50 °C water bath for 30 min, and centrifuged at 14,000 × *g* for 5 min at room temperature. The supernatant was transferred into a 15 mL conical tube and stored on ice. Repeat the extraction process twice, and the supernatant was combined in the 15 mL conical tube. The pellet was saved for starch determination. Water of 0.6 volumes was added to the 15 mL conical tube. The samples were vortexed and centrifuged at 4,650 × *g* for 5 min at room temperature. The aqueous (top) phase containing the soluble sugars was pipetted into a 1.5 mL Eppendorf tube and stored at −20 °C until measurement. Sucrose concentration was determined using Megazyme kit (catalog no. K-SUFRG) following manufacturer instructions. Starch was solubilized from the MCW-extracted tissue pellet and enzymatically degraded into glucose for quantification. The pellet was resuspended in 330 *μ*L of DMSO and heated at 100 °C for 5 min to solubilize and gelatinize the starch. Fifty microliters of the slurry was diluted by 950 *μ*L of 100 mm sodium acetate (pH 5.0). α-Amylase (30 U) was added, and the samples were vortexed briefly, heated at 100 °C for 15 min, and cooled at 50 °C for 3 min. Amyloglucosidase (66 U) was added, and the samples were vortexed briefly, incubated at 50 °C for 1 h, and centrifuged at 14,000 × *g* for 5 min at room temperature. The supernatant containing starch-derived glucose was transferred into a new 1.5 mL Eppendorf tube. Starch concentration was determined using Megazyme kit (catalog no. K-TSTA-50A) following manufacturer instructions.

### Aniline blue staining

Mature leaves from 5-wk-old Arabidopsis plants were infiltrated with 0.1 mg/mL aniline blue in 0.01 M K_3_PO_4_ (pH 12) to determine callose accumulation in epidermal and mesophyll cells. Ten-day-old seedlings were vacuum-infiltrated with 0.1 mg/mL of aniline blue in 0.01 M K_3_PO_4_ (pH 12) to detect callose accumulation in vasculatures. Mature leaves from 6-wk-old *N. benthamiana* were infiltrated with 0.1 mg/mL of aniline blue in 1× PBS buffer (pH 7.4) to detect callose accumulation in epidermal cells. The leaf tissues were stained for ∼5 min prior to imaging. Stained tissues were imaged using Zeiss LSM 700 Laser Scanning Confocal System. Callose in mature leaves was quantified using FIJI. For epidermis, images were converted from lsm to tiff. Images of 8 bits were used for analysis. Black and white images highlighting callose were created by Auto Threshold, which was set by RenyiEntropy white method. A Particle Analysis tool was used to outline callose with sizes from 0.10 to 20 *µ*m^2^ and circularity from 0.15 to 1.00. Quantitative numerical values in square micrometer were then exported. For mesophyll cells, an aniline blue–stained callose area was manually selected, and the signal intensity was determined by measuring integrated density. For vasculatures, the whole vasculature area was manually selected, and the mean signal intensity was determined by measuring integrated density and areas.

### GUS activity staining

GUS staining was performed as previously described (Li et al. 2016) with minor modifications. Mature leaves and small seedlings were immersed in GUS solution (100 mm sodium phosphate buffer [pH 7.0], 10 mm Na_2_EDTA, 1 mm K_3_ [Fe(CN)_6_], 1 mm K_4_ [Fe(CN)_6_], 0.1% Triton X-100, and 1 mm X-Gluc). Samples were vacuumed for 10 to 40 min, followed by incubation in darkness at 37 °C for 2 to 16 h. After staining, samples were destained in 75% ethanol. For imaging transverse leaf sections, GUS-stained leaves were embedded in 3% agarose and sectioned with a vibrating blade microtome (Leica VT1000 S). Images were taken using ZEISS Axio Observer.

### ClearSee

ClearSee was performed as previously described (Kurihara et al. 2015). ClearSee solutions contain 15% (*w/v*) sodium deoxycholate, 25% (*w/v*) urea, and 10% (*w/v*) xylitol powder in water. Leaves were fixed with 4% (*w/v*) paraformaldehyde in 1× PBS (pH 7.4) for 2 h under vacuum until the samples became fully water-soaked. The fixed samples were washed twice in 1× PBS pH7.4 and then cleared with ClearSee solutions at room temperature for 1 wk or more until the samples became clear. ClearSee solutions were changed every 2 d.

### Confocal imaging

All confocal images were captured with a confocal laser scanning microscope (Zeiss LSM 700). A small piece of tissue was mounted with water on a glass slide. For leaf tissues, the abaxial side was imaged. YFP was excited at 488 nm and emission was collected over 510 to 550 nm using SP555. Aniline blue–stained callose was excited at 405 nm and emission was collected over 420 to 480 nm using SP490. CF was excited at 488 nm and emission was collected over 505 to 545 nm using SP555. PI was excited at 555 nm and emission was collected using SP640.

### Immunoblot analyses

Arabidopsis leaves were frozen with liquid nitrogen and homogenized with 1600 miniG (SPEX). Protein extraction buffer (60 mm Tris–HCl [pH 8.8], 2% [*v/v*] glycerol, 0.13 mm EDTA [pH 8.0], and 1× protease inhibitor cocktail complete from Roche) was added to the homogenized tissues (100 *µ*L/10 mg). The samples were vortexed for 30 s, heated at 70 °C for 10 min, and centrifuged at 13,000 × *g* for 5 min at room temperature. The supernatants were then transferred to new tubes. For SDS-PAGE analysis, 10 *µ*L of the extract in 1× Laemmli sample buffer (Bio-Rad) was separated on 4% to 15% Mini-PROTEAN TGX precast protein gel (Bio-Rad). The separated proteins were transferred to a polyvinylidene fluoride membrane (Bio-Rad) using a Trans-Blot Turbo Transfer System RTA transfer kit following the manufacturer's instructions (Bio-Rad). The membrane was incubated in a blocking buffer (3% [*v/v*] BSA, 50 mm Tris base, 150 mM NaCl, 0.05% [*v/v*] Tween 20 [pH 8.0]) at room temperature for 1 h and then incubated overnight with a 1:10,000 dilution of an α-GFP (Abcam, catalog no. ab290), α-Flag-HRP (Sigma-Aldrich, catalog no. A8592), α-Streptavidin-HRP antibody (Abcam, catalog no. ab7403), or α-MBP (NEB, catalog no. E8032S) at 4 °C. The membrane was washed 4 times with 1× TBST (50 mm Tris base, 150 mm NaCl, 0.05% [*v/v*] Tween 20 [pH 8.0]) for 10 min. For an α-GFP or α-MBP, 1:20,000 goat antirabbit IgG (Thermo Fisher Scientific, catalog no. 31460) or sheep antimouse IgG (Cytiva, catalog no. NA931) was used as a secondary antibody. The membrane was washed 4 times with 1× TBST (50 mm Tris base, 150 mm NaCl, 0.05% [*v/v*] Tween 20 [pH 8.0]) for 10 min. The signals were visualized with SuperSignal West Dura Extended Duration Substrate (Pierce Biotechnology) or with Clarity (Max) Western ECL Substrate (Bio-Rad).

### Transient overexpression for subcellular localization and Co-IP

*Agrobacterium tumefaciens* GV3101 harboring the plasmid of interest was adjusted to an optical density of A_600_ 0.1 using sterilized ddH_2_O and infiltrated into 6-wk-old *N. benthamiana* leaves. Infiltrated tissues were subjected to live-cell imaging or co-IP assay 2 d after infiltration. Co-IP assay was performed as previously described (Aung et al. 2020). One gram fresh weight of tissues was ground in liquid nitrogen and lysed with 3 mL of radioimmunoprecipitation assay (RIPA) buffer (50 mm Tris–HCl [pH 7.5], 150 mm NaCl, 1% NP-40, 1% sodium deoxycholate, and 0.1% SDS with 1× complete protease inhibitor cocktail [Roche]) on a rotator at 4 °C for 1 h. The samples were centrifuged at 13,000 × *g* for 10 mins, filtered with 2 layers of Miracloth, and centrifuged again to remove cell debris. The supernatants were served as input controls. Twenty microliters of 4× SDS loading buffer (Bio-Rad) was added into input samples and heated at 95 °C for 5 min. Fifteen microliters of GFP-Trap_A (ChromoTek) was washed 3 times with RIPA buffer and added into the supernatant. The samples were mixed on a rotator at 4 °C for 1 h. The agarose beads were spun down at 100 g for 1 min and washed 4 times with RIPA buffer. Proteins co-immunoprecipitated with the YFP fusion protein were eluted by adding 60 *μ*L of 1× SDS loading buffer and heating at 95 °C for 5 min or 70 °C for 10 min. The protein samples were analyzed by immunoblot assay.

### Recombinant protein purification

*Escherichia coli* Rosetta cells were transformed with pET constructs containing MBP, MBP-SUS6, or MBP-CalS7-CL. The expression of the recombinant proteins was induced using 300 *μ*m isopropyl ß-D-1-thiogalactopyranoside. Following overnight culture (500 mL), cells were lysed in 40 mL of MBP lysis buffer (20 mm Tris [pH 7.4], 200 mm NaCl, 1 mm EDTA, 0.5 mg/mL lysozyme, 1 mm PMSF, 10 mm β-Me), and the samples were kept on ice for 1 h. Subsequently, 400 *μ*L of Triton X was added to each sample, and sonication was performed using a Qsonica Q125 Sonicator. The lysates were centrifuged at 10,000 rpm for 10 min at 4 °C, and the supernatant was filtered through layers of Miracloth. To purify MBP fusion proteins, 250 *μ*L of preequilibrated amylose resin beads was added to the supernatant and rotated at 4 °C for 1 h. The beads were centrifuged at 1,500 rpm for 2 min and washed twice with MBP lysis buffer containing Triton X-100 (0.1%). The beads were washed 2 times with MBP lysis buffer without Triton X-100. Finally, the recombinant proteins were eluted with 250 *μ*L of elution buffer containing 10 mm maltose by rotating at room temperature for 10 min.

### In vitro pull-down assay

The biotinylated C-terminal tail peptides of PDLP5 (GKCCRKLQDEKWCK), PDLP6 (AKSCERGKGGK), and PDLP7 (RGVCSRGGDFSILHSFTLI) were obtained from PEPTIDE 2.0. To perform binding assays, 1 *μ*g of the biotinylated peptides was combined with 1 *μ*g of MBP, MBP-SUS6, or MBP-CALS7-CL fusion proteins in 300 *μ*L of binding buffer (50 mm Tris [pH 7.5], 150 mm NaCl, and 0.05% NP-40). Negative controls without peptides were included. The mixtures were rotated at 4 °C overnight. Dynabeads MyOne Streptavidin C1 (Thermo Fisher) was washed 3 times with binding buffer, and 20 *μ*L of bead slurry was used for each sample. The samples were rotated at 4 °C for 3 h. Subsequently, the beads were washed 3 times with 1 mL of binding buffer, and the beads were resuspended in 50 *μ*L of 2× SDS sample buffer. The protein samples were heated at 70 °C for 10 min and subjected to an immunoblot assay.

### Statistical analysis

Column plots were created using GraphPad Prism. Box plots were created with an online software (https://huygens.science.uva.nl/PlotsOfData/). Mann–Whitney *U* test (www.socscistatistics.com/tests/mannwhitney/default2.aspx), Student's *t*-test (https://www.socscistatistics.com/tests/studentttest/default2.aspx), or 1-way ANOVA was performed for testing the statistical significance of differences. A summary of statistical analyses is provided in Supplementary Data Set 2.

### PL

The PL assay was performed according to Mair et al. (2019) with minor modifications. Three independent transgenic events from each transgenic line were subjected to biotin labeling and streptavidin enrichment. Fourteen-day-old seedlings were carefully removed from 1/2 LS medium, transferred into 40 mL of a 50 *μ*m biotin solution, and incubated at room temperature for 3 h. The biotin solution was then removed, and seedlings were quickly rinsed with ice-cold water 3 times. The samples were homogenized using a pestle and mortar in liquid nitrogen. Around 1.5 mL of leaf powder was resuspended with 2 mL of RIPA lysis buffer (50 mm Tris-HCl [pH 7.5], 150 mm NaCl, 1% NP-40, 1% sodium deoxycholate, and 0.1% SDS with 1× complete protease inhibitor cocktail [Roche]). The samples were vortexed to mix and incubate at 4 °C on a rotator for 10 min. To digest cell walls and DNA/RNA, the samples were incubated with 0.5 *μ*L of Lysonase (Millipore) on a rotator at 4 °C for 15 min. The samples were centrifuged at 15,000 × *g* for 10 mins at 4 °C. The clear supernatant was applied to a PD-10 desalting column to remove excess free biotin using gravity following the manufacturer's instructions. A total of 2.5 mL of the protein extract was loaded onto the desalting column. Proteins were then eluted with 3.5 mL of equilibration buffer (RIPA buffer without 1× complete protease inhibitor cocktail). The desalted protein extracts were quantified by Bradford assay, and a complete protease inhibitor cocktail was added to each sample to reach final concentrations of 1× complete.

### Affinity purification of biotinylated proteins

To enrich biotinylated proteins, 150 *µ*L streptavidin-coated beads (Dynabeads MyOne Streptavidin C1 beads) prewashed with extraction buffer were mixed with protein extracts and incubated on a rotator at 4 °C overnight. The Streptavidin beads were then washed with 1 mL of the following solutions for 2 min each: 2× cold extraction buffer, 1× cold 1 m KCl, 1× cold 100 mm Na_2_CO_3_, 1× 2 m urea in 10 mm Tris (pH 7.5), and 6× 50 mm Tris (pH 7.5). The beads were resuspended in 200 *μ*L of 50 mm Tris (pH 7.5) and stored at −80 °C for subsequent analysis until the next step.

### MS sample preparation

Biotinylated proteins on Streptavidin beads eluted from beads by incubation at 95 °C for 10 min in 1× S-Trap lysis buffer (5% SDS, 50 mm TEAB, pH 8.5) supplemented with 12.5 mm biotin. Eluted samples were subjected to S-Trap sample processing technology (catalog no. C02-micro-80, ProtiFi, USA), following manufacturer protocol. Samples were reduced in 2 mm TCEP, alkylated in 50 mm iodoacetamide, and digested into peptides at 37 °C in 1 round of overnight incubation with 1 *µ*g of trypsin and a second incubation of 4 h with 0.1 *µ*g trypsin plus 0.1 *µ*g Lys-C. Peptides were further desalted using Sep-Pack C18 columns (Waters). Tandem mass tag (TMT, Thermo Scientific) labeling was performed on purified peptides from each sample as previously reported (Song et al. 2020). TMT labeling reaction was stopped using 5% hydroxylamine, and the quenched samples were then pooled. Pooled samples were subjected to high pH fractionation using Pierce High pH Reversed-Phase Peptide Fractionation Kit (Thermo Scientific) following manufacturer instructions. The obtained 8 fractions were further concatenated (pooled) as follows: fraction 1 with fraction 5, fraction 2 with fraction 6, fraction 3 with fraction 7, and fraction 4 with fraction 8. Samples were dried in a SpeedVac and resuspended in 0.1% TFA in Optima grade H_2_O (Fisher).

### LC-MS/MS

Chromatography was performed on a Thermo UltiMate 3000 UHPLC RSLCnano. Peptides were desalted and concentrated on a PepMap100 trap column (300 *µ*m i.d. × 5 mm, 5 *µ*m C18, 100 Å µ-Precolumn, Thermo Scientific) at a flow rate of 10 *µ*L/min. Sample separation was performed on a 200 cm Micro-Pillar Array Column (µ-PAC, PharmaFluidics) with a flow rate of ∼300 nL/min over a 150 min reverse phase gradient (80% ACN in 0.1% FA from 1% to 15% over 5 min, from 15% to 20.8% over 20 min, from 20.8% to 43.8% over 80 min, and from 43.8% to 99.0% in 11 min and kept at 99.0% for 5 min). Eluted peptides were analyzed using a Thermo Scientific Q-Exactive Plus high-resolution quadrupole Orbitrap mass spectrometer, which was directly coupled to the UHPLC. Data-dependent acquisition was obtained using Xcalibur 4.0 software in positive ion mode with a spray voltage of 2.3 kV and a capillary temperature of 275 °C and an RF of 60. MS1 spectra were measured at a resolution of 70,000 and an automatic gain control (AGC) of 3e6 with a maximum ion time of 100 ms and a mass range of 400 to 2000 m/z. Up to 15 MS2 were triggered at a resolution of 35,000. A fixed first mass of 115 m/z, an AGC of 1e5 with a maximum ion time of 50 ms, a normalized collision energy of 33, and an isolation window of 1.3 m/z were used. Charge exclusion was set to unassigned, 1, 5 to 8, and >8. MS1 that triggered MS2 scans were dynamically excluded for 25 s.

### Proteomic data analysis

Raw data were analyzed using MaxQuant version 2.1.0.0 (Cox and Mann 2008). Spectra were searched, using the Andromeda search engine (Cox et al. 2011), against *A. thaliana* TAIR10 annotation (www.arabidopsis.org). The proteome files were complemented with reverse decoy sequences and common contaminants by MaxQuant. Carbamidomethyl cysteine was set as a fixed modification, while methionine oxidation and protein N-terminal acetylation were set as variable modifications. The sample type was set to “Reporter Ion MS2” with “TMT18plex” selected for both lysine and N-termini. TMT batch–specific correction factors were configured in the MaxQuant modifications tab (TMT lot no. XA338617). Digestion parameters were set to “specific” and “Trypsin/P; LysC.” Up to 2 missed cleavages were allowed. A false discovery rate, calculated in MaxQuant using a target-decoy strategy (Elias and Gygi 2007), less than 0.01 at both the peptide spectral match and protein identification level was required. The match-between-run feature of MaxQuant was not utilized. Statistical analysis on the MaxQuant output was performed using the TMT-NEAT Analysis Pipeline (Clark et al. 2021). Differential expression was assessed by 2-sample *t*-test, and Benjamini–Hochberg (BH) *P*-value adjustment was used for multiple test correction. We utilized MS2-based isobaric (TMT/iTRAQ) reporter ion quantification in our PL assay, which demonstrates high precision but exhibits ratio compression, leading to an underrepresentation of the actual level of enrichment (Wühr et al. 2012; Savitski et al. 2013). To address this, proteins were called as significantly enriched interactors if they were under a false discovery rate cutoff of *q* < 0.1 and a log_2_FC > 0.2 or <−0.2. PCA was performed and visualized using the ggbiplot R package as part of the TMT-NEAT analysis pipeline. Volcano plots were generated using VolcaNoseR.

### Accession numbers

The Arabidopsis Genome Initiative locus identifiers for the genes studied in this article are as follows: *PDLP5* (At1g70690), *PDLP6* (At2g01660), *PDLP7* (At5G37660), *SUS5* (At5g37180), *SUS6* (At1G73370), *CALS1* (At1G05570), *CALS7* (At1G06590), *MCTP3* (At1g06490), and *UBQ10* (At4g05320). Germplasm identification numbers of the mutants studied in this work are as follows: *cals7* (SALK_048921), *sus5* (SALK_152944), and *sus6* (SALK_107491).

## Supplementary Material

koae166_Supplementary_Data

## Data Availability

The data underlying this article are available in the article and in its online supplementary material.

## Supplementary data

The following materials are available in the online version of this article.

**Supplementary Figure S1.** Growth phenotypes of *PDLP5-HF* and *PDLP6-HF*.

**Supplementary Figure S2.** Characterization of *pdlp6* mutants.

**Supplementary Figure S3.** Starch accumulation in mature leaves of different Arabidopsis genotypes.

**Supplementary Figure S4.** Ubiquitous expression of PDLP5-YFP and PDLP6-YFP fusion proteins in *Pro35S:PDLP5-YFP* and *Pro35S:PDLP6-YFP* transgenic plants.

**Supplementary Figure S5.** The expression of PDLP5-TbID, PDLP6-TbID, and TbID-MCTP3 and their enriched proteins.

**Supplementary Figure S6.** Determination of genetic interactions among PDLP6, SUS6, and CALS7.

**Supplementary Figure S7.** Interaction among PDLPs, SUS6, and CALSs.

**Supplementary Table S1.** Primers used for mutant genotyping in this study.

**Supplementary Table S2.** Primers used for plasmid construction in this study.

**Supplementary Table S3.** Plasmids used in this study.

**Supplementary Data Set 1.** TbID data sheets.

**Supplementary Data Set 2.** A summary of statistical analyses.

## Dive Curated Terms

The following phenotypic, genotypic, and functional terms are of significance to the work described in this paper:

PDLP8 Gramene: AT3G60720
PDLP8 Araport: AT3G60720

SUS1 Gramene: AT5G20830
SUS1 Araport: AT5G20830

CLV1 Gramene: AT1G75820
CLV1 Araport: AT1G75820

SUS6 Gramene: AT1G73370
SUS6 Araport: AT1G73370

SUC2 Gramene: AT1G22710
SUC2 Araport: AT1G22710

UGP Gramene: AT3G03250
UGP Araport: AT3G03250

anthocyanin content Planteome: TO:0000071

PDLP5 Gramene: AT1G70690
PDLP5 Araport: AT1G70690

PDLP6 Gramene: AT2G01660
PDLP6 Araport: AT2G01660

SUS5 Gramene: AT5G37180
SUS5 Araport: AT5G37180

CALS7 Gramene: AT1G06490
CALS7 Araport: AT1G06490

MCTP3 Gramene: AT3G57880
MCTP3 Araport: AT3G57880

PDLP7 Gramene: AT5G37660
PDLP7 Araport: AT5G37660

MCTP4 Gramene: AT1G51570
MCTP4 Araport: AT1G51570

RTNLB6 Gramene: AT3G61560
RTNLB6 Araport: AT3G61560

HVA22C Gramene: AT1G69700
HVA22C Araport: AT1G69700

REM1.2 Gramene: AT3G61260
REM1.2 Araport: AT3G61260

REM1.3 Gramene: AT2G45820
REM1.3 Araport: AT2G45820

SWEET11 Gramene: AT3G48740
SWEET11 Araport: AT3G48740

SWEET12 Gramene: AT5G23660
SWEET12 Araport: AT5G23660

PDLP1 Gramene: AT5G43980
PDLP1 Araport: AT5G43980

starch content Planteome: TO:0000696

callose deposition Gramene: GO:0052543
callose deposition Araport: GO:0052543

CALS1 Gramene: AT1G05570
CALS1 Araport: AT1G05570

UBQ10 Gramene: AT4G05320
UBQ10 Araport: AT4G05320
