# Supplementary material for: PLASMODESMATA-LOCATED PROTEIN 6 regulates plasmodesmal function in Arabidopsis vasculature
Source: Plant Cell. 2024 Jun 6;36(9):3543–61. doi: 10.1093/plcell/koae166 (PMC11371196; doi:10.1093/plcell/koae166)
Supplement: koae166_Supplementary_Data [file koae166_supplementary_data.zip › tpc.00745.2023-s06.pdf]

THE PLANT CELL

PLASMODESMATA-LOCATED PROTEIN 6 regulates plasmodesmal function in Arabidopsis vasculature

Zhongpeng Li, Su-Ling Liu, Christian Montes-Serey, Justin Walley, and Kyaw Aung

Review Timeline:

Submission Date:	11-Aug-2023
Editorial Decision:	19-Sep-2023
Revision Received:	02-Feb-2024
Editorial Decision:	26-Feb-2024
Revision Received:	24-Apr-2024
Editorial Decision:	29-Apr-2024
Revision Received:	10-May-2024
Accepted:	10-May-2024

Dr. Kyaw Aung
Iowa State University
Ames, Iowa

Dear Joe / Dr. Aung:

We have received reviews of your manuscript entitled "Plasmodesmata-located proteins regulate plasmodesmal function at specific cell interfaces in Arabidopsis." Thank you for submitting your best work to The Plant Cell.

The reviewers have collectively found your research to be intriguing, offering fresh insights into the functions of PD and PDLP, particularly in relation to sugar loading. However, they have raised a series of valid concerns. Specifically, reviewers have emphasized the need for additional genetic and biochemical data to strengthen the central conclusion. Furthermore, there are reservations about asserting a "cell type-specific role" for PDLP5 and PDLP6, among other conclusions that appear to lack sufficient support or may be subject to potential over-interpretation in the current manuscript.

Despite these concerns, the editorial board acknowledges that your paper has the potential to align with the scope and high-quality standards of TPC, provided substantial revisions are undertaken. We also understand the pressing need for a strong publication as an early-stage investigator. Therefore, we are open to considering a revised manuscript, contingent upon the acquisition of essential, definitive data to comprehensively address the concerns raised by the reviewers.

Given the nature of the comments, we are offering you 120 days from when we have issued this decision to complete the revision. This extension is a doubling of the standard revision timeframe we typically provide. If a revision is not returned within this time frame, and if you have not been granted an extension, we will withdraw the manuscript, which will leave you free to submit the work elsewhere. If you need an extension, we encourage you to contact us at any point before the 120 days have passed.

When you are ready to submit the revised version, please upload a highlighted copy that indicates all changes made in response to the editor and reviewer recommendations. Include an itemized list of all changes made in response to each of the reviewer's suggestions in the "Response to Reviewers" section; please note that reviewers do not have access to your cover letter, nor was this decision letter shared with them.

Thank you for the privilege of reviewing your work. We look forward to receiving your revised manuscript.

Sincerely,

The Plant Cell Editorial Board

Please note the following:

-The Plant Cell now requires authors to complete and submit an author revisions checklist upon submission of a revised manuscript. The aim of the checklist is to aid authors in preparing a high-quality manuscript, facilitate the review and assessment of revised manuscripts, and help to ensure that journal standards are maintained across the board. If your manuscript is accepted, the completed checklist will be published as supplemental material attached to the article online. Please download a copy of the checklist (pdf fillable form) at this link, for submission with your revised manuscript: https://tpc.msubmit.net/html/Author_Revisions_Checklist.pdf.

-Supplemental materials should be restricted to large datasets and tables, presentation of replicates, and validation of reagents, methods, or genotypes. Any data that are used to support the major claims must be in the main manuscript. Supplemental figure legends must indicate what figure in the main manuscript is supported by the supplemental data presented. Please justify how each of the supplemental figures meet the criteria.

-Sampling methods and nature of "biological replicates" should be described precisely (i.e. different plants, parts of plants, pooled tissue, independent pools of tissue, sampled at different times, etc), along with a clear description of and rationale for any statistical analyses conducted. The reader should know exactly what was sampled; what forms the basis of the calculation of any means and statistical parameters reported. This is also necessary to ensure that proper statistical analysis was conducted.

-Want to add this revision deadline to your calendar? Click below!

--- Reviewer comments:

Reviewer #1 (Comments for the Author):

In this manuscript, Li et al. share several exciting new discoveries about the enigmatic PDLP family and present a major methodological advance in how we can identify more proteins that localize to plasmodesmata using proximity labelling approaches. I think that many of the findings in this paper are impressive and will be of great interest to the PD field. My enthusiasm for the paper is lessened, unfortunately, for two main reasons. First, there are at least two distinct stories here, one about the phenotypes associated with PDLP6 and another about the TurboID method for identifying putative PDLP6 interactors, which can be connected but make this manuscript unwieldy, in my view. Second, and perhaps as a consequence of the first issue, the way this manuscript is written is often confusing if not outright misleading, often making claims that do not reflect the actual data as presented or that are not well-supported by the literature. Therefore, my recommendation is to substantially (substantially!!) rewrite the manuscript to narrowly focus on reporting the data presented here (not speculative ideas beyond the data presented here) clearly and coherently, and, in any case, to consider splitting the manuscript into two publications that will allow the experiments to be fully appreciated by readers.

I could go line by line, but that would be overwhelming for a review. Instead, I will highlight a couple of major issues, and use a couple of smaller points as examples; but I urge the authors to carefully go through each section and consider whether the claims made are central to sharing their experimental conclusions.

For example...
59 - starch is a product of photosynthesis, but not always a major one, that depends a lot on conditions and photoperiod
61 - starch is hydrolyzed to maltose, which is further hydrolyzed to glucose, and then metabolized to sucrose-I don't know why these details are here anyway, you could just say that starch is metabolized to sucrose
62 - source-to-sink transport happens during the days and the nights in Arabidopsis, not only at night. Sinks include not only non-photosynthetic tissues but also actively growing tissues, including young leaves (see papers from the last thirty years on this topic, from https://doi.org/10.1105/tpc.9.8.1381 through https://www.pnas.org/doi/abs/10.1073/pnas.1919196117).

Throughout the starch section of the results-if source-to-sink trafficking is disrupted, starch will likely accumulate, but the inverse statement isn't true: if starch has overaccumulated, it could be for many reasons completely unrelated to source/sink trafficking. This needs to be clearly stated throughout. (See line 186 for just one of several examples where this is overstated.) There also isn't much discussion about other sinks for sugars, such as vacuoles.

TEM is not a more reliable method for quantifying starch levels than Lugol's stain. An improved method would be to determine starch concentrations using standard enzymatic approaches. The TEM results are easily skewed by other factors, like differences in starch granule morphology or chloroplast physiology. For instance, there might be more overall starch but spread across fewer granules, making visualization by TEM challenging.

The path of sugars: PD transport is extremely restricted in source leaves compared to sink leaves (see , which has often been interpreted as evidence that PD trafficking does not play a major role in apoplastic loading (at least, until you get into the phloem). suc2 mutants are nearly lethal with extreme growth phenotypes under standard growth conditions. As originally described in the 2012 paper, sweet11;12 mutants have almost no phenotype under normal growth conditions and weak phenotypes when plants are subjected to 450 uE light (~4x the amount of light used by most labs), with the strongest phenotypes observed when plants are transferred from normal to extreme high-light environments. The suc2 data strongly support the apoplastic loading hypothesis. The sweet11;12 phenotype suggests that these facilitators at the PP - CC interface contribute to phloem loading and make important contributions under some physiological conditions, but do not support that this is the exclusive (or even primary) mechanism of phloem loading in Arabidopsis. Braun (2022) argues that this reflects some genetic redundancy, which might be true (maybe sweet13 is important, for instance), but it seems that an easier hypothesis is the one that was prevalent until the 2012 sweet paper: sugar concentrations are very high in plant apoplasts, and sugars are probably exported from cells other than the phloem parenchyma.
My point with all of this commentary is that, in reality, none of this actually matters for the authors to present their data clearly. Instead of focusing on debates about the route taken by sugars, the authors could just focus on explaining their results. I'm not even sure, to be honest, that the sweet11;12 story needs to be here at all unless you split this into two papers.

Following up on that point-why wasn't PDLP6-HF crossed to sweet11;12? Note that line 239 claims "synergistic"

impacts, but "synergy" should be reserved for effects of mutant combinations that are beyond those that could be predicted from either mutant alone, and a quantitative increase in starch accumulation when you mix these all together isn't something unexpected. Also, if PDLP6-HF is already decreasing sugar loading, why does the cher1 mutant enhance that phenotype (why aren't they epistatic)? Obviously we can come up with explanations, I'm just trying to illustrate a situation where the results don't have an obvious conclusion to be drawn, but the authors have picked on interpretation over others without clear explanation.

On the next section, Callose deposition: I don't think that the data strongly support the hypothesis that PDLP functions are determined by their cellular environment, as is repeatedly implied here and relies on the callose deposition data. One hypothesis to explain the results shown is that PDLP5 and PDLP6 control callose biosynthesis differently in different cell types. But, the authors already showed that the phenotypic effect of PDLP overexpression is remarkably sensitive to the degree of protein overexpression; therefore, another hypothesis is that the minor and inconsistent difference in callose accumulation between the PDLP5 and PDLP6 overexpression lines is, instead, about some subtle differences in the expression of these genes in those cell types in the different lines. Or this could indicate other differences in the functionality of PDLP5 and PDLP6. The callose deposition measured in roots doesn't seem to be localized to PD (at least to my eye), so I'm not sure exactly what we're looking at here.

Lastly, on the TurboID section: As is clearly shown in panel 5D, none of these proteins localize exclusively to PD, as expected. Instead, PDLP5 and PDLP6 are plasma-membrane proteins enriched at PD, and MCTP3 is an ER protein enriched at PD. This means that negative controls would be plasma membrane-localized proteins that are not enriched at PD (for PDLP5 and PDLP6) or ER proteins that are not enriched at PD (for MCTP3). Instead, MCTP3 is presented as a "control", which it is not-that's just a different experiment-and sfGFP is used as a "control" in immunoprecipitations, which it is not. Plasma membrane proteins tend to co-IP with each other, certainly more than they do with cytosolic proteins. It's no surprise that MCTP3-TbID labeled other MCTPs/synaptotagmins and Golgi-associated proteins, whereas PDLPs labelled plasma membrane proteins. To me, it feels almost like a circular argument: you're just confirming what's near the plasma membrane versus what's near the ER, without any clear specificity. (To put this another way: neither co-immunoprecipitation nor TurboID labelling demonstrate a physical interaction between proteins, despite this claim popping up repeatedly in the manuscript!)
The PDLP5 versus PDLP6 experiment is more telling, but then the decision to then focus on SUS6, which it seems only just barely made the authors' very generous significance thresholds, is tenuous at best. I'm more intrigued by the PIP1/PIP2 results, which seem more biologically significant.

In any case, that's what the authors have chosen to do, and I can go along with them (even if sfGFP isn't a true negative control). But the genetic experiments are, again, overinterpreted. I agree that it seems that CALS7 and SUS6 are required for callose synthesis in PDLP6 overexpression lines, which is a nice example of (at least partial) epistasis. But, this is basically saying that callose synthesis is required to synthesize callose-of course it is!-and the conclusion might be that CALS7 and SUS6 are each important in that genetic pathway or in this functional role without claiming that these are somehow working together in a physical complex, which really isn't demonstrated here. I think you could hint at that as one possible hypothesis in the discussion, but it seems like such an overreach. For example, if SUS6 needs PDLP6 to localize to PD, why does SUS6 enrich at PD in the epidermis when PDLP6 does not?

Reviewer #2 (Comments for the Author):

The manuscript "Plasmodesmata-located proteins regulate plasmodesmal function at specific cell interfaces in Arabidopsis" examines the consequences of the overexpression of two PLASMODESMATA-LOCATED PROTEIN (PDLP) family members, PDLP5 and PDLP6. They find that overexpression of either leads to increased callose deposition at plasmodesmata at specific cell-cell interfaces. They characterize the patterns of callose deposition and then demonstrate a link between PDLP6 and starch hyperaccumulation, a presumed result of its phloem localization. The distinct expression patterns and plasmodesmata effects of the two proteins are then used as the basis for determining whether the proteins have distinct molecular partners by proximity labeling. After identifying CALLOSE SYNTHASE 7 (CalS7) and SUCROSE SYNTHASE 6 (SUS6) they demonstrate a genetic interaction between these proteins and PDLP6, supporting the hypothesis that, together, these proteins may act at the phloem to regulate sugar flux.

The findings of the manuscript are interesting because they shed light on potential heterogeneities in plasmodesmata, an emerging topic in plant cell biology. The use of proximity labeling to identify protein partners is at the forefront of

modern techniques. The experiments are well designed and generally well performed with appropriate controls. The procedures and resources used are well described in the Materials and Methods section of the manuscript. The manuscript is relatively easy to read, although several ambiguities or misstatements are highlighted below. There are some conclusions drawn, however, that are not supported by the current analysis. Re-visiting these discrepancies may be useful to the authors:

1. Lines 374-375 and line 377: co-immunoprecipitation does not give evidence of physical interaction. Instead, it shows that the proteins can associate with each other in the plant cells, probably as part of a complex that includes at least the two proteins under investigation. Thus, the phrase "pulls down" in line 376 is also misleading. Along the same veins, the statements regarding the formation of a PDLP6-SUS6-CalS7 complex are not reflective of the actual experiments presented. The data presented in Fig. 6C show that the three proteins were not co-expressed at the time and the pull down of one led to the identification of the other two proteins. How do the authors know that there is one complex and not several others PDLP6-SUS6, CalS7 and PDLP6, or CalS7 and SUS that exist as separate entities in the same cells and locations? Direct interaction can be demonstrated by in vitro assays, heterologous assays or in planta FRET.
2. Lines 130-132 overstate the authors' findings. The overexpression of PDL5 orPDLP6 results in the hyperaccumulation of starch. The authors presume that this is due to the reduced intercellular movement of sugars since no experimental data about sugar movement is presented.
3. The authors refer to "distinct functional roles" of PDLPs throughout the text. A survey of the literature and the current work would argue, at least to this reviewer, that the role is the same: to regulate callose at PD. This is borne out by the proximity labeling results (Fig. 5E). The outcome of callose deposition varies, but the molecular function of the PDLPS seems to be the same, whether the function is in defense (PDLP5) or in sugar transport (PDLP6).
4. In the text, the authors describe differences in the amount of starch observed in Fig. 2A. However, the data presented is only qualitative and not quantitative. What were the frequency of these observations? Can the amount of starch be quantified for better, quantitative comparisons?
5. For Fig. 2D, does n refer to the number of cells? How many cells were seen in one section? In other words, it is not clear how much sampling was done or how many biological replicates were examined.

Minor comments.
1. Line 23: what is meant by 'membrane conduits'?
2. Line 28 states "...(PDLPs) regulate callose deposition at plasmodesmata through unknown mechanisms' is not wholly true. Quite a bit is known about how PDLP5 coordinates with CalS1 and S10 for callose deposition, and the role of SA in inducing callose deposition through PDLP5 activation. Indeed, the authors' findings about PDLP6 likely mediating callose deposition through CalS7 are in line with what is already known about PDLP5.
3. Lines 58-59 state "The tissues responsible for photosynthesis are known as sources". This is incorrect. Photosynthetic tissues are known as source when they are net exporters of photosynthate. Many other tissues perform photosynthesis but are net importers of photosynthate.
4. "It's" is used throughout the manuscript. Please replace with "It is".
5. Line 147 and line 153 have citations incorrectly formatted citations.
6. Line 325-325 needs a citation.
7. The Discussion is long and unfocused. It should be revised to be more focused and succinct. The section about PDLP5 and salicylic acid, for example, can be omitted.
8. The images presented in Figure 1C are too small. The border of the plate does not need to be seen. The smaller plants can be viewed at higher magnification if this is clearly shown by a different scale bar and stated in the figure legend.

Reviewer #3 (Comments for the Author):

The manuscript by Li et al explores the mechanism of Pd regulation by two Pd-localized proteins, PDLP5 and PDLP6. The authors demonstrate that although transgenic plants overexpressing these proteins accumulate callose, starch, have retarded growth and restricted Pd, their phenotypes have tissue-specific differences. Using proximity labeling in the overexpressing plants, they identified phloem-specific sucrose synthase (SUS6) as PDLP6 partner, suggesting a role of this PDLP in phloem-specific callose synthesis. They further provide evidence of genetic and in planta interaction between PDLP6, SUS6 and CALS7. Overall, the study presents a good experimental scope and data integrity to shed new light on the role of Pd and PDLPs in sugar transport. However, a major drawback of the study is that some of the essential genetic and biochemical evidence are still needed in order to prove the hypothesis that PDLP6 regulates callose synthesis in the phloem through the PDLP6-SUS6-CALS7 pathway. The additional evidence is required mainly because the current conclusions are based on the overexpression approach. It is known that overexpression of other Pd proteins can also lead to Pd restriction, callose synthesis, and starch accumulation.

Perhaps, in addition to the OE analysis, a more in-depth characterization of the pdlp6 mutant or generation of a phloem-specific pdlp mutant (i.e. pdlp3/6/8) would be useful.
Throughout the manuscript, the authors place too strong an emphasis on the "cell-type" specific functions of PDLP5 and PDLP6 while ignoring the fact that the two genes are also differentially regulated at the protein level. It is assumed that PDLPs are functionally redundant and that their role diverges primarily at the cell-specific expression levels. This is in contrast to the data they provide with the OE analysis.

Specific comments are listed below:

lines 140-141: how over-expression of the wt protein can avoid gene redundancy? The redundancy issue is relevant in a mutant analysis.

lines 221-225: in the EM images both in fig. 2C and S3C, it would be helpful to show even lower magnification and indicate the location of SE and CC in relation to the cells from which quantification was performed. From the current blow-ups, it is not clear how the BS and MC cells were determined as such. Also, please indicate how many independent sections and how many images have been used for quantification.

line 229: this looks more like an epistasis analysis, not synergistic.

Lines 239-240: a more comprehensive interpretation is needed here: disruption of Pd in the mesophyll cells (cher1-4) overrides the effect of Pd closure in the phloem tissue, which is consistent with the direction of sugar flow from mesophyll to phloem.

lines 243-244: this reasoning is hardly valid, because based on the OE analysis, one cannot conclude/predict that expression pattern is responsible for the observed differences. The conclusion from the OE analysis can only be that these PDLPs are regulated differently at the protein level (beyond their differential expression).

lines 262-265: the labeling of the cells in fig. 3C seems quite arbitrary, as it is hardly possible at this magnification/image quality to tell the different vasculature cell types apart, except perhaps the xylem cells. The listed parameters cannot be determined from the images. So, either show a larger magnification and high quality images, and/or co-localize with known fluorescent markers of the respective cell types. Generally, the labeling of cells should appear also on the DIC images for comparison. In one of the panels in fig. 3C the X label is off.

Lines 288-289: callose quantification is commonly done using a fixed tissue (see Lee et al 2011 https://pubmed.ncbi.nlm.nih.gov/21934146/). Live tissue staining may introduce bias of induced de novo callose synthesis which may not represent true resting levels of callose in those plants. Also, it would be better to use a different pseudo-color for callose signal throughout the paper to distinguish it from yfp/gfp.

Lines 289-294: in the statistical analysis of fig. 4 please include the comparison between pdlp6 and control.

Lines 294-297: In fig. 4C the DIC channel images are not shown. The intensity plots in the lower panels cannot stand for quantification, these are simply replications of what is shown in the images above and therefore redundant.

Lines 316-318: this reasoning is in contrast to the data. Based on the OE phenotypes, it is already evident that PDLP5 and PDLP6 are themselves regulated differentially, i.e. they are functionally non-redundant proteins. If only the cell-specific factors determine the divergence between PDLP5 and PDLP6, then the OE phenotypes of the two proteins should be the same, but this is not the case.

Lines 328-330: it is not clear why pUBQ10 was used instead of the p35S which was already used for the phenotype analysis. Does the pUBQ10-driven expression also lead to growth and starch phenotypes as the 35S? This info needs to be provided, especially since the results of the proximity labeling might be quite different depending on the strength of the exhibited phenotype of the plants.

Lines 336-337: please also include separate images for the callose signal in fig. 6A.

In fig. 6B please indicate what parameter has been measured (e.g. Pearson coeff. of co-localization etc.), rather than "percent of overlap" which is not a standardly used parameter for co-localization quantification. Also, it is not clear what does n represent in this graph?

Line 337: here and elsewhere, please avoid the term "physical interaction" as it implies direct interaction tested in vitro. Only in planta data is provided.

In fig. 6C it would be helpful to include PDLP5 as well in the co-IP analysis, as this would better demonstrate/confirm

whether there's divergence between the two PDLPs.

Lines 423-426: again, this explanation is missing out on the point that it is at the protein level that the two PDLPs diverge.

Line 436: As PDLPs are PM-associated, their cell-to-cell movement is quite unlikely.

Lines 444-453: the growth retardation, Pd callose and starch accumulation phenotypes are not limited to PDLP overexpression only. It has been reported for other Pd-proteins as well (see Zavaliev et al 2010, https://pubmed.ncbi.nlm.nih.gov/19887501/). The likely explanation of the OE phenotype is the Pd restriction that results either from overaccumulation of a Pd protein, or induction of callose as a secondary stress response to Pd malfunction, or both.

Lines 512-514: this statement is confusing. No SA levels have been correlated with starch accumulation either in PDLP5 OE or PDLP6 OE, so this discussion on the role of SA is too speculative.

Overview

We would like to express our sincere appreciation for your valuable feedback. Your insightful comments have greatly contributed to strengthening the manuscript. In response to all comments, we have made significant revisions. Here, we outlined a few major changes we made.

1. We carefully revised the introduction based on the reviewers' recommendations.
2. We adjusted the part that might be perceived as a potential overinterpretation. For example, we revised the title of the paper to “Plasmodesmata-located protein 6 regulates plasmodesmal function in Arabidopsis vasculature.”
3. We removed the characterization of *sweet11;12* and *cher1-4* mutants to emphasize the functional characterization of PDLP6.
4. We provided the quantification of starch and sugar in the transgenic plants using a biochemical approach (Figures 2B-2D).
5. Using new genetic materials, we provided evidence of the expression of PDLP6 in phloem parenchyma cells, companion cells, and sieve elements (Figure 3C-3G).
6. Using new genetic materials and a cell biology approach, we showed that overexpression of PDLP6 affects plasmodesmal function in Arabidopsis vasculature (Figure 5E-5F).
7. Using in vitro pull-down assay, we showed the direct physical interaction between PDLP5, SUS6, and CalS7.
8. We removed certain parts of the discussion following the recommendations.

Overall, we are confident that these revisions have significantly improved the quality of our manuscript. We are grateful for your time and attention to our work.

Reviewer #1:

In this manuscript, Li et al. share several exciting new discoveries about the enigmatic PDLP family and present a major methodological advance in how we can identify more proteins that localize to plasmodesmata using proximity labelling approaches. I think that many of the findings in this paper are impressive and will be of great interest to the PD field. My enthusiasm for the paper is lessened, unfortunately, for two main reasons. First, there are at least two distinct stories here, one about the phenotypes associated with PDLP6 and another about the TurboID method for identifying putative PDLP6 interactors, which can be connected but make this manuscript unwieldy, in my view. Second, and perhaps as a consequence of the first issue, the way this manuscript is written is often confusing if not outright misleading, often making claims that do not reflect the actual data as presented or that are not well-supported by the literature. Therefore, my recommendation is to substantially (substantially!!) rewrite the manuscript to narrowly focus on reporting the data presented here (not speculative ideas beyond the data presented here) clearly and coherently, and, in any case, to consider splitting the manuscript into two publications that will allow the experiments to be fully appreciated by readers.

We appreciate the reviewer's enthusiasm for the manuscript. In response to the reviewer's concern, we have revised the manuscript substantially, placing a stronger emphasis on elucidating the role of PDLP6 in regulating plasmodesmal function in Arabidopsis vasculature.

I could go line by line, but that would be overwhelming for a review. Instead, I will highlight a couple of major issues, and use a couple of smaller points as examples; but I urge the authors to carefully go through each section and consider whether the claims made are central to sharing their experimental conclusions.

For example...
59 - starch is a product of photosynthesis, but not always a major one, that depends a lot on conditions and photoperiod
61 - starch is hydrolyzed to maltose, which is further hydrolyzed to glucose, and then metabolized to sucrose-I don't know why these details are here anyway, you could just say that starch is metabolized to sucrose
62 - source-to-sink transport happens during the days and the nights in Arabidopsis, not only at night. Sinks include not only non-photosynthetic tissues but also actively growing tissues, including young leaves (see papers from the last thirty years on this topic, from https://doi.org/10.1105/tpc.9.8.1381 through https://www.pnas.org/doi/abs/10.1073/pnas.1919196117).

As suggested by the reviewer, we decided to focus on the role of PDLP6 in regulating plasmodesmal function in the vasculature. We removed most of the statements on the sugar movement from the introduction.

Throughout the starch section of the results-if source-to-sink trafficking is disrupted, starch will likely accumulate, but the inverse statement isn't true: if starch has overaccumulated, it could be for many reasons completely unrelated to source/sink trafficking. This needs to be clearly stated throughout. (See line 186 for just one of several examples where this is overstated.) There also isn't much discussion about other sinks for sugars, such as vacuoles.

We agree with the reviewer’s comments that starch overaccumulation could be attributed to other reasons, including defects of starch degradation, enhanced starch synthesis, and decreased sugar storage in the vacuole. However, overexpression of PDLPs is known to suppress

plasmodesmata-mediated trafficking. We thus hypothesized that PDLPs affect starch accumulation by affecting plasmodesmata-mediated sugar movement. However, we do not go into the argument about the two different routes for sugar movement in mature leaves with our current revision.

We have revised the related statements to incorporate the reviewers' comments.

"Our findings suggest that the overexpression of PDLP5 and PDLP6 compromises sugar transportation in mature leaves." (lines 162-163 in the revised manuscript)

TEM is not a more reliable method for quantifying starch levels than Lugol's stain. An improved method would be to determine starch concentrations using standard enzymatic approaches. The TEM results are easily skewed by other factors, like differences in starch granule morphology or chloroplast physiology. For instance, there might be more overall starch but spread across fewer granules, making visualization by TEM challenging.

As recommended by the reviewer, we removed the starch granule quantification of TEM images. We've included standard enzymatic approaches to quantify starch and Sugar (Figures 2B-2D).

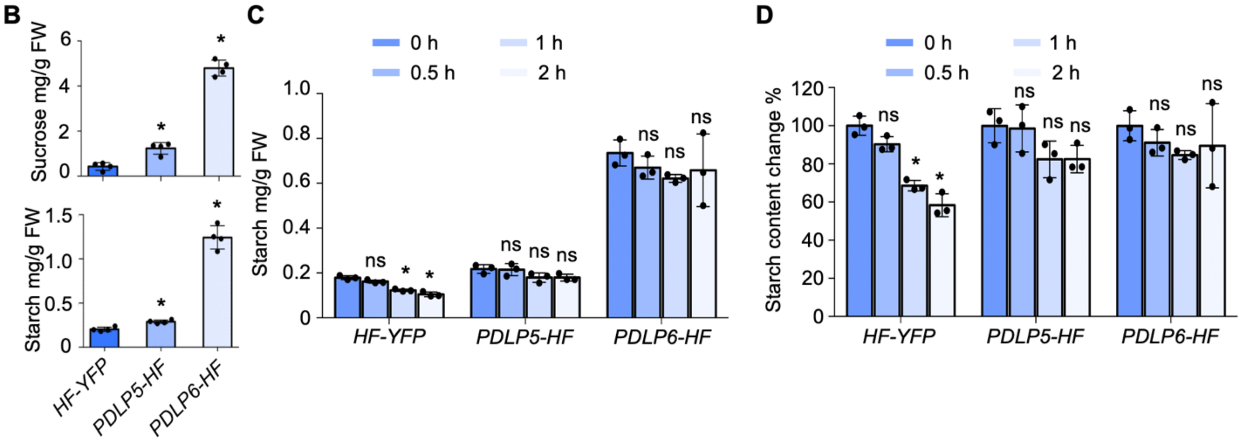

Figure 2B-2D. **Quantification of sucrose and starch. B**) Quantification of sucrose and starch contents in leaves of high-light treated plants at the end of the night. Mature leaves from three plants were combined to form a single replicate. The plots show the mean with SD (n = 4). Asterisks indicate statistically significant differences analyzed with a two-tailed *t* test (*, $P < 0.01$). **C**) Quantification of starch content in leaves of high-light treated plants at various time points. The '0 h' designation represents the end of the day; 0.5 h, 1 h, and 2 h indicate time points following the end of the day. Mature leaves from three plants were combined to form a single replicate. The plots show the mean with SD (n = 3). The values at 0.5 h, 1h, and 2 h time points were compared to that at 0 h for each genotype. Asterisks denote statistically significant differences analyzed with a two-tailed *t* test (*, $P < 0.01$). ns: no significance. **D**) Changes in starch content relative to the end of the day at different time points. The plots show the mean with SD (n = 3). The values at 0.5 h, 1h, and 2 h time points were compared to that at 0 h for each genotype. Asterisks denote statistically significant differences analyzed with a two-tailed *t* test (*, $P < 0.01$). ns: no significance.

The path of sugars: PD transport is extremely restricted in source leaves compared to sink leaves (see , which has often been interpreted as evidence that PD trafficking does not play a major role in apoplastic loading (at least, until you get into the phloem). suc2 mutants are nearly lethal with extreme growth phenotypes under standard growth conditions. As originally described in the 2012 paper, sweet11;12 mutants have almost no phenotype under normal growth conditions and weak phenotypes when plants are subjected to 450 uE light (~4x the amount of light used by most labs),

with the strongest phenotypes observed when plants are transferred from normal to extreme high-light environments. The suc2 data strongly support the apoplastic loading hypothesis. The sweet11;12 phenotype suggests that these facilitators at the PP - CC interface contribute to phloem loading and make important contributions under some physiological conditions, but do not support that this is the exclusive (or even primary) mechanism of phloem loading in Arabidopsis. Braun (2022) argues that this reflects some genetic redundancy, which might be true (maybe sweet13 is important, for instance), but it seems that an easier hypothesis is the one that was prevalent until the 2012 sweet paper: sugar concentrations are very high in plant apoplasts, and sugars are probably exported from cells other than the phloem parenchyma. My point with all of this commentary is that, in reality, none of this actually matters for the authors to present their data clearly. Instead of focusing on debates about the route taken by sugars, the authors could just focus on explaining their results. I'm not even sure, to be honest, that the sweet11;12 story needs to be here at all unless you split this into two papers.

As suggested by the reviewer, we decided not to put much weight on debates about the two different routes for sugar movement in mature leaves. We excluded the data related to *sweet11;12* and *cher1-4* mutants from the revised manuscript.

Following up on that point-why wasn't PDLP6-HF crossed to sweet11;12? Note that line 239 claims "synergistic" impacts, but "synergy" should be reserved for effects of mutant combinations that are beyond those that could be predicted from either mutant alone, and a quantitative increase in starch accumulation when you mix these all together isn't something unexpected. Also, if PDLP6-HF is already decreasing sugar loading, why does the cher1 mutant enhance that phenotype (why aren't they epistatic)? Obviously we can come up with explanations, I'm just trying to illustrate a situation where the results don't have an obvious conclusion to be drawn, but the authors have picked on interpretation over others without clear explanation.

We have crossed *PDLP6-HF* to *sweet11;12*. To follow the reviewer's previous suggestion, we decided not to include the data in this revision.

We agree with the reviewer that "synergistic" is not the ideal term to describe the results presented in our prior version. We will use the proper term when presenting the mutant phenotype in the future.

On the next section, Callose deposition: I don't think that the data strongly support the hypothesis that PDLP functions are determined by their cellular environment, as is repeatedly implied here and relies on the callose deposition data. One hypothesis to explain the results shown is that PDLP5 and PDLP6 control callose biosynthesis differently in different cell types. But, the authors already showed that the phenotypic effect of PDLP overexpression is remarkably sensitive to the degree of protein overexpression; therefore, another hypothesis is that the minor and inconsistent difference in callose accumulation between the PDLP5 and PDLP6 overexpression lines is, instead, about some subtle differences in the expression of these genes in those cell types in the different lines. Or this could indicate other differences in the functionality of PDLP5 and PDLP6. The callose deposition measured in roots doesn't seem to be localized to PD (at least to my eye), so I'm not sure exactly what we're looking at here.

1. Our findings support that PDLP6 functions in the vasculature with SUS6 and CalS7 to regulate callose biosynthesis. We agree with the reviewer that PDLP5 and PDLP6 control callose biosynthesis differently in different cell types. As overexpression of PDLP6 does not lead to callose accumulation between epidermal cells and mesophyll cells, it might not regulate callose accumulation in those cell types similarly to PDLP5. We hypothesize that PDLP5 regulates callose

deposition through a SUS-independent pathway. As discussed, PDLP5 might regulate callose synthesis through a cytosolic invertase (CINV)-dependent pathway (Lines 422-432 in the revised manuscript). However, we cannot completely rule out the possibility that subtle differences in the expression of PDLPs-HF in different cell types in the transgenic lines contribute to the variations in callose accumulation across cell types in PDLP5 and PDLP6 overexpression lines.

2. To visualize callose signals in Arabidopsis roots, we imaged them using a 20x objective (Figure 4C). Consequently, the resolution is insufficient to clearly observe plasmodesmal callose, especially in the vasculature. Images taken using 20x objective showing more typical PD callose puncta between epidermal cells and cortex cells in roots are provided below. Using a 63x objective, we could better observe plasmodesmal callose accumulation. We believe the signals detected in Figure 4C present callose signals at plasmodesmata.

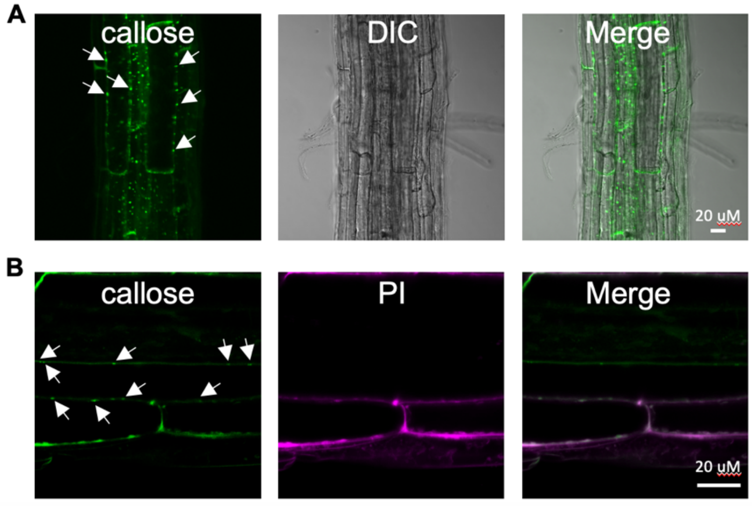

PD callose staining in roots. A) Callose signal in roots using a 20x objective. **B**) Callose signal in roots using a 63x objective. Green signals represent aniline blue stained-callose in roots. PI was used to stain cell walls. Merged images show the signals from callose and bright-field (DIC) (upper panel) or callose and PI (lower panel). Arrows indicate the puncta callose signals. PI: Propidium Iodide Scale bars = 20 μm.

Lastly, on the TurboID section: As is clearly shown in panel 5D, none of these proteins localize exclusively to PD, as expected. Instead, PDLP5 and PDLP6 are plasma-membrane proteins enriched at PD, and MCTP3 is an ER protein enriched at PD. This means that negative controls would be plasma membrane-localized proteins that are not enriched at PD (for PDLP5 and PDLP6) or ER proteins that are not enriched at PD (for MCTP3). Instead, MCTP3 is presented as a "control", which it is not-that's just a different experiment-and sfGFP is used as a "control" in immunoprecipitations, which it is not. Plasma membrane proteins tend to co-IP with each other, certainly more than they do with cytosolic proteins. It's no surprise that MCTP3-TbID labeled other MCTPs/synaptotagmins and Golgi-associated proteins, whereas PDLPs labelled plasma membrane proteins. To me, it feels almost like a circular argument: you're just confirming what's near the plasma membrane versus what's near the ER, without any clear specificity. (To put this another way: neither co-immunoprecipitation nor TurboID labelling demonstrate a physical interaction between proteins, despite this claim popping up repeatedly in the manuscript!) The PDLP5 versus PDLP6 experiment is more telling, but then the decision to then focus on SUS6, which it seems only just barely made the authors' very generous significance thresholds, is tenuous at best. I'm more intrigued by the PIP1/PIP2 results, which seem more biologically significant.

1. To address the reviewer’s concern about the proper control, we acknowledged that MCTP3 is not the best control for PDLP5 and PDLP6 in our proximity labeling. Instead of claiming MCTP3 as a control for the PDLPs, we rephrased the statement as follows:

"We also included multiple C2 domains and transmembrane protein 3 (MCTP3) as it is targeted to the ER membrane of PD (PD-ER; also known as desmotubule) (Brault et al., 2019), which is different from PDLP5 and PDLP6 that are localized to the plasma membrane within plasmodesmata (PD-PM; Figure 6A) (Thomas et al., 2008; Lee et al., 2011)." (lines 265-269 in the revised manuscript)

Nevertheless, we believe the identification of functional partners of MCTP3 allows us to demonstrate the power of enzyme-catalyzed proximity labeling in resolving protein complexes at different sub-domains within plasmodesmata or the ER membrane. The data set also allows us to narrow down candidates further to investigate functional partners of the PDLPs. More importantly, the data set could be informative to the PD community. We thus decided to include the data in the manuscript but move them into supplemental data. In Figure 6E, we only present the comparison between PDLP5 and PDLP6.

2. To address the reviewer's concern about our generous significance thresholds, we included the following explanation in this revision.

"We utilized MS2-based isobaric (TMT/iTRAQ) reporter ion quantification in our proximity labeling assay, which demonstrates high precision but exhibits ratio compression, leading to an underrepresentation of the actual level of enrichment (Wühr et al., 2012; Savitski et al., 2013). To address this, we set a statistical cutoff at log2FC > 0.2 or < -0.2. Additionally, we employed multiple testing corrections and utilized *q*-values for a rigorous statistical enrichment determination. Notably, histograms revealed a relatively flat distribution of *q*-values beyond 0.1 (Supplemental Figure 7), suggesting that protein hits with *q*-values below 0.1 are unlikely to be random targets. Consequently, we established a *q*-value cutoff of 0.1 as the threshold for defining statistical significance." (lines 283-291 in the revised manuscript)

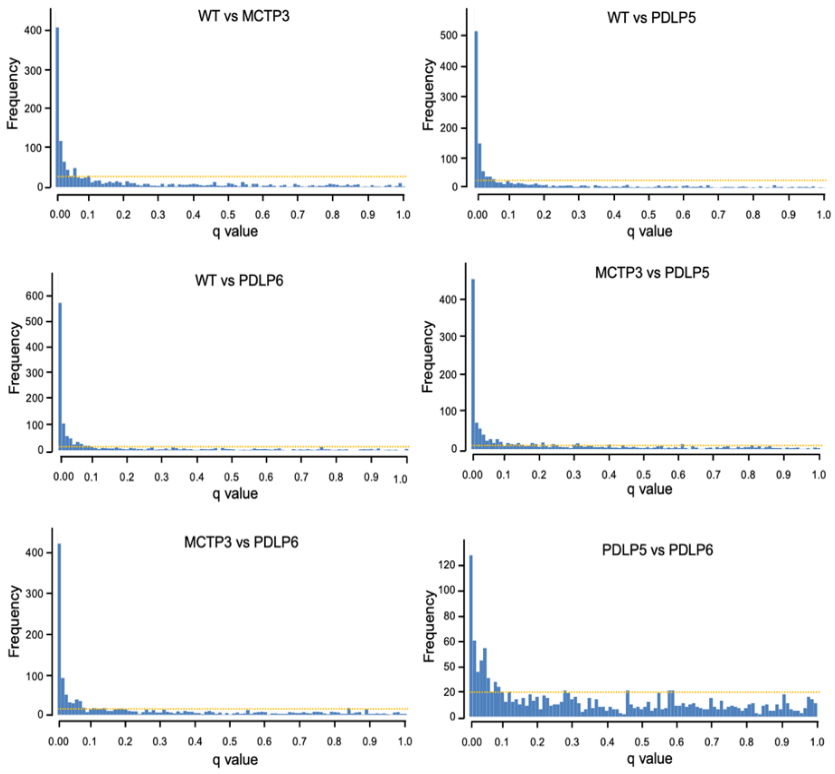

Supplemental Figure 7. Histograms of *q* values. A two-sample *t*-test assessed differential expression. Benjamini-Hochberg (BH) *p*-value adjustment was used for multiple test corrections. The x-axis represents different bins or range groups of *q* value (0-0.01, 0.01-0.02…0.99-1.0), while the y-axis represents the number of hits within each bin. The dotted line indicates the relatively flat distribution of *q* values beyond 0.1. These histograms served as a statistical tool to assess enrichment without bias towards specific protein candidates. Notably, we observed a relatively flat distribution of q values beyond 0.1, indicating that protein hits with *q* values below 0.1 are unlikely to be random targets. Therefore, we determined a *q* value cutoff of 0.1 as the threshold for defining statistical significance.

More importantly, we demonstrated the partial PD association of SUS6 and the physical and genetic interactions between PDLP6 and SUS6. We provided new evidence from an in vitro pull-down assay showing that PDLP6 physically interacts with SUS6 and CalS7 (Figure 7E).

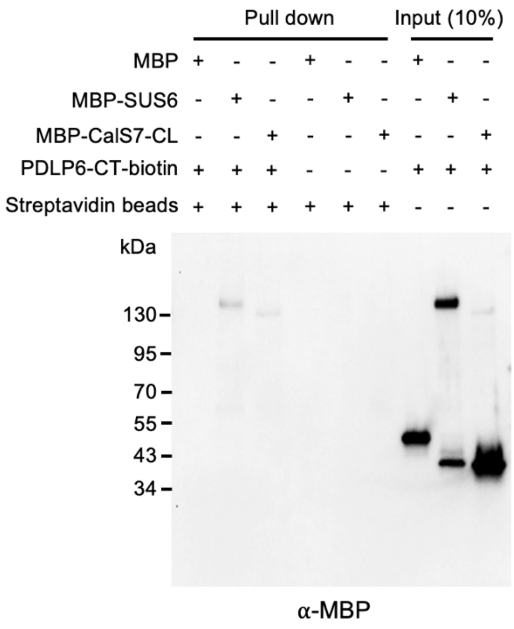

Figure 7E. In vitro pull-down assay. Direct interaction between PDLP6 and SUS6, and PDLP6 and CalS7. The biotinylated C-terminal tail of PDLP6 (PDLP6-CT-biotin) was incubated with recombinant proteins, MBP, MBP-SUS6, or MBP-CalS7. Magnetic beads coupled with streptavidin were used to pull down PDLP6-CT-biotin and the interacting proteins. A MBP antibody was used to detect the interaction between PDLP6 and MBP fusion proteins.

3. We are very much intrigued by the PIP1/PIP2 results as well. Since we decided to focus on PDLP6, we didn't include the work on the PIPs in this revision.

In any case, that's what the authors have chosen to do, and I can go along with them (even if sfGFP isn't a true negative control). But the genetic experiments are, again, overinterpreted. I agree that it seems that CALS7 and SUS6 are required for callose synthesis in PDLP6 overexpression lines, which is a nice example of (at least partial) epistasis. But, this is basically saying that callose synthesis is required to synthesize callose-of course it is!-and the conclusion might be that CALS7 and SUS6 are each important in that genetic pathway or in this functional role without claiming that these are somehow working together in a physical complex, which really isn't demonstrated here. I think you could hint at that as one possible hypothesis in the discussion, but it seems like such an overreach. For example, if SUS6 needs PDLP6 to localize to PD, why does SUS6 enrich at PD in the epidermis when PDLP6 does not?

1. As stated above, we demonstrated the physical interaction between PDLP6, SUS6, and CalS7 using an in vitro pull-down assay (Figure 7E). However, we did not show the presence of PDLP6-SUS6-CalS7 complex. We thus revised our statement through the manuscript. For example, we revised one of the claims as follows:

"We propose that PDLP6 functions together with SUS6 and CalS7 to regulate callose biosynthesis in the vasculature, regulating plasmodesmal function." (lines 377-378 in the revised manuscript)

2. We detected the PD localization of SUS6-sfGFP in the epidermis of *N. benthamiana*. When overexpressed in plants, SUS6 and PDLP6 are detected in most cell types, including epidermal cells.

Reviewer #2:

The manuscript "Plasmodesmata-located proteins regulate plasmodesmal function at specific cell interfaces in Arabidopsis" examines the consequences of the overexpression of two PLASMODESMATA-LOCATED PROTEIN (PDLP) family members, PDLP5 and PDLP6. They find that overexpression of either leads to increased callose deposition at plasmodesmata at specific cell-cell interfaces. They characterize the patterns of callose deposition and then demonstrate a link between PDLP6 and starch hyperaccumulation, a presumed result of its phloem localization. The distinct expression patterns and plasmodesmata effects of the two proteins are then used as the basis for determining whether the proteins have distinct molecular partners by proximity labeling. After identifying CALLOSE SYNTHASE 7 (CalS7) and SUCROSE SYNTHASE 6 (SUS6) they demonstrate a genetic interaction between these proteins and PDLP6, supporting the hypothesis that, together, these proteins may act at the phloem to regulate sugar flux.

The findings of the manuscript are interesting because they shed light on potential heterogeneities in plasmodesmata, an emerging topic in plant cell biology. The use of proximity labeling to identify protein partners is at the forefront of modern techniques. The experiments are well designed and generally well performed with appropriate controls. The procedures and resources used are well described in the Materials and Methods section of the manuscript. The manuscript is relatively easy to read, although several ambiguities or misstatements are highlighted below. There are some conclusions drawn, however, that are not supported by the current analysis. Re-visiting these discrepancies may be useful to the authors:

We sincerely thank the reviewer for their interest and positive comments on our manuscript. In this revised version, we have presented additional evidence to substantiate our conclusions. The reviewer's comments have been thoroughly addressed, and the specific responses are outlined below.

1. Lines 374-375 and line 377: co-immunoprecipitation does not give evidence of physical interaction. Instead, it shows that the proteins can associate with each other in the plant cells, probably as part of a complex that includes at least the two proteins under investigation. Thus, the phrase "pulls down" in line 376 is also misleading. Along the same veins, the statements regarding the formation of a PDLP6-SUS6-CalS7 complex are not reflective of the actual experiments presented. The data presented in Fig. 6C show that the three proteins were not co-expressed at the time and the pull down of one led to the identification of the other two proteins. How do the authors know that there is one complex and not several others PDLP6-SUS6, CalS7 and PDLP6, or CalS7 and SUS that exist as separate entities in the same cells and locations? Direct interaction can be demonstrated by in vitro assays, heterologous assays or in planta FRET.

1. We corrected the use of “pulls down” We revised the statement as follows:

“PDLP6-YFP specifically enriched SUS6-HF.” (line 324 in the revised manuscript)

2. To better reflect our findings regarding the relationship between PDLP6, SUS6, and CalS7, we revised the statement as follows:

“We propose that PDLP6 functions with SUS6 and CalS7 in the vasculature to regulate plasmodesmal function.” (lines 38-39 in the revised manuscript)

“We propose that PDLP6 functions together with SUS6 and CalS7 to regulate callose biosynthesis in the vasculature, regulating plasmodesmal function.” (lines 377-378 in the revised manuscript)

3. We validated the directed physical interaction between PDLP6, SUS6, and CalS7 using an in vitro assay as suggested. Figure 7E shows that the biotinylated C-terminal tail of PDLP6, facing the cytoplasmic sleeve, pulled down maltose binding protein-SUS6 (MBP-SUS6) fusion proteins and MBP-CalS7-cytoplasmic loop (MBP-CalS7-CL) fusion proteins. These results confirm the direct physical interactions between PDLP6 and SUS6, as well as PDLP6 and CalS7.

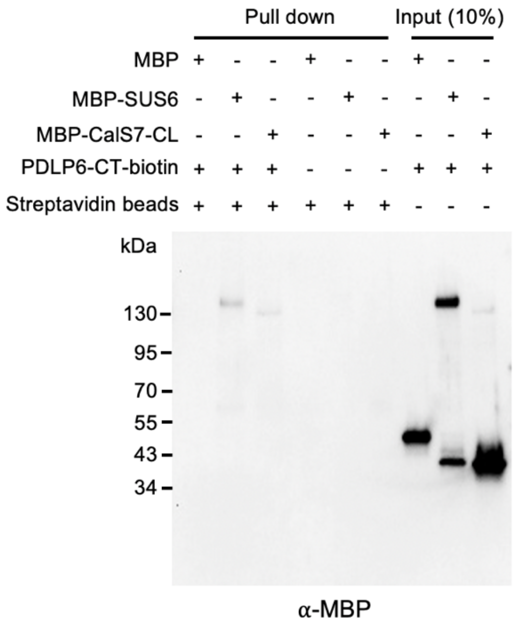

Figure 7E. In vitro pull-down assay. Direct interaction between PDLP6 and SUS6, and PDLP6 and CalS7. The biotinylated C-terminal tail of PDLP6 (PDLP6-CT-biotin) was incubated with recombinant proteins, MBP, MBP-SUS6, or MBP-CalS7. Magnetic beads coupled with streptavidin were used to pull down PDLP6-CT-biotin and the interacting proteins. A MBP antibody was used to detect the interaction between PDLP6 and MBP fusion proteins.

2. Lines 130-132 overstate the authors' findings. The overexpression of PDL5 or PDLP6 results in the hyperaccumulation of starch. The authors presume that this is due to the reduced intercellular movement of sugars since no experimental data about sugar movement is presented.

We acknowledge the reviewer’s concern about overstating the findings. Given that the direct measurement of the plasmodesmata-mediated sugar movement is technically challenging, we focused on the function of PDLP6 in regulating the plasmodesmal function in the vasculature. We generated Arabidopsis transgenic plants carrying *ProPDLP6:1xYFP* construct in wild-type Col-0 and *Pro35S:PDLP6-HF* (*PDLP6-HF*) backgrounds to express 1xYFP specifically in phloem. As shown in Figures 5E and 5F, YFP levels in non-vascular cells were significantly higher in Col-0 than *PDLP6-HF*, suggesting that *PDLP6* overexpression inhibits the plasmodesmata-dependent movement of molecules in the vasculature. In this revision, we emphasized the role of PDLP6 in regulating plasmodesmal function in Arabidopsis vasculature instead of presuming its function in the plasmodesmata-dependent sugar movement.

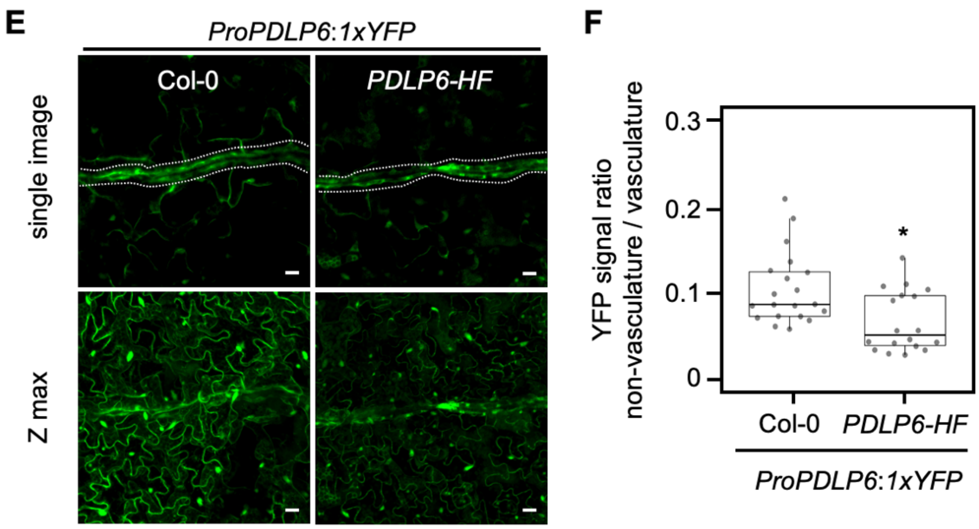

Figure 5E and 5F. The diffusion of YFP. E) The diffusion of YFP from the vasculature to mesophyll and pavement cells in leaves of Col-0 and *PDLP6-HF* expressing *ProPDLP6*:*1xYFP*. Signals were detected in cotyledons of 14-day-old seedlings. Scale bar = 20 µm. **F**) Quantification of the YFP signals diffused from the vasculature to the other cell types. Mean signal intensity was determined in the vasculature (dotted area) and non-vasculature near the leaf tip. The ratio between non-vasculature and vasculature was calculated. A cotyledon from each seedling was used for imaging. Col-0, n = 20; *PDLP6-HF*, n = 18. The asterisk indicates statistically significant differences analyzed with a Mann-Whitney *U* Test (*, $P < 0.05$).

3. The authors refer to "distinct functional roles" of PDLPs throughout the text. A survey of the literature and the current work would argue, at least to this reviewer, that the role is the same: to regulate callose at PD. This is borne out by the proximity labeling results (Fig. 5E). The outcome of callose deposition varies, but the molecular function of the PDLPS seems to be the same, whether the function is in defense (PDLP5) or in sugar transport (PDLP6).

We agree with the reviewer's viewpoint on the function of different PDLPs in positively regulating plasmodesmal callose deposition. When we discuss the cell-type-specific functions of PDLP5 and PDLP6, we mean that the PDLPs express in specific cell types and function together with cell-type-specific partners to regulate plasmodesmal callose accumulation. For example, PDLP6 expresses specifically in phloem and functions together with SUS6 and CalS7, which are specifically expressed in sieve elements, to regulate plasmodesmal callose accumulation. We revised the phrase “distinct functional roles” with a more appropriate term throughout the revised manuscript. For example:

“The findings also show that the impact of overexpressing *PDLP5* and *PDLP6* is most pronounced in the cell types where they are naturally expressed.” (lines 255-256 in the revised manuscript)

“Our findings suggest that SUS6 and CalS7 function with PDLP6, regulating callose accumulation in a specific cell type, likely in sieve elements.” (lines 359-360 in the revised manuscript)

4. In the text, the authors describe differences in the amount of starch observed in Fig. 2A. However, the data presented is only qualitative and not quantitative. What were the frequency of these observations? Can the amount of starch be quantified for better, quantitative comparisons?

The starch accumulation phenotype in the transgenic lines is highly reproducible. With the same growth conditions, we always observed the hyperaccumulation of starch, especially in *PDLP6-HF*.

We used standard enzymatic approaches to quantify the starch and sucrose concentration in the transgenic plants (Figure 2B-2D). The method provides a better quantitative comparison. In addition, we showed the changes in starch content at various time points after the end of the day. Figures 2C and 2D show that *PDLP5-HF* and *PDLP6-HF* exhibit a lesser reduction in starch content during the dark period compared to *HF-YFP*.

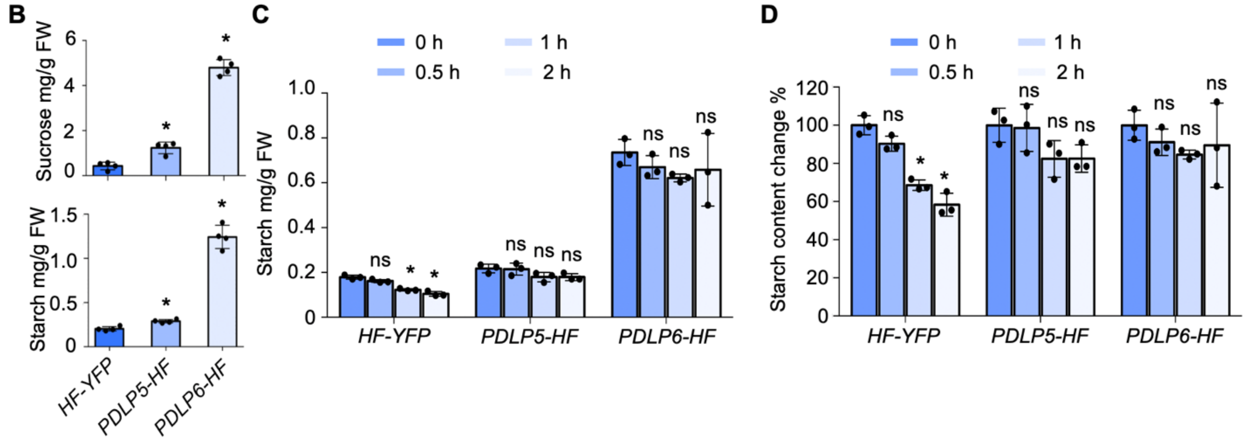

Figure 2B- 2D. **Quantification of sucrose and starch. B**) Quantification of sucrose and starch contents in leaves of high-light treated plants at the end of the night. Mature leaves from three plants were combined to form a single replicate. The plots show the mean with SD (n = 4). Asterisks indicate statistically significant differences analyzed with a two-tailed *t* test (*, $P < 0.01$). **C**) Quantification of starch content in leaves of high-light treated plants at various time points. The '0 h' designation represents the end of the day; 0.5 h, 1 h, and 2 h indicate time points following the end of the day. Mature leaves from three plants were combined to form a single replicate. The plots show the mean with SD (n = 3). The values at 0.5 h, 1h, and 2 h time points were compared to that at 0 h for each genotype. Asterisks denote statistically significant differences analyzed with a two-tailed *t* test (*, $P < 0.01$). ns: no significance. **D**) Changes in starch content relative to the end of the day at different time points. The plots show the mean with SD (n = 3). The values at 0.5 h, 1h, and 2 h time points were compared to that at 0 h for each genotype. Asterisks denote statistically significant differences analyzed with a two-tailed *t* test (*, $P < 0.01$). ns: no significance.

5. For Fig. 2D, does n refer to the number of cells? How many cells were seen in one section? In other words, it is not clear how much sampling was done or how many biological replicates were examined.

As suggested by Reviewer #1, we removed the quantitative data from the revision as TEM quantification of samples without serial blocks could introduce biases. Instead, we showed the quantification of sucrose and starch using standard enzymatic approaches (Figure 2B-2D). The method provides a better quantitative comparison. In addition, we showed the changes in starch content at various time points after the end of the day. Figures 2C and 2D show that *PDLP5-HF* and *PDLP6-HF* exhibit a lesser reduction in starch content during the dark period compared to HF-YFP.

In original Fig. 2D, “n” refers to the number of chloroplasts. The numbers of images and cells are listed below: Mesophyll cell: Col-0: 9 and 13; *cher1-4*: 9 and 12; *sweet11;12*: 8 and 12; *HF-YFP*: 8 and 9; *PDLP5-HF*: 8 and 30; *PDLP6-HF*: 8 and 24. BS: Col-0: 6 and 10; *cher1-4*: 7 and 13;

sweet11;12: 6 and 12; *HF-YFP*: 7 and 7; *PDLP5-HF*: 6 and 7; *PDLP6-HF*: 5 and 6. We processed four grids from two leaf disks for each genotype.

Minor comments.

1. Line 23: what is meant by 'membrane conduits'?

We avoid using membrane channels to prevent potential confusion with ion channels. "Conduits" might be more suitable than "membrane conduits." We have rephrased the statement as follows.

"The regulation of plasmodesmal aperture is considered one of the major mechanisms to modulate plasmodesmal function." (lines 23-24 in the revised manuscript)

2. Line 28 states "...(PDLPs) regulate callose deposition at plasmodesmata through unknown mechanisms' is not wholly true. Quite a bit is known about how PDLP5 coordinates with CalS1 and S10 for callose deposition, and the role of SA in inducing callose deposition through PDLP5 activation. Indeed, the authors' findings about PDLP6 likely mediating callose deposition through CalS7 are in line with what is already known about PDLP5.

We have rephrased the statement as follows.

"Among them, plasmodesmata-located proteins (PDLPs) promote callose deposition at plasmodesmata. In this study, we explored the function of PDLP5 and PDLP6 in different cell types. We further demonstrated the molecular mechanism underlying PDLP6 in regulating plasmodesmal function in the vasculature." (lines 26-29 in the revised manuscript)

3. Lines 58-59 state "The tissues responsible for photosynthesis are known as sources". This is incorrect. Photosynthetic tissues are known as source when they are net exporters of photosynthate. Many other tissues perform photosynthesis but are net importers of photosynthate.

We removed the introduction about the sugar movement as we decided to focus on the functional characterization of PDLP6 in regulating plasmodesmal function in the vasculature.

4. "It's" is used throughout the manuscript. Please replace with "It is".

We have revised it through the revised manuscript.

5. Line 147 and line 153 have citations incorrectly formatted citations.

We have corrected the citation.

"A similar trend was also observed for *PDLP5-HF* (Supplemental Figure 1A and 1B, Lee et al., 2011)." (lines 112-113 in the revised manuscript)

"The observed growth phenotypes are similar to Arabidopsis mutants *suc2* and *sweet11;12*, which are compromised in sugar transport from mature leaves to sink tissues, including young leaves, roots, flowers, and seeds. In addition to the growth phenotype, these mutants overaccumulate starch in mature leaves (Gottwald et al., 2000; Srivastava et al., 2008; Chen et al., 2012; Wippel and Sauer, 2012). We thus determined starch content in the transgenic plants using a Lugol's iodine staining method (Tran et al., 2019)." (lines 115-120 in the revised manuscript)

6. Line 325-325 needs a citation.

We have revised the statement and cited the reference.

“We also included multiple C2 domains and transmembrane protein 3 (MCTP3) as it is targeted to the ER membrane of PD (PD-ER; also known as desmotubule) (Brault et al., 2019), which is different from PDLP5 and PDLP6 that are localized to the plasma membrane within plasmodesmata (PD-PM; Figure 6A) (Thomas et al., 2008; Lee et al., 2011).” (lines 265-269 in the revised manuscript)

7. The Discussion is long and unfocused. It should be revised to be more focused and succinct. The section about PDLP5 and salicylic acid, for example, can be omitted.

We have removed the discussion on PDLP5 and salicylic acid in the revised manuscript. As. recommended by one of the reviewers for conciseness and to emphasize PDLPs' role in regulating plasmodesmal function, we excluded the starch overaccumulation phenotypes of *sweet11;12* and *cher1-4*. Consequently, the discussion on sugar movement paths has been removed. Additionally, information on the cell type specificities of other PDLPs in roots and the associated discussion has been eliminated. Meanwhile, we have rephrased certain statements in the Discussion.

8. The images presented in Figure 1C are too small. The border of the plate does not need to be seen. The smaller plants can be viewed at higher magnification if this is clearly shown by a different scale bar and stated in the figure legend.

We have cropped the images and enlarged the smaller plants in our revised manuscript (Figure 1C).

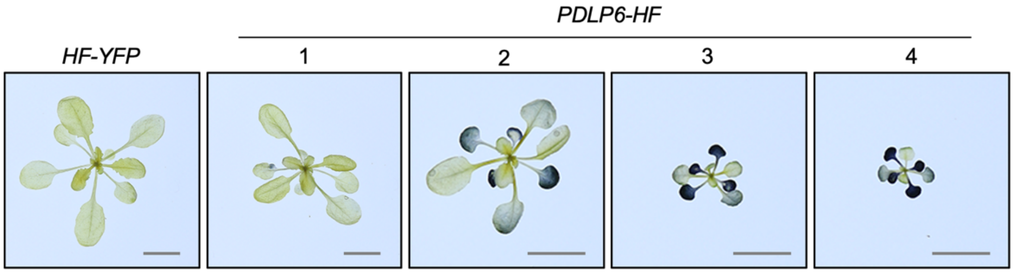

Figure 1C. Starch accumulation in *PDLP6-HF* transgenic lines. Plants were harvested at the end of the night and stained using Lugol’s iodine solution. Scale bars = 1 cm.

Reviewer #3:

The manuscript by Li et al explores the mechanism of Pd regulation by two Pd-localized proteins, PDLP5 and PDLP6. The authors demonstrate that although transgenic plants overexpressing these proteins accumulate callose, starch, have retarded growth and restricted Pd, their phenotypes have tissue-specific differences. Using proximity labeling in the overexpressing plants, they identified phloem-specific sucrose synthase (SUS6) as PDLP6 partner, suggesting a role of this PDLP in phloem-specific callose synthesis. They further provide evidence of genetic and in planta interaction between PDLP6, SUS6 and CALS7. Overall, the study presents a good experimental scope and data integrity to shed new light on the role of Pd and PDLPs in sugar transport. However, a major drawback of the study is that some of the essential genetic and biochemical evidence are still needed in order to prove the hypothesis that PDLP6 regulates callose synthesis in the phloem through the PDLP6-SUS6-CALS7 pathway. The additional evidence is required mainly because the current conclusions are based on the overexpression approach. It is known that overexpression of other Pd proteins can also lead to Pd restriction, callose synthesis, and starch accumulation. Perhaps, in addition to the OE analysis, a more in-depth characterization of the pdlp6 mutant or generation of a phloem-specific pdlp mutant (i.e. pdlp3/6/8) would be useful.

We appreciate the positive comments from the reviewer regarding our manuscript. In this revised version, we have included additional evidence to support our conclusions. While we acknowledge the reviewer's concern about relying on the overexpression approach for several key conclusions, our results demonstrate that this strategy effectively reveals the cell type-specific function of PDLP6. PDLP6 functions with cell type-specific partners, SUS6 and CalS7, to regulate plasmodesmal function in Arabidopsis vasculature. Upon reflection, we consider ourselves fortunate to have initiated our study by characterizing the overexpressors rather than the knockout mutants, as this approach led to the identification of the role of PDLP6 role in the vasculature.

We made diligent efforts to characterize the *pdlp6* mutant. Unfortunately, we did not observe any noticeable starch (Supplemental Figure 2D) or callose accumulation phenotypes (Supplemental Figure 2G) for the mutant, except for a slight decrease in starch grains observed through transmission electron microscopy (Supplemental Figure 2E). Given the potential for functional redundancy, we believe that characterizing *pdlp3/6/8* will likely provide more insights into the role of PDLPs in regulating plasmodesmal function in the vasculature. While our primary focus is on characterizing PDLP6, we plan to generate and analyze higher-order mutants in the future. Our responses to the reviewer's comments are provided below:

Throughout the manuscript, the authors place too strong an emphasis on the "cell-type" specific functions of PDLP5 and PDLP6 while ignoring the fact that the two genes are also differentially regulated at the protein level. It is assumed that PDLPs are functionally redundant and that their role diverges primarily at the cell-specific expression levels. This is in contrast to the data they provide with the OE analysis.

Bacterial infection has been shown to upregulate the expression of PDLP5 transcript and protein (Lee et al., 2011, https://doi.org/10.1105/tpc.111.087742); however, it remains unknown whether PDLP6 is regulated at the protein level.

We want to clarify that our findings do not lead to the conclusion that "PDLPs are functionally redundant and that their role diverges primarily at the cell-specific expression levels." If the divergence in the role of PDLPs were primarily at the cell-specific expression levels, we would anticipate observing plasmodesmal callose accumulation in all cell-cell interfaces in transgenic

plants overexpressing PDLP6 (*PDLP6-HF*), as the protein is expected to be highly expressed in all cell types, as suggested in Supplemental Figure 4. Our results indicate that PDLP6 is expressed in the vasculature and functions with specific partners, SUS6 and CalS7, to regulate plasmodesmal function. While cell type-specific expression of PDLP6 is a contributing factor, its function also relies on SUS6 and CalS7 to exert its cell type-specific effects. This conclusion is supported by our observation that overexpression of PDLP6 in *sus6* or *cals7* mutant backgrounds does not result in stunted plant growth and starch overaccumulation phenotypes (Figures 7F and 7G). Collectively, the data presented through the OE analysis support our main claim that PDLP6 functions with SUS6 and CalS7 to regulate plasmodesmal function in the vasculature.

Specific comments are listed below:

lines 140-141: how over-expression of the wt protein can avoid gene redundancy? The redundancy issue is relevant in a mutant analysis.

We recognize that the message may not have been articulated clearly. We intended to convey that in the case of gene redundancy, a lack of observable phenotypes in knockout mutants might be expected, as is likely the situation in this instance. Conversely, overexpressing a gene of interest can potentially reveal its function, as is demonstrated in this study.

To avoid confusion, we have rephrased the statement as follows:

"To determine the function of PDLPs, we individually overexpressed all eight members of PDLP (PDLP1-8) in Arabidopsis wild-type Col-0 using a 35S promoter (*Pro35S*). The PDLPs were fused with His and Flag (HF) tags." (lines 104-106 in the revised manuscript)

lines 221-225: in the EM images both in fig. 2C and S3C, it would be helpful to show even lower magnification and indicate the location of SE and CC in relation to the cells from which quantification was performed. From the current blow-ups, it is not clear how the BS and MC cells were determined as such. Also, please indicate how many independent sections and how many images have been used for quantification.

Images with lower magnification are provided. PP, CC, and SE are marked (Supplemental Figure 3). As suggested by Reviewer #1, we removed the quantitative data from the revision as TEM quantification of samples without serial blocks could introduce biases. Instead, we showed the quantification of sucrose and starch using standard enzymatic approaches (Figure 2B-2D). The method provides a better quantitative comparison. In addition, we showed the changes in starch content at various time points after the end of the day. Figures 2C and 2D show that *PDLP5-HF* and *PDLP6-HF* exhibit a lesser reduction in starch content during the dark period compared to *HF-YFP*.

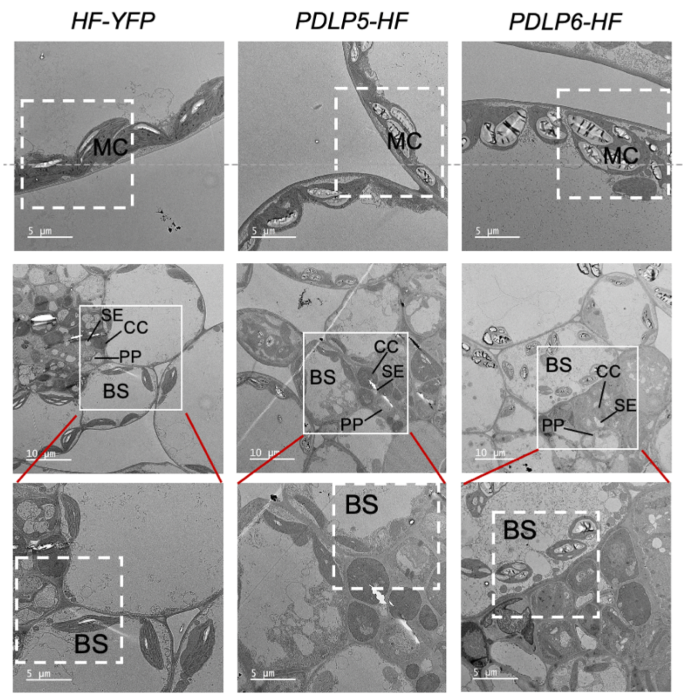

Supplemental Figure 3B. TEM images show the starch granules in chloroplasts in different cells of mature leaves. White dashed frames indicate the images in **Figure 2F**. MC: mesophyll cell; BS: bundle sheath cell; PP: phloem parenchyma cells; CC: companion cells; and SE: sieve elements.

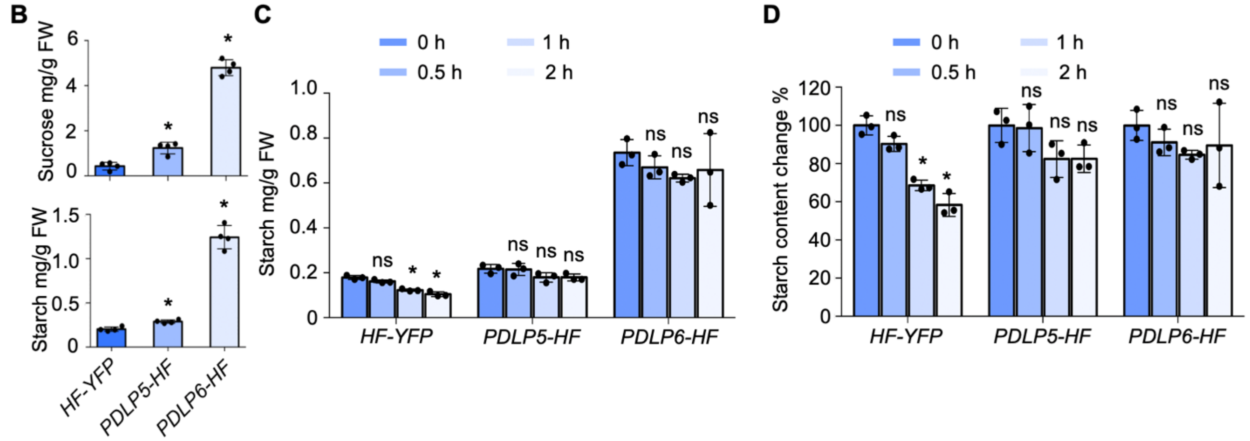

Figure 2B-2D. **Quantification of sucrose and starch. B**) Quantification of sucrose and starch contents in leaves of high-light treated plants at the end of the night. Mature leaves from three plants were combined to form a single replicate. The plots show the mean with SD (n = 4). Asterisks indicate statistically significant differences analyzed with a two-tailed *t* test (*, $P < 0.01$). **C**) Quantification of starch content in leaves of high-light treated plants at various time points. The '0 h' designation represents the end of the day; 0.5 h, 1 h, and 2 h indicate time points following the end of the day. Mature leaves from three plants were combined to form a single replicate. The plots show the mean with SD (n = 3). The values at 0.5 h, 1h, and 2 h time points were compared to that at 0 h for each genotype. Asterisks denote statistically significant differences analyzed with a two-tailed *t* test (*, $P < 0.01$). ns: no significance. **D**) Changes in starch content relative to the end of the day at different time points. The plots show the mean with SD (n = 3). The values at 0.5 h, 1h, and 2 h time points were compared to that at 0 h for each genotype. Asterisks denote statistically significant differences analyzed with a two-tailed *t* test (*, $P < 0.01$). ns: no significance.

line 229: this looks more like an epistasis analysis, not synergistic.

We removed the findings on *cher1-4* and *sweet11;12* as suggested by Reviewer #1.

We agree with the reviewer that “synergistic” is not an ideal term to describe the results presented in our prior version. We will use the proper term when presenting the mutant phenotype in the future.

Lines 239-240: a more comprehensive interpretation is needed here: disruption of Pd in the mesophyll cells (cher1-4) overrides the effect of Pd closure in the phloem tissue, which is consistent with the direction of sugar flow from mesophyll to phloem.

We removed the findings on *cher1-4* and *sweet11;12* as suggested by Reviewer #1.

lines 243-244: this reasoning is hardly valid, because based on the OE analysis, one cannot conclude/predict that expression pattern is responsible for the observed differences. The conclusion from the OE analysis can only be that these PDLPs are regulated differently at the protein level (beyond their differential expression).

The distinct starch accumulation patterns observed in the two PDLP overexpressors prompted our exploration into the cell type-specific expression of PDLP5 and PDLP6. Upon reflection, we recognize that this assumption was bold. To prevent possible confusion, we have revised the statement as follows:

“Single-cell RNA-sequencing analysis revealed that *PDLP* transcripts showed distinct expression patterns in different cells in the Arabidopsis leaf. Especially, *PDLP6* was detected in phloem parenchyma cells (Kim et al., 2021). To determine the cell type-specific expression of PDLP5 and PDLP6, we constructed *ProPDLP5:PDLP5-YFP* and *ProPDLP6:PDLP6-YFP*.” (lines 183-186 in the revised manuscript)

lines 262-265: the labeling of the cells in fig. 3C seems quite arbitrary, as it is hardly possible at this magnification/image quality to tell the different vasculature cell types apart, except perhaps the xylem cells. The listed parameters cannot be determined from the images. So, either show a larger magnification and high quality images, and/or co-localize with known fluorescent markers of the respective cell types. Generally, the labeling of cells should appear also on the DIC images for comparison. In one of the panels in fig. 3C the X label is off.

We provided new data to confirm the expression of PDLP6 in Arabidopsis phloem. We used a ClearSee method to visualize PDLP6-YFP in the vasculature (Figure 3C). We also detected the expression of PDLP6-YFP in different phloem cell types (Figure 3D). In addition, we observed the colocalization of PDLP6-YFP signals with various cell type markers (Figure 3E-3G). Phloem parenchyma cells, companion cells, and sieve elements were marked using *ProSWEET13:SWEET13-mCherry*, *ProSUC2:PP2A1-mCherry*, and *ProSEOR2:SEOR2-mCherry*, respectively.

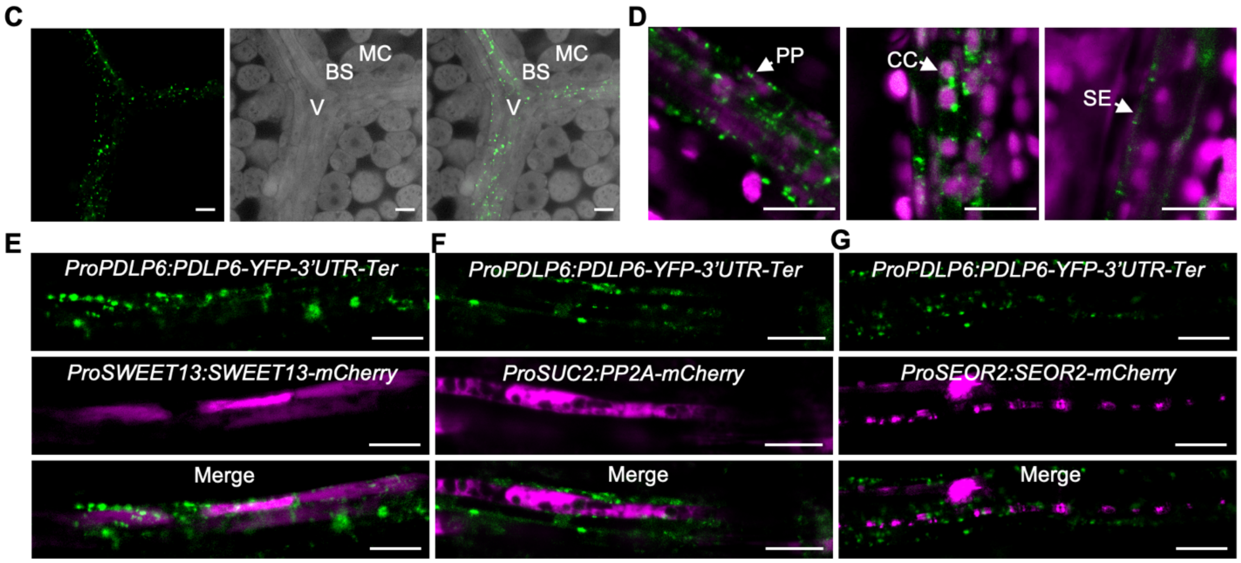

Figure 3C-3G. PDLP6 subcellular localization. C) PDLP6-YFP proteins were observed in the vasculature. The leaf sample was cleared by ClearSee solution. MC: mesophyll cell; BS: bundle sheath cell; V: vasculature. Scale bars = 10 µm. **D**) PDLP6-YFP proteins were detected in phloem parenchyma cells (PP), companion cells (CC), and sieve elements (SE). Cell types were determined based on their sizes, chloroplast arrangement, and cell wall ingrowth phenotype as previously described (Cayla et al., 2015). Chlorophyll autofluorescence was shown in magenta. Scale bar = 10 µm. **E-G**) The colocalization of PDLP-YFP signals with various cell type markers. **E**) *ProSWEET13:SWEET13-mCherry* marks phloem parenchyma cells. **F**) *ProSUC2:PP2A1-mCherry* marks companion cells. **G**) *ProSEOR2:SEOR2-mCherry* makes sieve elements. Scale bars = 10 µm.

Lines 288-289: callose quantification is commonly done using a fixed tissue (see Lee et al 2011 https://pubmed.ncbi.nlm.nih.gov/21934146/). Live tissue staining may introduce bias of induced de novo callose synthesis which may not represent true resting levels of callose in those plants. Also, it would be better to use a different pseudo-color for callose signal throughout the paper to distinguish it from yfp/gfp.

In our experiments, we obtained more consistent results for callose staining without fixation. We always included controls for each experiment to account for the potential de novo callose synthesis. In our hands, we experienced more inconsistency with different fixation methods following the published protocols. It is worth noting that callose staining without fixation has been adopted in many recent publications from PD labs (Cui and Lee, 2016, https://www.nature.com/articles/nplants201634.pdf; Tee et al., 2023, https://www.pnas.org/doi/10.1073/pnas.2216397120; Huang et al., 2023; https://doi.org/10.1093/plcell/koad176).

We have changed the pseudo-color of callose into magenta.

Lines 289-294: in the statistical analysis of fig. 4 please include the comparison between pdlp6 and control.

We have included the comparison between *pdlp6* and control in Figure 4B.

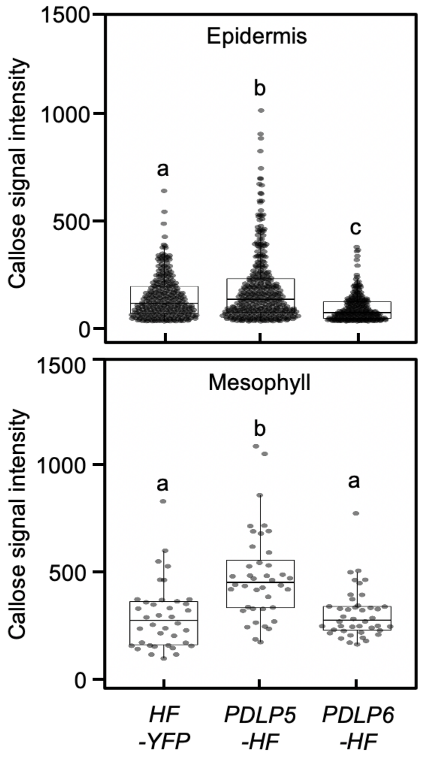

Figure 4B. Quantitative data show callose accumulation between epidermal cells and mesophyll cells. Quantitative data show callose accumulation between epidermal cells and mesophyll cells. Each dot represents an aniline blue-stained callose. *HF-YFP*, n = 283; *PDLP5-HF*, n = 359; and *PDLP6-HF*, n = 233 for epidermis. *HF-YFP*, n = 38; *PDLP5-HF*, n = 40; and *PDLP6-HF*, n = 42 for mesophyll cell. The number of images used for quantification are as follows: *HF-YFP*: 38; *PDLP5-HF*: 41; and *PDLP6-HF*: 36 for epidermis. *HF-YFP*: 34; *PDLP5-HF:* 37; and *PDLP6-HF:* 36 for mesophyll cell. Images were captured from three leaves, each from a different plant. Different letters on the bar indicate statistically significant differences analyzed with one-way ANOVA ($P < 0.0001$).

Lines 294-297: In fig. 4C the DIC channel images are not shown. The intensity plots in the lower panels cannot stand for quantification, these are simply replications of what is shown in the images above and therefore redundant.

DIC channel images have been added (Figure 4C in the revised manuscript). We analyzed 25 individual plants for each genotype and their fluorescence intensity profiles of callose accumulation were combined within the genotype. The lower panel shows the intensity profiles of all 25 plants (Figure 4D in the revised manuscript) rather than simply showing from the one image presented. Thus, they are not simply replicating what is shown in the image above.

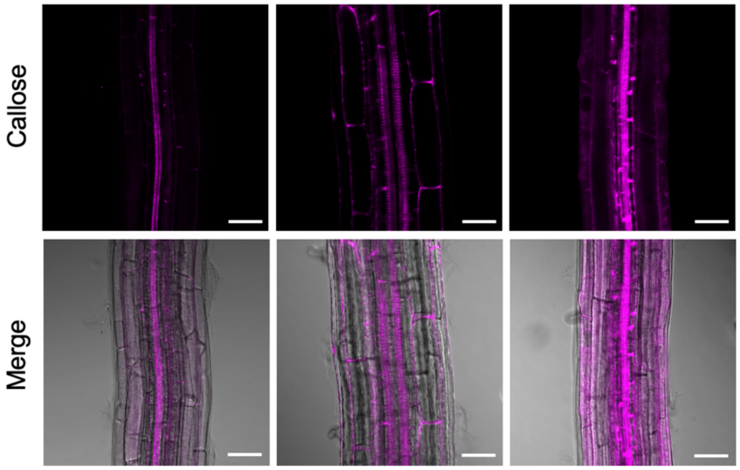

Figure 4C. Callose accumulation in roots. Magenta signals represent aniline blue stained-callose in roots (top panel). Merged images show the signals from callose and bright-field (lower panel). It is noted that the signals from xylem are autofluorescence. Scale bars = 50 μm.

Lines 316-318: this reasoning is in contrast to the data. Based on the OE phenotypes, it is already evident that PDLP5 and PDLP6 are themselves regulated differentially, i.e. they are functionally non-redundant proteins. If only the cell-specific factors determine the divergence between PDLP5 and PDLP6, then the OE phenotypes of the two proteins should be the same, but this is not the case.

We recognize that the statement might not be clear and straightforward enough. As stated earlier, the cell type-specific expression of PDLP6 is part of the equation. It also relies on cell type-specific functional partners SUS6 and CalS7 to exert its function. Our studies on the genetic interaction between PDLP6, SUS6, and CalS7 further support our conclusion.

To enhance the clarity of our statements, we have made the following revisions:

"The distinct starch accumulation patterns (Figure 2) and the differential regulation of plasmodesmata in various cell types (Figure 5) in *PDLP5-HF* and *PDLP6-HF* transgenic lines suggest that the ubiquitous expression of PDLPs does not uniformly impact plasmodesmal function. The findings also show that the impact of overexpressing PDLP5 and PDLP6 is most pronounced in the cell types where they are naturally expressed. As PDLPs are not predicted to catalyze callose biosynthesis, the enzymes or proteins function together with the PDLPs in synthesizing callose might also express in a cell type-specific manner." (lines 252-258 in the revised manuscript)

Lines 328-330: it is not clear why pUBQ10 was used instead of the p35S which was already used for the phenotype analysis. Does the pUBQ10-driven expression also lead to growth and starch phenotypes as the 35S? This info needs to be provided, especially since the results of the proximity labeling might be quite different depending on the strength of the exhibited phenotype of the plants.

We opted for *pUBQ10* instead of *p35S* because our constructs were based on previously published expression vectors (Mair et al., 2019, https://elifesciences.org/articles/47864). The *pUBQ10*-driven expression of PDLP6 leads to smaller plant size and lower fresh weight than the control; however, *pUBQ10:PDLP5-TbID* transgenic lines did not exhibit significant phenotypes. This could be attributed to relatively lower expression levels of the PDLP-TbID protein in these lines. Nevertheless, we successfully identified putative functional partners of the PDLPs. We agree with the reviewer that the expression level of the TbID fusion proteins might have a drastic effect on the proteins identified. We are happy with the data we obtained using the *pUBQ10* promoter. Instead of switching to the *p35S* promoter, we are trying to identify cell type-specific functional partners of PDLPs using their native promoters.

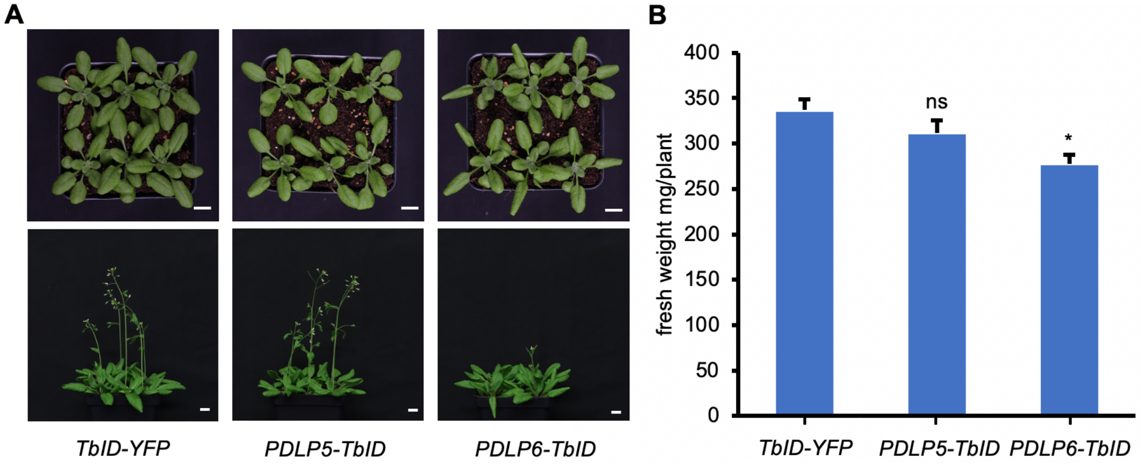

Growth phenotype of transgenic plants expressing TbID fusion proteins. A) upper panel: 4-week-old plants; lower panel: 5-week-old plants. *TbID-YFP*, *PDLP5-TbID*, and*PDLP6-TbID*refer to *ProUBQ10: 3xFlag-TurboID-EYFP*, *ProUBQ10:PDLP5-TurboID-3xFlag*, and *ProUBQ10:PDLP6-TbID-3xFlag,* and*,* respectively. **B**) Fresh weight of 4-week-old plants shown in **A**). The plot shows the mean with SD (n = 14). Asterisks indicate statistically significant differences (*t*-Test; two-paired; *P* < 0.01). ns: no significance.

Lines 336-337: please also include separate images for the callose signal in fig. 6A.

We have included separate images of callose signals in Figure 6B.

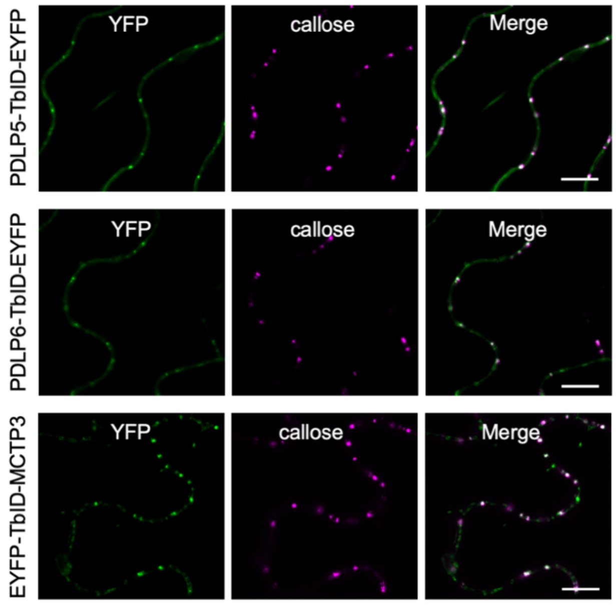

Figure 6B. Plasmodesmal localization of PDLP5-TbID-EYFP, PDLP6-TbID-EYFP, and EYFP-TbID-MCTP3. Agrobacteria harboring *ProUBQ10:PDLP5-TurboID-EYFP-3xFlag* (PDLP5-TbID-EYFP), *ProUBQ10:PDLP6-TurboID-EYFP-3xFlag* (PDLP6-TbID-EYFP), and *ProUBQ10:3xFlag-EYFP-TurboID-MCTP3* (EYFP-TbID-MCTP3) were infiltrated into *N. benthamiana* to transiently overexpress the EYFP fusion proteins. The plasmodesmal localization of the EYFP fusion proteins was imaged using confocal microscopy. Green signals represent the expression of the EYFP fusion proteins. Aniline blue stained-callose signal was shown in magenta. Merged images show the plasmodesmal localization of the EYFP fusion proteins. Scale bars = 10 μm.

In fig. 6B please indicate what parameter has been measured (e.g. Pearson coeff. of co-localization etc.), rather than "percent of overlap" which is not a standardly used parameter for co-localization quantification. Also, it is not clear what does n represent in this graph?

Pearson's coefficient analysis revealed that the expression of PDLP6 enhances the plasmodesmal localization of SUS6-sfGFP (Figure 7A-7C). Here, 'n' presents the number of confocal images.

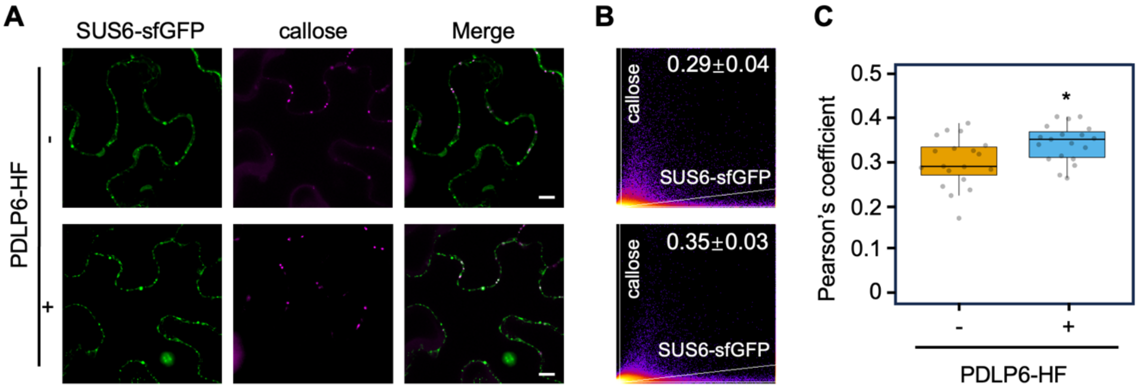

Figure 7A-7C. Transient overexpression of PDLP6-HF increased plasmodesmal association of SUS6-sfGFP. A) Confocal images show plasmodesmal localization of SUS6-sfGFP with or without overexpressing PDLP6-HF. The fusion proteins were transiently overexpressed in *N. benthamiana*. Aniline blue-stained callose marked plasmodesmata. Scale bars = 10 µm. **B-C**) Pearson's co-efficient analysis shows that the expression of PDLP6 increases the plasmodesmal localization of SUS6-sfGFP. Each dot represents Pearson's co-efficient value calculated for a merged image. n=19. Images were captured from three leaves. An asterisk indicates statistically significant differences analyzed with a Mann-Whitney *U* Test (*, $P < 0.05$).

Line 337: here and elsewhere, please avoid the term "physical interaction" as it implies direct interaction tested in vitro. Only in planta data is provided.

To further determine the direct physical interaction between PDLP6 and SUS6, we conducted an in vitro pull-down assay. Figure 7E in the revised manuscript shows that a biotinylated C-terminal tail of PDLP6, which faces the cytoplasmic sleeve, can specifically pull down the maltose binding protein-SUS6 (MBP-SUS6) fusion proteins and MBP-CalS7-cytoplasmic loop (MBP-CalS7-CL) fusion proteins (Figure 7E). Together, the findings confirmed the physical interaction between PDLP6 and SUS6 as well as PDLP6 and CalS7.

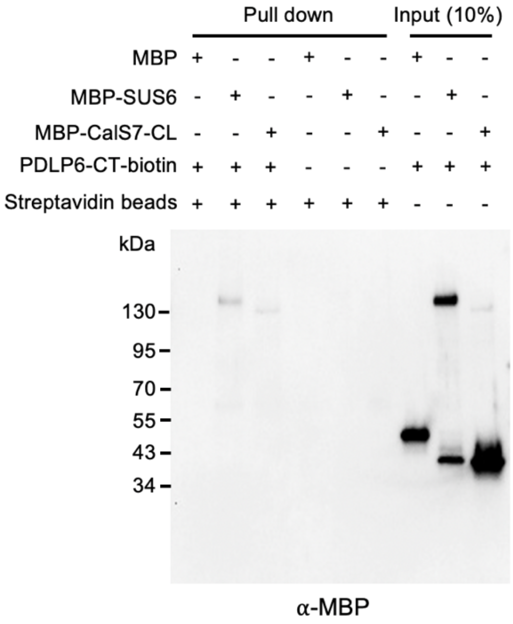

Figure 7E. In vitro pull-down assay. Direct interaction between PDLP6 and SUS6, and PDLP6 and CalS7. The biotinylated C-terminal tail of PDLP6 (PDLP6-CT-biotin) was incubated with recombinant proteins, MBP, MBP-SUS6, or MBP-CalS7. Magnetic beads coupled with streptavidin were used to pull down PDLP6-CT-biotin and the interacting proteins. A MBP antibody was used to detect the interaction between PDLP6 and MBP fusion proteins.

In fig. 6C it would be helpful to include PDLP5 as well in the co-IP analysis, as this would better demonstrate/confirm whether there's divergence between the two PDLPs.

We conducted the co-IP analysis as suggested by the reviewer. We detected the interaction between PDLP5 and SUS6, as well as CalS7, through co-IP and in vitro pull-down assays. Since SUS6 and CalS7 are specifically expressed in the vasculature, whereas PDLP5 is not expressed in this tissue, the observed interactions may not have biological relevance in planta. We could not conclude whether there is a divergence between the two PDLPs' protein activity only based on their interaction with SUS6 and CalS7.

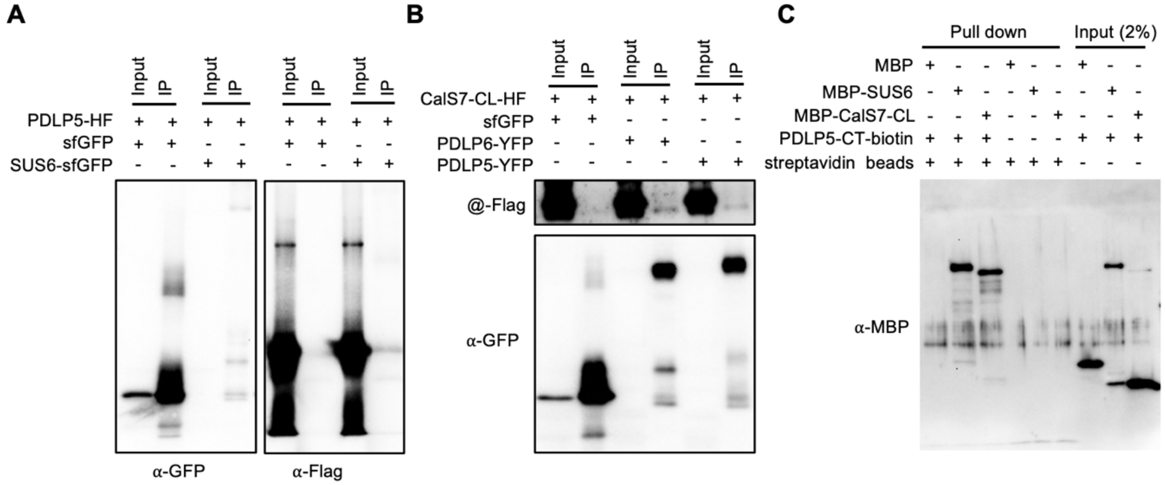

Interaction between PDLP5, SUS6, and CalS7. A-B) Co-IP assays show the interaction between PDLP5, SUS6, and CalS7. Agrobacteria harboring different plasmids were co-infiltrated into *N. benthamiana*. Samples were collected 2 days after infiltration and subjected to Co-IP. **C**) In vitro pull-down assay shows the direct interaction between PDLP5, SUS6, and CalS7. The biotinylated C-terminal tail of PDLP5 (PDLP5-CT-biotin) was incubated with recombinant proteins, MBP, MBP-SUS6, or MBP-CalS7. Magnetic beads coupled with streptavidin were used to pull down PDLP5-CT-biotin and the interacting proteins. A MBP antibody was used to detect the interaction between PDLP6 and MBP fusion proteins.

Lines 423-426: again, this explanation is missing out on the point that it is at the protein level that the two PDLPs diverge.

PDLP5 is highly expressed in all cell types, including the vasculature, in *p35S:PDLP5-YFP* (Supplemental Figure 4). The findings suggest that PDLP5 is also highly expressed in the vasculature of *PDLP5-HF*; however, the absence of callose overaccumulation in *PDLP5-HF* vasculature (Figures 4C and 4D) suggests that PDLP5 may not function with SUS6 and CalS7 to regulate plasmodesmal function. The observed interaction between PDLP5, SUS6, and CalS7 using co-IP and in vitro pull-down assays might not translate to the biological function in the transgenic plant.

Line 436: As PDLPs are PM-associated, their cell-to-cell movement is quite unlikely.

Our recent findings showed that HopAF1, a PM-associated protein effector from *Pseudomonas syringae* pv. *tomato* DC3000, can efficiently traffic through plasmodesmata when transiently expressed in *N. benthamiana* (Li et al., 2021, Front Plant Sci. 12, 640277.). Moreover, HopAF1^{G2A}, which can't be associated with the PM due to the mutation of the *N*-myristoylation site, is not as mobile as the PM-associated HopAF1, suggesting that PM association of HopAF1 does not

negatively affect the PD-dependent movement of the protein. The findings support the notion that the PM-associated PDLP6 might be able to move from one cell to its neighbors.

Lines 444-453: the growth retardation, Pd callose and starch accumulation phenotypes are not limited to PDLP overexpression only. It has been reported for other Pd-proteins as well (see Zavaliev et al 2010, https://pubmed.ncbi.nlm.nih.gov/19887501/). The likely explanation of the OE phenotype is the Pd restriction that results either from overaccumulation of a Pd protein, or induction of callose as a secondary stress response to Pd malfunction, or both.

We agree with the reviewer that the reduction in plasmodesmata permeability in these overexpression lines could be attributed to changes in their protein composition or callose overaccumulation, or both. Given that SUS6 and CalS7, two proteins involved in callose synthesis, are required for PDLP6 to function (Figure 7 in the revised manuscript), it is reasonable to assume that the phenotypes observed in PDLP6 overexpression lines result from, if not solely, primarily callose overaccumulation at plasmodesmata.

We have added the following statement:

"Interfering with the movement of molecules through plasmodesmata can severely impact plant growth and development (Kim et al., 2005; Thomas et al., 2008; Guseman et al., 2010; Zavaliev et al., 2010; Lee et al., 2011; Benitez-Alfonso et al., 2013; Dettmer et al., 2014; Brunkard et al., 2020). For instance, the overexpression of the plasmodesmal-associated class 1 reversibly glycosylated polypeptide in Arabidopsis results in suppressed plasmodesmal function, inhibited plant growth, and an hyperaccumulation of starch in *Nicotiana tabacum* (Zavaliev et al., 2010)." (lines 393-398 in the revised manuscript)

Lines 512-514: this statement is confusing. No SA levels have been correlated with starch accumulation either in PDLP5 OE or PDLP6 OE, so this discussion on the role of SA is too speculative.

We have removed the part concerning PDLP5 and salicylic acid.

Dr. Kyaw Aung
Iowa State University
Ames, Iowa

Dear Dr. Aung:

We have received reviews of your manuscript entitled "Plasmodesmata-located protein 6 regulates plasmodesmal function in Arabidopsis vasculature." While all reviewers acknowledge a substantial improvement in your manuscript with this revised version, there are lingering concerns. Specifically, some of the previously highlighted issues, such as the argument regarding the cell type-specific function of PDLP5 and PDLP6, have not been sufficiently addressed. Additionally, there are concerns about the interpretation of TurboID results and the utilization of essential controls in multiple experiments. Lastly, Reviewers have also noted the presence of contradictory data and information within the manuscript.

It is crucial to highlight that this marks the second revision request. I must express concern that in the event the resubmission still does not adequately and comprehensively address the reviewers' concerns, we may be unable to provide an additional opportunity for further revisions to your manuscript. Given the nature of the comments, we are offering you 60 days from when we have issued this decision to complete the revision. If a revision is not returned within this time frame, and if you have not been granted an extension, we will withdraw the manuscript, which will leave you free to submit the work elsewhere. If you need an extension, we encourage you to contact us at any point before the 60 days have passed.

When you are ready to submit the revised version, please upload a highlighted copy that indicates all changes made in response to the editor and reviewer recommendations. Include an itemized list of all changes made in response to each of the reviewer's suggestions in the "Response to Reviewers" section; please note that reviewers do not have access to your cover letter, nor was this decision letter shared with them.

Thank you for the privilege of reviewing your work. We look forward to receiving your revised manuscript.

Sincerely,

The Plant Cell Editorial Board

Please note the following:

-The Plant Cell now requires authors to complete and submit an author revisions checklist upon submission of a revised manuscript. The aim of the checklist is to aid authors in preparing a high-quality manuscript, facilitate the review and assessment of revised manuscripts, and help to ensure that journal standards are maintained across the board. If your manuscript is accepted, the completed checklist will be published as supplemental material attached to the article online. Please download a copy of the checklist (pdf fillable form) at this link, for submission with your revised manuscript: https://tpc.msubmit.net/html/Author_Revisions_Checklist.pdf.

-Supplemental materials should be restricted to large datasets and tables, presentation of replicates, and validation of reagents, methods, or genotypes. Any data that are used to support the major claims must be in the main manuscript. Supplemental figure legends must indicate what figure in the main manuscript is supported by the supplemental data presented. Please justify how each of the supplemental figures meet the criteria.

-Sampling methods and nature of "biological replicates" should be described precisely (i.e. different plants, parts of plants, pooled tissue, independent pools of tissue, sampled at different times, etc), along with a clear description of and rationale for any statistical analyses conducted. The reader should know exactly what was sampled; what forms the basis of the calculation of any means and statistical parameters reported. This is also necessary to ensure that proper statistical analysis was conducted.

-Want to add this revision deadline to your calendar? Click below!

-- Reviewer comments:

Reviewer #1 (Comments for the Author):

First, I am delighted to see the revised manuscript with several substantive improvements to the experimental design and text. This draft is much more accessible to read, which allowed me to focus more carefully on the exciting findings. I also greatly appreciate the authors' willingness to adjust statements and experimental approaches throughout the text, which I know can be a frustrating process, so I'm going to avoid adding too much new here. That said, because the manuscript has been substantially revised, I can now really focus more precisely on a few points, which I'll raise below and which I hope will be easily addressed.

Cell type specificity: As came up in other reviews last time, I'm still a little bit confused about the cell type specificity argument being made here.
In the TurboID data, PDLP6 maybe slightly enriches for SUS6, but PDLP5 also enriched for SUS6. CalS7 wasn't detected in the TbID data, but CalS1 was-and was somewhat enriched in both PDLP5 and PDLP6 datasets. Long before we get to either of these, the list of strongly enriched putative PDLP5 and PDLP6 interactors includes a wide range of plasma membrane proteins, such as permeases, that have nothing to do with callose synthesis. My point is that the authors have chosen to focus on this narrative, but it isn't obvious to me that the data support that the PDLP6-SUS6-CalS7 interaction is so interesting or biologically meaningful. (This is what I intended, and maybe didn't state clearly enough, in the last review: I'm perfectly supportive of the statistical cut-offs used by the authors, but the decision to focus narrowly on these proteins that aren't among the best-supported putative interactors seems potentially misleading.) In any case, given that PDLP5 apparently also strongly enriches for SUS6 over the negative control, I'm not convinced that this proposed interaction confers any cell type-specific effects or explains the overall differences between PDLP6-HF and PDLP5-HF phenotypes. Similarly, CalS1 comes up as enriched by both PDLP5-HF and PDLP6-HF (I don't know the p-value, but it's about a two-fold increase in the supplemental data, by eye), but CalS7 did not; why, then, the focus on CalS7, unless the point was to force this interpretation of cell type specificity?
I'll put this another way. The argument seems to shift among three hypotheses that are somewhat contradictory: (1) PDLP5 and PDLP6 are transcriptionally regulated to be expressed predominantly in distinct cell types, which confers cell type-specificity to their mechanism of action; (2) PDLP5 and PDLP6 are functionally/biochemically equivalent, but their interacting partners are expressed in distinct cell types, which confers cell type-specificity to their mechanism of action; (3) PDLP5 and PDLP6 are functionally/biochemically different, with PDLP6 only able to interact with SUS6/CalS7, which is why PDLP6 is only fully functional in the vasculature.
On point 1, if this were true, then why does the 35S promoter cause such a strong phenotype for PDLP6, which is the original discovery? Here, PDLP6 is being expressed everywhere, and it's really detrimental.
On point 2, if this were true, then why doesn't PDLP6 function equivalently in the epidermis, and PDLP5 function equivalently in the vasculature?
On point 3, if this were true, then why does PDLP5 nonetheless strongly enrich for SUS6 over negative controls, and why doesn't CalS7 show up in the TurboID experiments (but CalS1 does)?
I am sure that the authors could respond to each of these three points separately and convincingly, but I hope that they will consider revising the paper further to move away from their singular interpretation to instead allow for some alternative hypotheses.

Figure 5E/F: Measuring this phenomenon is tricky, so I am very glad to see the authors taking creative approaches to try to test whether PDLP6 impacts PD trafficking in specific cell types is affected. I do think there are many ways in which this assay might not be ideal; among other things, it's hard to know if there might be feedback onto the PDLP6 promoter or effects of the different growth phenotype of PDLP6 overexpression lines on overall source/sink dynamics and cell-cell trafficking. That said, I think this is sufficient evidence for me to make the claim that PDLP6 impacts trafficking of YFP, as long as the authors are careful not to overinterpret.
As a side note: the authors mention the work from Imlau et al. showing trafficking of SUC2pro:GFP as an alternative strategy. Why did the authors not use this established experimental approach?

Callose quantification: The aniline blue staining protocol used here is not ideal; among other things, aniline blue only specifically stains callose in basic solutions (pH > 12), not at pH = 7.4, so PBS is not an appropriate buffer, and some of the fluorescence observed may be artifactual / non-specific. There's a paper that will be published very soon from Sankoh et al. in the Burch-Smith Lab demonstrating that the method of callose quantification used here is not reliable or reproducible. That said, I fully understand that this has a been a common (if not ideal) approach in the field for the past several years, so I do understand why this protocol was used. If it weren't so important to the conclusions being drawn in this paper, I wouldn't be so worried-but in this revised manuscript, callose synthesis at PD seems to be the central argument about how PDLP6 is functioning. I would encourage the authors to at least mention that the protocol used is not optimal, and to consider whether repeating any key experiments using the protocols validated by Sankoh et al. would improve the reliability of these experimental conclusions. Doi: 10.1101/2023.09.30.560305.
Incidentally, the data shown in the response to my last review are not encouraging: propidium iodide seems to be staining precisely the same regions as the aniline blue signal! The bright aniline blue-stained "puncta" correspond perfectly to bright puncta of PI staining. This makes me very concerned that the aniline blue stain is nonspecifically binding some other molecule, as expect at the near-neutral pH used.

Lastly, one minor suggestion, in the spirit of inclusivity and sensitivity: LL410 and 414, I recommend rephrasing as "...overexpression of PDLP6 delays plant growth" (or "leads to delayed", or "slows") and then as "...demonstrated that the slowed plant growth phenotype" (or "delayed plant growth", etc.).

Reviewer #2 (Comments for the Author):

The revised version of this manuscript is much improved over the original manuscript. The narrower focus and fewer extraneous claims have streamlined the manuscript and made much clearer the points the authors want to have readers focus on. In particular, the new pull-down assays support the claims of direct physical interaction between PDLP6 and SUS6 (Figure 7). This has certainly improved the manuscript.

Unfortunately, several of the major claims of the manuscript remain unsupported by the data presented. A close examination of Supplemental Dataset 1 and Supplemental Figure 5 reveal that SUS6 was enriched in the MCTP3, PDLP5 and PDLP6 Turbo-ID samples. This contrasts with statements made in the manuscript, lines 317-318. The data presented in Figure 7 is supposed to support the authors' model that a PDLP6-SUS6-CalS7 complex exists in the phloem and there it acts to regulate plasmodesmata by determining callose homeostasis. This complex is proposed to explain the cell type-specific effects observed on overexpression of PDLP5 or PDLP6. But where is the control experiment to support that the interaction with SUS6 is specific to PDLP6? The association of SUS6 with PDLP5, and other plasmodesmal proteins for that matter, cannot be ruled out based on the current data. A pulldown or co-IP with PDLP5 should also be presented to verify the specificity of the PDLP6-SUS6 complex. Further, PDLP6 and PDLP7 have overlapping expression patterns (lines 391-394). Therefore, before claims of a specific or exclusive interaction between PDLP6 and SUS6 can be made, the interaction between PDLP7 and SUS6 should be tested. And why exclude SUS5 from the story if it has a similar expression pattern to SUS6 (line 423)?

The manuscript text needs thorough editing and polishing. It is standard practice that results are written in the past tense, and this is not done here. Also by convention, plant protein names are written in capital letters, so 'PLASMODESMATA-LOCATED PROTEIN (PDLP)' and not 'plasmodesmata-located proteins (PDLP)'. Probably due to the extensive revision, the text has become quite choppy and difficulty to follow.

As far as is known to date, all PDLPs have one function: to regulate callose homeostasis at plasmodesmata. This leads to them having different physiological roles depending on how they are recruited by the system in question. Lines 75-76 should be more carefully re-stated.

Reviewer #3 (Comments for the Author):

**In the revised manuscript the authors successfully addressed some of the comments, but provided unclear/speculative responses to others. The authors maintain that the difference between pdlp5 and pdlp6 is due to cell-specific factors. This is overinterpretation and it disregards the over-expression data. The authors show that the Pd localization and the expression levels of the two proteins are very similar in the over-expression lines. Yet, the phenotypes these proteins produce are very different, particularly Pd-callose and starch levels. So, what is the conclusion from these overexpression data? The conclusion is that the two PDLPs are biochemically different. Regardless of the downstream cell-specific factors, these pdlps function differently. In other words, at this point the findings do not explain why overexpression of pdlp6 does not induce callose synthesis in the epidermis and mesophyll like pdlp5 does; and why pdlp5 overexpression does not induce callose in vasculature like pdlp6 does. I can be even more deliberate: for example, why pdlp6 overexpression does not pull down the PIP proteins in the TbID? And so on.
These questions have to be somehow posed and discussed in the manuscript before interpreting what do pdlp5/6 do in their natively expressed tissues.**

In fig. 7d please indicate "IP-GFP" instead of just "IP". It takes a while to guess what ab was used for IP. The legend also doesn't say what IP was done.

In fig. 7E please mark and explain the non-specific bands for MBP-SUS6 and MBP-Cals, especially since these are shorter than the free MBP.

In fig. 7a, the callose images do not match the corresponding gfp images.

Lines 276-282: It would be useful to include the data about the phenotype of pUBQ10 transgenic plants used for turbo-ID would in the manuscript, not just in the response letter. The protein levels provided in the supplemental data are not telling us what is the phenotype of those plants.

Lines 399-402: this is speculation based on unrelated evidence.
The movement of the bacterial effector Hopaf1 between cells cannot serve as evidence to suggest movement of the host intrinsic transmembrane Pd proteins like PDLPs. As a putative myristoylated protein, Hopaf1 is predicted to be anchored to the PM, rather than being transmembrane. It could be that these PM-anchored effectors are cleaved at the Pd vicinity before they enter the channel in the soluble form. It is known that by itself myristoylation promotes transient membrane association enabling proteins to anchor to membranes but dissociate easily. More importantly, the effector hopaf1 shown in Li et al (2021) is not enriched at the Pd as the PDLPs are. To the best of my knowledge, no transmembrane Pd proteins have yet been reported to move cell-to-cell.

Overview

We are grateful once again for your invaluable feedback on our revised manuscript. Your insightful comments have significantly enhanced the quality of our work. In response to your feedback, we have implemented several minor updates to the manuscript:

1. We have introduced a new set of aniline blue-stained callose images in Figure 4, utilizing basic solutions (pH 12). The staining method allows the detection of callose accumulation in the vasculature of Arabidopsis leaves, which aligns more closely with the focus of our manuscript on leaf phenotypes. Consequently, we replaced the callose data collected from Arabidopsis roots.

2. We have expanded our examination of physical interactions among proteins by including additional proteins in co-IP and in vitro pull-down assays.

3. The discussion section has been revised to provide a clearer explanation of our working hypothesis, and we have considered an alternative explanation as suggested by the reviewers.

4. Any findings perceived as contradictory by the reviewer have been addressed and clarified for better understanding.

5. Minor edits have been made to the text to enhance the overall flow and readability of the manuscript.

Thank you once again for your invaluable input, which has significantly contributed to the refinement of our work.

Reviewer #1 (Comments for the Author):

First, I am delighted to see the revised manuscript with several substantive improvements to the experimental design and text. This draft is much more accessible to read, which allowed me to focus more carefully on the exciting findings. I also greatly appreciate the authors' willingness to adjust statements and experimental approaches throughout the text, which I know can be a frustrating process, so I'm going to avoid adding too much new here. That said, because the manuscript has been substantially revised, I can now really focus more precisely on a few points, which I'll raise below and which I hope will be easily addressed.

Cell type specificity: As came up in other reviews last time, I'm still a little bit confused about the cell type specificity argument being made here.
In the TurboID data, PDLP6 maybe slightly enriches for SUS6, but PDLP5 also enriched for SUS6. CalS7 wasn't detected in the TbID data, but CalS1 was-and was somewhat enriched in both PDLP5 and PDLP6 datasets. Long before we get to either of these, the list of strongly enriched putative PDLP5 and PDLP6 interactors includes a wide range of plasma membrane proteins, such as permeases, that have nothing to do with callose synthesis. My point is that the authors have chosen to focus on this narrative, but it isn't obvious to me that the data support that the PDLP6-SUS6-CalS7 interaction is so interesting or biologically meaningful. (This is what I intended, and maybe didn't state clearly enough, in the last review: I'm perfectly supportive of the statistical cut-offs used by the authors, but the decision to focus narrowly on these proteins that aren't among the best-supported putative interactors seems potentially misleading.) In any case, given that PDLP5 apparently also strongly enriches for SUS6 over the negative control, I'm not convinced that this proposed interaction confers any cell type-specific effects or explains the overall differences between PDLP6-HF and PDLP5-HF phenotypes. Similarly, CalS1 comes up as enriched by both PDLP5-HF and PDLP6-HF (I don't know the p-value, but it's about a two-fold increase in the supplemental data, by eye), but CalS7 did not; why, then, the focus on CalS7, unless the point was to force this interpretation of cell type specificity?

We appreciate the valuable feedback provided by the reviewer. We are delighted that the reviewer has identified numerous significant improvements in our previous version. In response to the reviewer's comment, we have included the requested callose staining data in this version and revised our hypothesis in alignment with the suggestions provided.

We moved the description of the justification of the statistical cut-offs of the proximity labeling assay from the main text to the materials and methods to improve the manuscript's flow further. We also removed the Histogram of q values (Supplemental Figure 6 in the previous version) as the figure is not essential for the manuscript.

We acknowledge the reviewer's perspective; nevertheless, we are enthusiastic about employing a comprehensive array of methodologies, including cell biology, biochemical analyses, and genetic approaches, to provide empirical evidence regarding the collaborative role of PDLP6 alongside SUS6 and CalS7 in regulating plasmodesmal function in the phloem.

I'll put this another way. The argument seems to shift among three hypotheses that are somewhat contradictory: (1) PDLP5 and PDLP6 are transcriptionally regulated to be expressed predominantly in distinct cell types, which confers cell type-specificity to their mechanism of action; (2) PDLP5 and PDLP6 are functionally/biochemically equivalent, but their interacting partners are expressed in distinct cell types, which confers cell type-specificity to their mechanism of action; (3) PDLP5 and PDLP6 are functionally/biochemically different, with PDLP6 only able to interact with SUS6/CalS7, which is why PDLP6 is only fully functional in the vasculature.

Thank you for nicely putting together three hypotheses to explain our work. Our working hypothesis is a combination of the first two points. Using PDLP6 as an example, it is transcriptionally regulated to be expressed predominantly in the vasculature. In addition, the function of PDLP6 depends on the presence of its functional partners, CalS7 and SUS6, in the vasculature. It explains why the ubiquitous expression of PDLP6 does not lead to callose overaccumulation of all cell types. However, our findings do not rule out the possibility that PDLP5 and PDLP6 are biochemically different. We revised the second and third paragraphs of the discussion as the following:

"We hypothesize that for PDLP6 to function predominantly in phloem, two key regulations are necessary: (1) transcriptional regulation of *PDLP6* expression in phloem and (2) expression of PDLP6's functional partners in the same cell types. A previous report has shown that different members of *PDLP* transcripts were detected in distinct Arabidopsis leaf cell types, with PDLP6 identified in phloem parenchyma cells (Kim et al., 2021). We detected PDLP6-YFP fusion protein in phloem, including phloem parenchyma cells, companion cells, and sieve elements (Figures 3C-3G). Conversely, PDLP5 was primarily observed in epidermal cells (Figure 3A-3B). These findings support the transcriptional regulation of PDLPs to express them predominantly in distinct cell types. Using a proximity labeling assay, we identified SUS6 as a potential functional partner of PDLP6 (Figure 6E). Biochemical approaches confirmed the physical interaction between PDLP6 and SUS6 (Figures 7D-7E), and genetic analysis established the dependence of PDLP6 function on SUS6 (Figure 7F). Additionally, we demonstrated physical and genetic interactions between PDLP6 and CalS7 (Figures 7D-7E and 7G). Given that SUS6 and CalS7 are predominantly expressed in the phloem (Barratt et al., 2011; Xie et al., 2011; Yao et al., 2020; Kalmbach et al., 2023), our findings suggest that PDLP6 functions with SUS6 and CalS7 to regulate plasmodesmal function in the vasculature.

However, our findings do not rule out the possibility that PDLP5 and PDLP6 are biochemically different. While we detected the physical interaction among PDLP5, SUS6, and CalS7 using co-IP and in vitro pull-down assays (Supplemental Figure 7), the overexpression of PDLP5 does not appear to promote callose accumulation in the vasculature (Figure 4). If the overexpressed PDLP5 can interact with SUS6 and CalS7 in planta, the physical interaction alone may not be sufficient for PDLP5 to function with SUS6 and CalS7 to regulate callose accumulation. Given that PDLP5 is predominantly expressed in non-overlapping cell types compared to SUS6 and CalS7, the observed physical interactions may not hold biological relevance under their native conditions. Additional investigations are necessary to clarify why the overexpression of PDLP5 did not affect plasmodesmal callose accumulation and function in the vasculature."

On point 1, if this were true, then why does the 35S promoter cause such a strong phenotype for PDLP6, which is the original discovery? Here, PDLP6 is being expressed everywhere, and it's really detrimental.

The *PDLP5-HF* transgenic plants we characterized may not strongly inhibit plasmodesmal function. While the delayed plant growth phenotype was observed, the starch overaccumulation phenotype of PDLP5-HF only became prominent under higher light intensity. Alternatively, the biochemical activity of PDLP5 might not be as high as PDLP6. *PDLP6-HF*, on the other hand, might have a stronger impact on inhibiting plasmodesmal function, and the blockage of sugar movement in the vasculature in PDLP6-HF could be more detrimental to plant growth.

On point 2, if this were true, then why doesn't PDLP6 function equivalently in the epidermis, and PDLP5 function equivalently in the vasculature?

We consider PDLP5 and PDLP6 functionally equivalent, as the overexpressors showed callose overaccumulation in different cell types. However, we do not rule out the possibility that PDLP5 and PDLP6 are biochemically different. Given the unknown biochemical activity of PDLPs, we lack the means to determine whether PDLP5 and PDLP6 are biochemically equivalent. We hypothesize that PDLP5 and PDLP6 may interact with different functional partners in distinct cell types to exert their function to regulate callose accumulation. Here, we provided working models to explain the function of PDLP5 and PDLP6 (not included in the manuscript). We hypothesize that for PDLP6 to function, it requires SUS6 and CalS7. Given that the two proteins are only predominantly expressed in the vasculature, the *PDLP6-HF* overexpressor mainly affects the plasmodesmal callose accumulation and function in the same tissue. Physical and genetic interactions support the hypothesis that PDLP6 functions with SUS6 and CalS7 to regulate the plasmodesmal function in the vasculature (Figure 7). As none of the SUSs are expressed in the epidermal and mesophyll cells (Yao et al., 2019; https://academic.oup.com/jxb/article/71/6/1858/5660925?login=true), PDLP5 likely functions through the cytosolic invertase (CINV) pathway to regulate callose accumulation in the epidermal and mesophyll cells. Our proximity labeling assay identified PIP5K9 (AT3G09920; Supplemental Data Set 1_TbID data sheets), which physically interacts with CINV1 (Lou et al., 2009; https://academic.oup.com/plcell/article/19/1/163/6091529?login=true), as a functional partner of PDLP5 and PDLP6, but not MCTP3. We are pursuing the potential role of PIP5K9 and cytosolic invertases (CINVs) in regulating callose accumulation in collaboration with PDLP5, which is beyond the scope of this manuscript.

We included additional data showing that PDLP5 also physically interacts with SUS6 and CalS7 in our co-IP and in vitro pull-down assays, whereas PDLP7 does not (Supplemental Figure 7). Despite the positive physical interaction, the PDLP5-HF overexpressor does not lead to the overaccumulation of plasmodesmal callose in the vasculature. If PDLP5 physically interacts with SUS6 and CalS7 in plants, the interaction among the proteins might not be sufficient to promote plasmodesmal callose accumulation. Further investigations (e.g., a genetic interaction assay) are required to test the hypothesis.

On point 3, if this were true, then why does PDLP5 nonetheless strongly enrich for SUS6 over negative controls, and why doesn't CalS7 show up in the TurboID experiments (but CalS1 does)?
I am sure that the authors could respond to each of these three points separately and convincingly, but I hope that they will consider revising the paper further to move away from their singular interpretation to instead allow for some alternative hypotheses.

The findings that PDLP5 (and MCTP3) enriched SUS6 over negative controls underscore the strengths and limitations of the proximity labeling assay. This assay typically detects proteins within proximity (approximately 20 nm) to the enzyme (TurboID). In most plasmodesmal regions, the plasma membrane and ER membrane are within this range (Nicolas et al., 2017; https://www.nature.com/articles/nplants201782), suggesting that various PD-associated proteins would likely enrich similar proteins functioning in proximity to PD-localized proteins compared to negative controls. Moreover, the overexpression of PD-associated proteins fused with TbID enhances the identification of similar proteins that function at or near plasmodesmata.

Given that the overexpression of PDLP5 and PDLP6 resulted in their localization to the plasma membrane in addition to the plasmodesmata, the proximity labeling assay significantly increases the possibility of identifying a wide array of plasma membrane proteins, particularly those highly expressed. Thus, we were careful not to categorize all the candidate proteins as

PDLP-interacting plasmodesmal proteins. Despite their unclear role in plasmodesmal regulation, we were intrigued by the notable enrichment of permeases. Due to their structural similarity to PIPs, we speculate that they may indeed be PDLP-interacting proteins on either the plasma membrane or plasmodesmata. Using a split-ubiquitin yeast two-hybrid assay, we confirmed the physical interaction between PDLP5 and PIPs (data not shown), suggesting that PIPs physically interact with PDLP5. However, further investigations are needed to demonstrate the interaction between permeases with PDLP5 and their function at plasmodesmata. Conversely, some PDLP-interacting plasmodesmal proteins might be expressed only in specific cell types or expressed at low levels, potentially resulting in less pronounced enrichment in the proximity labeling assay (e.g., SUS6) or even undetected (e.g., CalS7).

We added the following statements to the result (lines 307-312).

"It is noted that PDLP5 and MCTP3 also significantly enriched SUS6 when compared to Col-0 control; however, PDLP6 showed the highest level of enrichment (Supplemental Figures 5D-I). Furthermore, PDLP6 showed a significant enrichment of SUS6 compared to both PDLP5 and MCTP3, whereas PDLP5 did not exhibit a significant enrichment of SUS6 compared to MCTP3 (see Supplemental Figures 5D-5E)."

The enrichment of SUS6 by PDLP5, PDLP6, and MCTP3 supports the notion that a portion of SUS6 localizes and functions around plasmodesmata. This enrichment of SUS6 by PDLP5 compared to a negative control was observed only when PDLP5 is overexpressed. Using *proPDLP5:PDLP5-TbID*, we did not enrich SUS6 compared to *pro35S:YFP-TbID* (data not shown). Nonetheless, PDLP6 enriches more SUS6 than PDLP5 (Figure 6C and Supplemental Figures 5D-I).

We were also intrigued by the slight enrichment of CalS1 by PDLP5, PDLP6, and MCTP3 compared to wild-type Col-0. We examined the physical interaction between CalS1, PDLP5, and PDLP6 (Supplemental Figure 7G) and added the following statements in the result (lines 356-367).

"CalS1 has been demonstrated to play an important role in regulating callose biosynthesis and plasmodesmal function in Arabidopsis (Tee et al., 2023; Cui and Lee, 2016). Our proximity labeling assay showed that TbID-MCTP3, PDLP5-TbID, and PDLP6-TbID significantly enriched a CalS protein group, including CalS1, CalS2, and CalS4, compared to wild-type Col-0. However, PDLP5-TbID and PDLP6-TbID did not significantly enrich the CalS protein group compared to TbID-MCTP3 (Supplemental Figure 5I and Supplemental Data Set 1). To examine the physical interaction among CalS1, PDLP5, and PDLP6, we performed an in vitro pull-down assay. As the cytoplasmic loop (CL) of CalSs was predicted to interact with SUS6 to form callose synthase complex (Verma and Hong, 2001; De Storme and Geelen, 2014), we cloned and purified the CL of MBP-CalS1 (MBP-CalS1-CL). Supplemental Figure 7G showed that PDLP5-CT-biotin and PDLP6-CT-biotin did not pull down MBP-CalS1-CL. The findings suggest that CalS1 might not be a strong interacting protein of PDLP5 and PDLP6, at least not with the CalS1-CL."

Supplemental Figure 7G showed that PDLP7-CT-biotin pulls down a free MBP. Thus, the positive interaction between PDLP7-CT-biotin and MBP-CalS1-CL might be mediated through MBP.

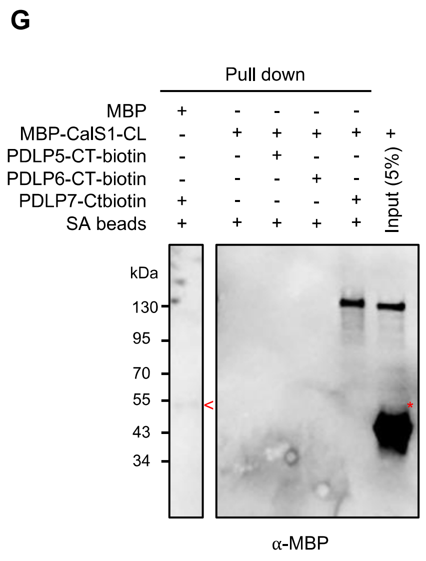

Supplemental Figure 7. G) In vitro pull-down assay shows no direct interaction between PDLP5-CalS1 and PDLP6-CalS1. The in vitro pull-down assay was conducted as mentioned above. An arrow indicated the binding of PDLP7-CT-biotin with a free MBP. Asterisks indicated non-specific bands for MBP-SUS6 and MBP-CalS7-CL.

Figure 5E/F: Measuring this phenomenon is tricky, so I am very glad to see the authors taking creative approaches to try to test whether PDLP6 impacts PD trafficking in specific cell types is affected. I do think there are many ways in which this assay might not be ideal; among other things, it's hard to know if there might be feedback onto the PDLP6 promoter or effects of the different growth phenotype of PDLP6 overexpression lines on overall source/sink dynamics and cell-cell trafficking. That said, I think this is sufficient evidence for me to make the claim that PDLP6 impacts trafficking of YFP, as long as the authors are careful not to overinterpret. As a side note: the authors mention the work from Imlau et al. showing trafficking of SUC2pro:GFP as an alternative strategy. Why did the authors not use this established experimental approach?

Measuring this phenomenon poses significant challenges for us. After dedicating over a year to generating the materials and establishing the method, we are pleased that it has yielded sufficient evidence to show that PDLP6 influences the trafficking of YFP out of the vasculature. We carefully phrase our findings to avoid any potential overinterpretation.

We borrowed the concept of using SUCpro:GFP from Imlau et al. instead of using the established method as we aimed to express GFP in the same cell type as PDLP6 under “native condition” instead of expressing them only in the companion cells.

Callose quantification: The aniline blue staining protocol used here is not ideal; among other things, aniline blue only specifically stains callose in basic solutions (pH > 12), not at pH = 7.4, so PBS is not an appropriate buffer, and some of the fluorescence observed may be artifactual / non-specific. There's a paper that will be published very soon from Sankoh et al. in the Burch-Smith Lab demonstrating that the method of callose quantification used here is not reliable or reproducible. That said, I fully understand that this has a been a common (if not ideal) approach in the field for the past several years, so I do understand why this protocol was used. If it weren't so important to the conclusions being drawn in this paper, I wouldn't be so worried-but in this revised manuscript, callose synthesis at PD seems to be the central argument about how PDLP6 is functioning. I would encourage the authors to at least mention that the protocol used is not optimal, and to consider whether repeating any key experiments using the protocols validated by Sankoh et al. would improve the reliability of these experimental conclusions. Doi: 10.1101/2023.09.30.560305.

As suggested by the reviewer, we replaced the data in Figure 4 with a set of images collected using basic solutions (pH 12). In addition to imaging plasmodesmal callose between epidermal and mesophyll cells, we could detect callose signals in the vasculature of mature leaves. We replaced the data collected from roots with the images collected from leaves. As we mainly focused on the leaf phenotype in this manuscript, the callose accumulation phenotype in leaves makes more biological sense.

To justify the use of near-neutral pH for callose staining in Figures 6B and 7A, we provided the evidence showing that co-localization between aniline blue-stained callose and PDLP5-YFP, which is targeted to PD specifically, is similar between pH 7.4 and 12. Our findings suggest that aniline blue stains callose in basic solutions and near-neutral pH exhibit similar specificity. It is noted that we detected stronger aniline blue signals with near-neutral pH solutions. We did not include the figure in the manuscript.

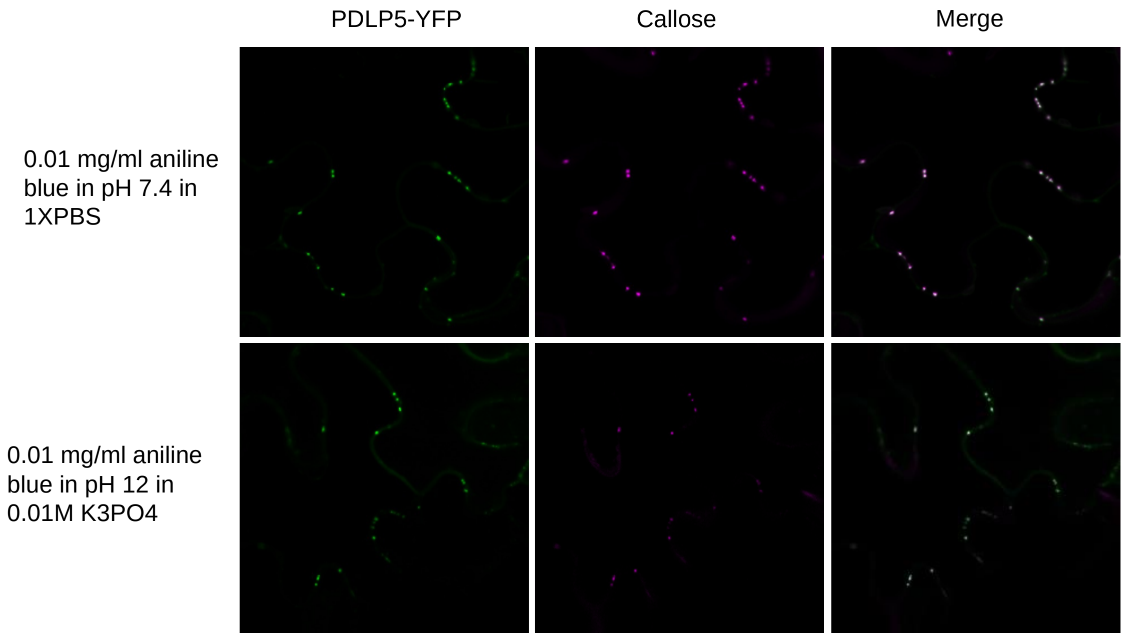

Detection of plasmodesmal callose using aniline blue with near-neutral or basic solutions. PDLP5-YFP fusion protein was expressed in *N. benthamiana* using an Agrobacterium-mediated transiently expression approach. Different aniline blue buffers were infiltrated into the Agrobacterium-infiltrated leaf 10 minutes before imaging.

Incidentally, the data shown in the response to my last review are not encouraging: propidium iodide seems to be staining precisely the same regions as the aniline blue signal! The bright aniline blue-stained "puncta" correspond perfectly to bright puncta of PI staining. This makes me very concerned that the aniline blue stain is nonspecifically binding some other molecule, as expect at the near-neutral pH used.

Looking back at the images, we agree they are not the best representation of plasmodesmal callose in roots. Here, we provided an image of an aniline blue-stained Arabidopsis root (elongation zone) using basic solutions (pH 12). Similar staining patterns were observed using near-neutral solutions (pH 7.4; data not shown). We detected a more typical pattern of plasmodesmal callose at different cell-cell interfaces in Arabidopsis roots, including sieve plates within sieve elements (marked with white arrows). We have yet to optimize a robust staining method for comparing different genotypes. Since we provided callose staining in the leaf vasculatures (Figure 4), we decided not to include the data collected from roots.

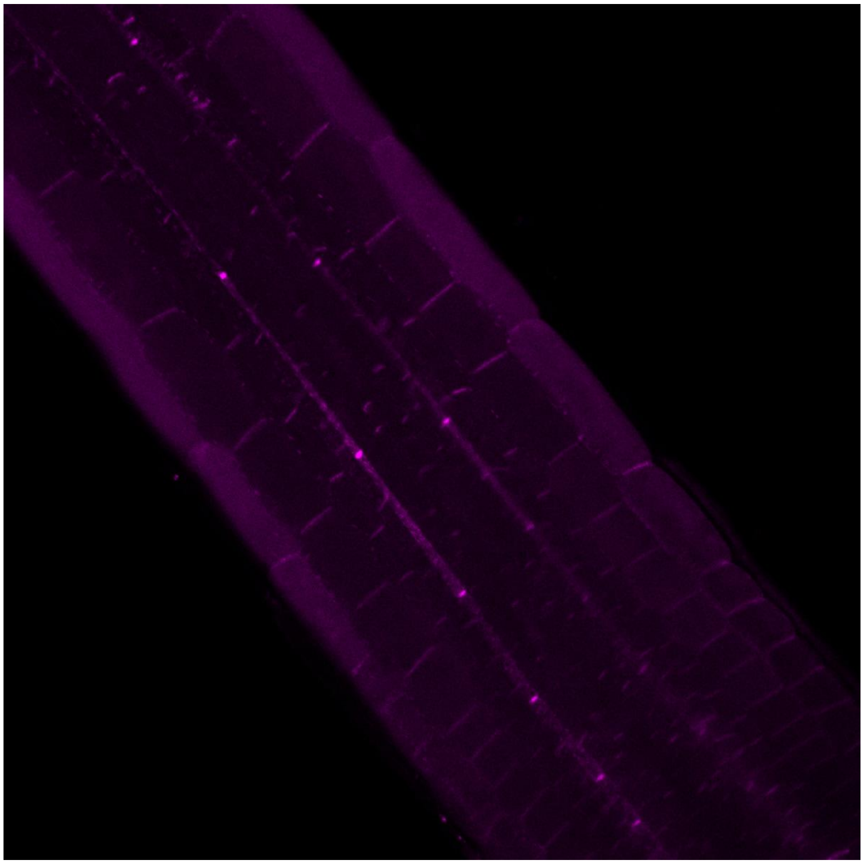

Detection of plasmodesmal callose using aniline blue in an Arabidopsis root. 10-days-old Arabidopsis seedlings were stained with 0.01 mg/ml aniline blue in pH12 for an hour before imaging.

Lastly, one minor suggestion, in the spirit of inclusivity and sensitivity: LL410 and 414, I recommend rephrasing as "...overexpression of PDLP6 delays plant growth" (or "leads to delayed", or "slows") and then as "...demonstrated that the slowed plant growth phenotype" (or "delayed plant growth", etc.).

We edited the phrase as recommended.

Reviewer #2 (Comments for the Author):

The revised version of this manuscript is much improved over the original manuscript. The narrower focus and fewer extraneous claims have streamlined the manuscript and made much clearer the points the authors want to have readers focus on. In particular, the new pull-down assays support the claims of direct physical interaction between PDLP6 and SUS6 (Figure 7). This has certainly improved the manuscript.

Unfortunately, several of the major claims of the manuscript remain unsupported by the data presented. A close examination of Supplemental Dataset 1 and Supplemental Figure 5 reveal that SUS6 was enriched in the MCTP3, PDLP5 and PDLP6 Turbo-ID samples. This contrasts with statements made in the manuscript, lines 317-318. The data presented in Figure 7 is supposed to support the authors' model that a PDLP6-SUS6-CalS7 complex exists in the phloem and there it acts to regulate plasmodesmata by determining callose homeostasis. This complex is proposed to explain the cell type-specific effects observed on overexpression of PDLP5 or PDLP6. But where is the control experiment to support that the interaction with SUS6 is specific to PDLP6? The association of SUS6 with PDLP5, and other plasmodesmal proteins for that matter, cannot be ruled out based on the current data. A pulldown or co-IP with PDLP5 should also be presented to verify the specificity of the PDLP6-SUS6 complex. Further, PDLP6 and PDLP7 have overlapping expression patterns (lines 391-394). Therefore, before claims of a specific or exclusive interaction between PDLP6 and SUS6 can be made, the interaction between PDLP7 and SUS6 should be tested. And why exclude SUS5 from the story if it has a similar expression pattern to SUS6 (line 423)?

We greatly appreciate the valuable feedback provided by the reviewer. We are pleased to note that the reviewer found the manuscript much improved over its original version. In response to the reviewer's comments, we have incorporated the requested co-IP and in vitro pull-down assays involving PDLP5, PDLP7, SUS6, and CalS7. Additionally, we have conducted an in vitro pull-down assay between the PDLPs and CalS1.

The reviewer rightly points out that SUS6 was also significantly enriched by TbID-MCTP3 and PDLP5-TbID. The findings underscore the strengths and limitations of the proximity labeling assay. This assay typically detects proteins within proximity (approximately 20 nm) to the enzyme (TurboID). In most plasmodesmal regions, the plasma membrane and ER membrane are within this range (Nicolas et al., 2017; https://www.nature.com/articles/nplants201782), suggesting that various PD-associated proteins would likely enrich similar proteins functioning in proximity to PD-localized proteins compared to non-PD-associated proteins. As anticipated, PDLP5-TbID, PDLP6-TbID, and TbID-MCTP3 significantly enrich similar proteins targeted to the plasma membrane or ER membrane within PD compared to Col-0 (Supplemental Data Set 1_TbID data sheets). However, we were pleasantly surprised to discover that most proteins targeted to the plasma membrane are significantly enriched by PDLP5-TbID and PDLP6-TbID compared to TbID-MCTP3, whereas TbID-MCTP3 significantly enriches most proteins associated with the ER membrane compared to PDLP5-TbID and PDLP6-TbID. The findings suggest that despite the potential limitations, we can directly compare if different PD-associated proteins significantly enriched different functional partners. Of particular interest for this manuscript was the significant enrichment of SUS6 by PDLP6 compared to PDLP5. To illustrate the differential enrichment of SUS6 by different baits, we have included a heatmap diagram as Supplemental Figure 5I. The enrichment of SUS6 by PDLP5 and MCTP3 gave us more confidence to conclude the plasmodesmal association of SUS6, at least partially. In lines 317-318 of the prior version, we carefully stated that “PDLP6 significantly enriches sucrose synthase 6 (SUS6)” compared to PDLP5. We were careful not to

state that PDLP6 “specifically” enriches SUS6 throughout the manuscript. These findings collectively underscore the efficacy of the proximity labeling assay in identifying proteins functioning in the proximity of PD-associated proteins.

As requested by the reviewer, we presented a co-IP and an in vitro pull-down assay to determine the physical interaction among PDLP5, PDLP7, SUS6, and CalS7. We also tested the interaction between the PDLPs and CalS1. Both methods showed positive interactions of PDLP5-SUS6 and PDLP5-CalS7. We have now included the data in Supplemental Figure 7. The enrichment of SUS6 by PDLP5 in the proximity labeling assay supports the findings that PDLP5 can be present in the same protein complexes with SUS6 (and CalS7) when PDLP5 is ubiquitously expressed. PDLP5 is not predominantly expressed in the vasculatures as SUS6 and CalS7, so it might not be a functional partner of SUS6 and CalS7 under native conditions. In addition, the overexpression of PDLP5 does not lead to callose overaccumulation in the vasculatures. The findings suggest that PDLP5 might not function with SUS6 and CalS7 despite the physical interaction upon overexpression. As we decided to focus on the function of PDLP6 in the vasculatures, we do not include further characterization of PDLP5 overexpressor in this manuscript.

Through the revised manuscript, we were careful not to claim that SUS6 specifically or only interacts with PDLP6. Our proximity labeling, co-IP, and pull-down assays show that PDLP5 can interact with SUS6 when overexpressed in plants or in vitro; however, PDLP5 might not function with SUS6 to regulate plasmodesmal function in the vasculature. Further studies will be required to determine the relationship between PDLP5 and SUS6. With this manuscript, we intended to focus on demonstrating that PDLP6 functions with SUS6 and CalS7 to regulate plasmodesmal function in the vasculature.

As suggested by the reviewer, we also tested the physical interaction between PDLP7, SUS6, and CalS7. We did not detect the presence of PDLP7 in SUS6- or CalS7-containing protein complexes using a co-IP assay (Supplemental Figures 7D-E). PDLP7 could be viewed as a negative control for PDLP6; however, we didn’t make a major claim about it. As PDLP7-CT-biotin binds to a free MBP in an in vitro binding assay (Supplemental Figure 7F), the method is inadequate to determine the direct physical interaction between PDLP7-SUS6, PDLP7-CalS1, and PDLP7-CalS7. Our preliminary data suggest that PDLP7 does not interact with SUS6 and CalS7; however, further analyses will be required to strengthen the conclusion. In addition, PDLP5 and PDLP6 do not interact with CalS1 (Supplemental Figure 7G).

We agreed with the reviewer that including SUS5 in the story could be interesting. Since our proximity labeling assay only identified SUS6, we focused on SUS6 in this manuscript. The suppression of the plant phenotype of *PDLP6-HF* by the *sus6* mutant strongly indicates the pivotal role of SUS6 in PDLP6's function. We will examine the physical and genetic interaction between PDLP6 and SUS5, but we decided not to include it in this manuscript as it is not essential to support our claim.

The manuscript text needs thorough editing and polishing. It is standard practice that results are written in the past tense, and this is not done here. Also by convention, plant protein names are written in capital letters, so 'PLASMODESMATA-LOCATED PROTEIN (PDLP)' and not 'plasmodesmata-located proteins (PDLP)'. Probably due to the extensive revision, the text has become quite choppy and difficulty to follow.

We edited the manuscript as suggested by the reviewer. We moved the description of the justification of the statistical cut-offs of the proximity labeling assay from the main text to the

materials and methods to improve the manuscript's flow further. We also edited the text for a better flow.

As far as is known to date, all PDLPs have one function: to regulate callose homeostasis at plasmodesmata. This leads to them having different physiological roles depending on how they are recruited by the system in question. Lines 75-76 should be more carefully re-stated.

We revised the statement on lines 75-76 in the previous version as the following: “Misexpression of different PDLP members has been demonstrated to impact plant growth, development, and defense.”

We agree with the reviewer that all PDLPs have the same function in regulating callose homeostasis; however, our current data cannot rule out the possibility that different PDLPs have different biochemical activities. As the other two reviewers suggested considering other hypotheses instead of our singular interpretation, we revised a section of our discussion as follows:

“We hypothesize that for PDLP6 to function predominantly in phloem, two key regulations are necessary: (1) transcriptional regulation of *PDLP6* expression in phloem and (2) expression of PDLP6’s functional partners in the same cell types. A previous report has shown that different members of *PDLP* transcripts were detected in distinct Arabidopsis leaf cell types, with PDLP6 identified in phloem parenchyma cells (Kim et al., 2021). We detected PDLP6-YFP fusion protein in phloem, including phloem parenchyma cells, companion cells, and sieve elements (Figures 3C-3G). Conversely, PDLP5 was primarily observed in epidermal cells (Figure 3A-3B). These findings support the transcriptional regulation of PDLPs to express them predominantly in distinct cell types. Using a proximity labeling assay, we identified SUS6 as a potential functional partner of PDLP6 (Figure 6E). Biochemical approaches confirmed the physical interaction between PDLP6 and SUS6 (Figures 7D-7E), and genetic analysis established the dependence of PDLP6 function on SUS6 (Figure 7F). Additionally, we demonstrated physical and genetic interactions between PDLP6 and CalS7 (Figures 7D-7E and 7G). Given that SUS6 and CalS7 are predominantly expressed in the phloem (Barratt et al., 2011; Xie et al., 2011; Yao et al., 2020; Kalmbach et al., 2023), our findings suggest We hypothesize that for PDLP6 to function specifically in phloem.

However, our findings do not rule out the possibility that PDLP5 and PDLP6 are biochemically different. While we detected the physical interaction among PDLP5, SUS6, and CalS7 using co-IP and in vitro pull-down assays (Supplemental Figure 7), the overexpression of PDLP5 does not appear to promote callose accumulation in the vasculature (Figure 4). If the overexpressed PDLP5 can interact with SUS6 and CalS7 in planta, the physical interaction alone may not be sufficient for PDLP5 to function with SUS6 and CalS7 to regulate callose accumulation. Given that PDLP5 is predominantly expressed in non-overlapping cell types compared to SUS6 and CalS7, the observed physical interactions may not hold biological relevance under their native conditions. Additional investigations are necessary to clarify why the overexpression of PDLP5 did not affect plasmodesmal callose accumulation and function in the vasculature.”

Reviewer #3 (Comments for the Author):

In the revised manuscript the authors successfully addressed some of the comments, but provided unclear/speculative responses to others. The authors maintain that the difference between pdlp5 and pdlp6 is due to cell-specific factors. This is overinterpretation and it disregards the over-expression data. The authors show that the Pd localization and the expression levels of the two proteins are very similar in the over-expression lines. Yet, the phenotypes these proteins produce are very different, particularly Pd-callose and starch levels. So, what is the conclusion from these overexpression data? The conclusion is that the two PDLPs are biochemically different. Regardless of the downstream cell-specific factors, these pdlps function differently. In other words, at this point the findings do not explain why overexpression of pdlp6 does not induce callose synthesis in the epidermis and mesophyll like pdlp5 does; and why pdlp5 overexpression does not induce callose in vasculature like pdlp6 does. I can be even more deliberate: for example, why pdlp6 overexpression does not pull down the PIP proteins in the TbID? And so on.

These questions have to be somehow posed and discussed in the manuscript before interpreting what do pdlp5/6 do in their natively expressed tissues.

We greatly appreciate the valuable feedback provided by the reviewer. We are pleased to learn that many of the reviewer's concerns have been successfully addressed. We provided additional information and explanations to address the reviewer's concerns here.

In general, we tried to be very careful with interpreting our results. Our data support the conclusion that PDLP6 functions with SUS6 and CalS7 to regulate the plasmodesmal function in the vasculature. Using PDLP6 as an example, the overexpression of PDLP6-HF requires its functional partners SUS6 and CalS7, impacting plant growth and starch overaccumulation (Figure 7). The genetic interaction studies between *PDLP6*, *SUS6*, and *CalS7* showed that the overexpression of PDLP6 did not lead to delayed plant growth and starch overaccumulation without a functional SUS6 or CalS7 (Figure 7). As suggested by the reviewer, we considered the possibility that "the two PDLPs are biochemically different." Given the unknown biochemical activity of PDLPs, we lack the means to determine whether PDLP5 and PDLP6 are biochemically equivalent. In this revision, we have revised the second and third paragraphs of the discussion as follows, allowing space for interpretation and future exploration:

"We hypothesize that for PDLP6 to function predominantly in phloem, two key regulations are necessary: (1) transcriptional regulation of *PDLP6* expression in phloem and (2) expression of PDLP6's functional partners in the same cell types. A previous report has shown that different members of *PDLP* transcripts were detected in distinct Arabidopsis leaf cell types, with PDLP6 identified in phloem parenchyma cells (Kim et al., 2021). We detected PDLP6-YFP fusion protein in phloem, including phloem parenchyma cells, companion cells, and sieve elements (Figures 3C-3G). Conversely, PDLP5 was primarily observed in epidermal cells (Figure 3A-3B). These findings support the transcriptional regulation of PDLPs to express them predominantly in distinct cell types. Using a proximity labeling assay, we identified SUS6 as a potential functional partner of PDLP6 (Figure 6E). Biochemical approaches confirmed the physical interaction between PDLP6 and SUS6 (Figures 7D-7E), and genetic analysis established the dependence of PDLP6 function on SUS6 (Figure 7F). Additionally, we demonstrated physical and genetic interactions between PDLP6 and CalS7 (Figures 7D-7E and 7G). Given that SUS6 and CalS7 are predominantly expressed in the phloem (Barratt et al., 2011; Xie et al., 2011; Yao et al., 2020; Kalmbach et al., 2023), our findings suggest that PDLP6 functions with SUS6 and CalS7 to regulate plasmodesmal function in the vasculature.

However, our findings do not rule out the possibility that PDLP5 and PDLP6 are biochemically different. While we detected the physical interaction among PDLP5, SUS6, and CalS7 using co-IP and in vitro pull-down assays (Supplemental Figure 7), the overexpression of PDLP5 does not appear to promote callose accumulation in the vasculature (Figure 4). If the overexpressed PDLP5 can interact with SUS6 and CalS7 in planta, the physical interaction alone may not be sufficient for PDLP5 to function with SUS6 and CalS7 to regulate callose accumulation. Given that PDLP5 is predominantly expressed in non-overlapping cell types compared to SUS6 and CalS7, the observed physical interactions may not hold biological relevance under their native conditions. Additional investigations are necessary to clarify why the overexpression of PDLP5 did not affect plasmodesmal callose accumulation and function in the vasculature."

We provided working models to explain the function of PDLP5 and PDLP6 (not included in the manuscript). We hypothesize that for PDLP6 to function, it requires SUS6 and CalS7. Given that the two proteins are only predominantly expressed in the vasculature, the *PDLP6-HF* overexpressor mainly affects the plasmodesmal callose accumulation and function in the same tissue. Physical and genetic interactions support the hypothesis that PDLP6 functions with SUS6 and CalS7 to regulate the plasmodesmal function in the vasculature (Figure 7). Despite the positive physical interaction among PDLP5, SUS6, and CalS7 in our co-IP and in vitro pull-down assays, the PDLP5-HF overexpressor does not lead to the overaccumulation of plasmodesmal callose in the vasculature. We thus hypothesize that PDLP5 functions through the SUS6- and CalS7-independent pathways. In other words, even if PDLP5 is overexpressed, its physical interaction with SUS6 and CalS7 in plants may not be enough to promote plasmodesmal callose accumulation in the vasculature. As none of the SUSs are expressed in the epidermal and mesophyll cells (Yao et al., 2019; https://academic.oup.com/jxb/article/71/6/1858/5660925?login=true), PDLP5 likely functions through the cytosolic invertase (CINV) pathway to regulate callose accumulation in the epidermal and mesophyll cells. Our proximity labeling assay identified PIP5K9 (AT3G09920; Supplemental Data Set 1_TbID data sheets), which physically interacts with CINV1 (Lou et al., 2009; https://academic.oup.com/plcell/article/19/1/163/6091529?login=true), as a functional partner of PDLP5 and PDLP6, but not MCTP3. We are pursuing the potential role of PIP5K9 and cytosolic invertases (CINVs) in regulating callose accumulation in collaboration with PDLP5, which is beyond the scope of this manuscript.

We do not fully understand the reviewer's concern regarding "why pdlp6 overexpression does not pull down the PIP proteins in the TbID?" We are excited to identify PIPs as potential specific functional partners of PDLP5, but not PDLP6. This suggests that despite utilizing a constitutive promoter, the proximity labeling assay significantly enriches a unique set of proteins for PDLP5 and PDLP6. We view these results as a major strength of the experimental system. PDLP5 and PIPs are involved in plant immunity against bacterial pathogens; however, PDLP6 does not seem important in plant immunity (data not shown). The findings suggest that PIPs might function with PDLP5 during immune responses. We are exploring the relationship between PDLP5 and PIPs in regulating plasmodesmata during bacterial infection.

In fig. 7d please indicate "IP-GFP" instead of just "IP". It takes a while to guess what ab was used for IP. The legend also doesn't say what IP was done.

We labeled IP-GFP in the figure and added the information in the figure legend.

In fig. 7E please mark and explain the non-specific bands for MBP-SUS6 and MBP-Cals, especially since these are shorter than the free MBP.

We marked and explained the non-specific bands for MBP-SUS6 and MBP-CalS.

In fig. 7a, the callose images do not match the corresponding gfp images.

Great catch. We swapped the images we displaced. We also cropped and enlarged the images to better display the results.

Lines 276-282: It would be useful to include the data about the phenotype of pUBQ10 transgenic plants used for turbo-ID would in the manuscript, not just in the response letter. The protein levels provided in the supplemental data are not telling us what is the phenotype of those plants.

We included the phenotype of p*UBQ10* transgenic plants used for the proximity labeling assay in Supplemental Figure 5B.

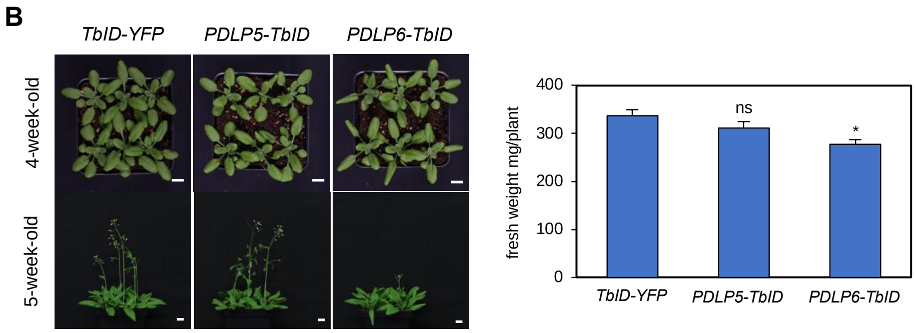

Supplemental Figure 5. B) Growth phenotype of transgenic plants expressing TbID fusion proteins. The plot displays the fresh weight of 4-week-old plants and the mean and standard deviation (n = 14). Asterisks denote statistically significant differences (*t*-Test; two-tailed; P < 0.01), while "ns" indicates no significance.

Lines 399-402: this is speculation based on unrelated evidence.
The movement of the bacterial effector Hopaf1 between cells cannot serve as evidence to suggest movement of the host intrinsic transmembrane Pd proteins like PDLPs. As a putative myristoylated protein, Hopaf1 is predicted to be anchored to the PM, rather than being transmembrane. It could be that these PM-anchored effectors are cleaved at the Pd vicinity before they enter the channel in the soluble form. It is known that by itself myristoylation promotes transient membrane association enabling proteins to anchor to membranes but dissociate easily. More importantly, the effector hopaf1 shown in Li et al (2021) is not enriched at the Pd as the PDLPs are. To the best of my knowledge, no transmembrane Pd proteins have yet been reported to move cell-to-cell.

We removed the statements from the discussion as it is not crucial for this manuscript. We are interested in studying the movement of transmembrane proteins through plasmodesmata by moving through the plasma or ER membranes. We will leave the topic for another time to discuss.

Dear Joe / Dr. Aung:

We have received the reviews of your manuscript titled "PLASMODESMATA-LOCATED PROTEIN 6 regulates plasmodesmal function in Arabidopsis vasculature." Based on the feedback received, the board of reviewing editors would like to accept your manuscript for publication in The Plant Cell. However, this acceptance is subject to minor revisions based on the comments provided by our reviewers. Specifically, we request that you carefully consider the remaining concerns highlighted by Reviewer 1, particularly focusing on avoiding potential conflicting statements in the main text when interpreting the function of PLDP5 and PDLP6, given the complexities of the experimental designs involved.

Please highlight all changes and include a detailed annotation of changes of the text, with line numbers, and noting your responses to the comments.

To submit your revised manuscript, click:

Link Not Available

If you have any questions about the revision submission procedures, please contact the Editorial Office Staff (tpc-submissions@aspb.org). If you cannot return the revised manuscript within 30 days, please let us know. Otherwise, we will assume that you have elected not to revise the manuscript and withdraw it.

Thank you very much for the privilege of reviewing this work. I look forward to receiving the next version.

Sincerely,

The Plant Cell Editorial Board

Reviewer #1 (Comments for the Author):

To begin, thank you to the authors for considering the comments of the several reviewers. As a reviewer, I was especially glad to see that all three reviewers raised similar concerns about the manuscript. Unfortunately, I am not convinced that the revisions have sufficiently addressed the concerns, and I am not sure that the authors fully understood the critiques raised. I will try to briefly address these again, and I encourage the authors to reconsider the various points raised by reviewers, especially the many points of consensus; if three of your colleagues in the field don't follow the logic of the model/hypothesis and how it matches the data presented, then I would encourage the authors to critically evaluate whether sticking to that model/hypothesis is the best course of action.

On PDLP5: I found the new hypothesis raised in the response to reviewers that PDLP5 "may not strongly inhibit plasmodesmal function" alarming, since the evidence for a role of PDLP5 in regulating PD trafficking is much better-established and consistent across different labs than the evidence presented here for PDLP6.

On the many hypotheses: In the response to reviewers, the authors continue to use multiple different hypotheses to explain results. In response to point 1, the authors say that they think PDLP5 and PDLP6 are likely molecularly distinct, with PDLP5 weaker than PDLP6. In response to point 2, they say that PDLP5 and PDLP6 are likely the same in molecular function-but then they say that they have different interacting partners (which would mean that they are molecularly distinct). None of this addresses the claims raised about promoter specificity. The figure offered to reviewers is remarkably speculative.
In other words, as I tried to articulate in the last review-yes, the authors can respond to each of the points individually, but they have not holistically considered whether the data they present support the models they argue for.

Supplemental Figure 7: Having all of these blots cropped and with different contrasts and brightnesses makes it hard for me to fully evaluate, since the effects are subtle. In panel A, I see that some PDLP5-HF coprecipitates with sfGFP, and maybe a little more coprecipitates with SUS6-sfGFP. I guess there is less SUS6-sfGFP than sfGFP, and so the argument is that the ratio of co-IP is different? But it's really not that convincing-certainly not a situation like the cartoon above where PDLP6 is recruiting SUS6 to make callose in a complex!

Supplemental Figure 5B: PDLP6-TbID seems to have a slight growth defect, but nothing like what we saw for the HF-tagged plants. This is with the UBQ10 promoter, so I would have expected even stronger phenotypes, if anything. Does this indicate that the PDLP6-TbID construct is less functional than PDLP6-HF? How does that affect your interpretations?

Reviewer #3 (Comments for the Author):

The manuscript after second revision has significantly improved. After this third round of reviewing, I do not have further comments, as the authors addressed the issues I raised previously. I would only suggest that the title reflects the PDLP5 data as well.

Please also note the following:

-The Plant Cell now requires authors to complete and submit an author revisions checklist upon submission of a revised manuscript. The aim of the checklist is to aid authors in preparing a high-quality manuscript, facilitate the review and assessment of revised manuscripts, and help to ensure that journal standards are maintained across the board. If your manuscript is accepted, the completed checklist will be published as supplemental material attached to the article online. Please download a copy of the checklist (pdf fillable form) at this link, for submission with your revised manuscript: https://tpc.msubmit.net/html/Author_Revisions_Checklist.pdf.

-Supplemental materials should be restricted to large datasets and tables, presentation of replicates, and validation of reagents, methods, or genotypes. Any data that are used to support the major claims must be in the main manuscript. Supplemental figure legends must indicate what figure in the main manuscript is supported by the supplemental data presented. Please justify how each of the supplemental figures meet the criteria.

-Sampling methods and the nature of "biological replicates" should be described precisely (i.e. different plants, parts of plants, pooled tissue, independent pools of tissue, sampled at different times, etc.), along with a clear description of and rationale for any statistical analyses conducted. The reader should know exactly what was sampled; what forms the basis of the calculation of any means and statistical parameters reported. This is also necessary to ensure that proper statistical analysis was conducted.

Reviewer #1 (Comments for the Author):

To begin, thank you to the authors for considering the comments of the several reviewers. As a reviewer, I was especially glad to see that all three reviewers raised similar concerns about the manuscript. Unfortunately, I am not convinced that the revisions have sufficiently addressed the concerns, and I am not sure that the authors fully understood the critiques raised. I will try to briefly address these again, and I encourage the authors to reconsider the various points raised by reviewers, especially the many points of consensus; if three of your colleagues in the field don't follow the logic of the model/hypothesis and how it matches the data presented, then I would encourage the authors to critically evaluate whether sticking to that model/hypothesis is the best course of action.

On PDLP5: I found the new hypothesis raised in the response to reviewers that PDLP5 "may not strongly inhibit plasmodesmal function" alarming, since the evidence for a role of PDLP5 in regulating PD trafficking is much better-established and consistent across different labs than the evidence presented here for PDLP6.

We agreed with the reviewer that "the evidence for a role of PDLP5 in regulating PD trafficking is much better-established and consistent across different labs." We also confirmed and reported similar findings in this manuscript. However, the function of PDLP5 was mostly, if not all, characterized in the epidermal cells. The phenotypes we observed and reported in this manuscript must rely on the function of PDLP5 in mesophyll cells and the vasculature. We hypothesize that the function of PDLP5 in inhibiting the plasmodesmal function in mesophyll cells and vasculature might not be as strong. We acknowledge that proposing something new, slightly new in this case, could be alarming to some readers. Our manuscript provides evidence on the function of PDLP5 and PDLP6 in distinct cell types instead of solely confirming previous findings. Given the complex nature of biology and the evidence provided in the manuscript, we are aware of the possible confusion. We provided thorough explanations to make the case for our major points while allowing readers to interpret our findings.

On the many hypotheses: In the response to reviewers, the authors continue to use multiple different hypotheses to explain results. In response to point 1, the authors say that they think PDLP5 and PDLP6 are likely molecularly distinct, with PDLP5 weaker than PDLP6. In response to point 2, they say that PDLP5 and PDLP6 are likely the same in molecular function-but then they say that they have different interacting partners (which would mean that they are molecularly distinct). None of this addresses the claims raised about promoter

specificity. The figure offered to reviewers is remarkably speculative. In other words, as I tried to articulate in the last review-yes, the authors can respond to each of the points individually, but they have not holistically considered whether the data they present support the models they argue for.

The predominant functionality of PDLP6 in phloem is governed by two key regulatory mechanisms (1) transcriptional regulation of PDLP6 expression in phloem and (2) expression of PDLP6's functional partners in the same cell types, both substantiated by experimental evidence. Firstly, GUS staining results (Figure 3B) indicate the activation of PDLP6 promoter in the vasculature. The detection of PDLP6-YFP fusion proteins driven by the native PDLP6 promoter in phloem, including phloem parenchyma cells, companion cells, and sieve elements (Figures 3C-3G), further supports the transcriptional regulation of PDLP6 expression in phloem. Secondly, the physical interaction between PDLP6 and its functional partners, SUS6 and CalS7, was indicated by co-IP and in vitro pull-down assays (Figure 7D and 7E). Genetic analysis corroborates this interaction by demonstrating PDLP6's dependence on SUS6 and CalS7, which are specifically expressed in the vasculature.

Our observations lead to the hypothesis that both regulatory mechanisms are essential for PDLP6 to predominantly function in phloem. Importantly, these two regulations fit in well each other rather than conflict. The first regulation indicates the transcriptional specificity of PDLP6 expression, while the second elucidates the molecular mechanism at the protein level involved in PDLP6 promoting callose deposition.

In our response to point 1, we aimed to compare and analyze the difference of the phenotypical severity between PDLP5 and PDLP6 overexpression lines. In the model figures presented in our reply to the reviewers' comments, we highlighted the hypothesized protein partners of PDLP6 and PDLP5 involved in regulating callose deposition at plasmodesmata.

The function of PDLP5 and PDLP6 are the same regarding to their role in promoting callose deposition and restricting plasmodesmata-mediated trafficking. However, they would be molecularly distinct in functioning together with different protein partner that are present in the same cell types where PDLP5 and PDLP6 are expressed.

To better address our claims, we revised or added some sentences through the manuscript. For example, the third paragraph of Discussion is revised as followed:

"We consider PDLP5 and PDLP6 functionally equivalent regarding their role in promoting callose accumulation at plasmodesmata. Due to the unknown biochemical activity of PDLPs, we can't determine whether PDLP5 and PDLP6 are biochemically equivalent. However, PDLP5 and PDLP6 could be molecularly distinct, possibly interacting with different functional partners in distinct cell types to exert their function to regulate callose

accumulation. While we detected the physical interaction among PDLP5, SUS6, and CalS7 using co-IP and in vitro pull-down assays (Supplemental Figure 7), the overexpression of PDLP5 does not appear to promote callose accumulation in the vasculature (Figure 4). If the overexpressed PDLP5 can interact with SUS6 and CalS7 in planta, the physical interaction alone may not be sufficient for PDLP5 to function with SUS6 and CalS7 to regulate callose accumulation. Given that PDLP5 is predominantly expressed in non-overlapping cell types compared to SUS6 and CalS7, the observed physical interactions may not hold biological relevance under their native conditions. As none of the SUSs, which can directly convert sucrose into UDP-G for callose synthesis, are expressed in the epidermal and mesophyll cells (Yao et al., 2019), PDLP5 likely relies on the cytosolic invertase (CINV) pathway, involving multiple steps to covert sucrose into UDP-G, to regulate callose accumulation in the epidermal and mesophyll cells."

Supplemental Figure 7: Having all of these blots cropped and with different contrasts and brightnesses makes it hard for me to fully evaluate, since the effects are subtle. In panel A, I see that some PDLP5-HF coprecipitates with sfGFP, and maybe a little more coprecipitates with SUS6-sfGFP. I guess there is less SUS6-sfGFP than sfGFP, and so the argument is that the ratio of co-IP is different? But it's really not that convincing-certainly not a situation like the cartoon above where PDLP6 is recruiting SUS6 to make callose in a complex!

We ensured uniformity in brightness and contrast across images depicting co-immunoprecipitated proteins in both experimental and control samples. Given the significantly more immunoprecipitated sfGFP proteins and minimal co-immunoprecipitated PDLP5 proteins in sfGFP samples, we are confident in the presence of PDLP5-HF and SUS6-sfGFP in the same protein complex when they were transiently expression in *N. benthamiana*.

In addition to co-immunoprecipitation, we conducted in vitro pull-down assays, which unequivocally demonstrated the physical interaction between PDLP6 and SUS6. Combining the co-IP and in vitro pull-down assay results, we concluded that PDLP6 interacts with SUS6 within a protein complex. Moreover, our genetic analysis indicating PDLP6's dependence on SUS6 is consistent with the direct interaction observed between PDLP6 and SUS6.

Supplemental Figure 5B: PDLP6-TbID seems to have a slight growth defect, but nothing like what we saw for the HF-tagged plants. This is with the UBQ10 promoter, so I would have expected even stronger phenotypes, if anything. Does this indicate that the PDLP6-TbID construct is less functional than PDLP6-HF? How does that affect your interpretations?

As shown in Figure 1A-1C, the delayed plant growth phenotype of PDLP6 overexpression lines was determined by the expression level of PDLP6. The growth defects of PDLP6-TbID plants further supported that PDLP6 overexpression affects plant growth. The less severe growth defects of PDLP6-TbID plants may be attributed to relatively lower PDLP6-TbID protein expression levels in these lines. Since we successfully identified many promising candidate proteins from the PDLP6-TbID transgenic lines, the use of the selected transgenic plants does not affect our interpretations in the context of this manuscript. Moreover, we conducted biochemical and genetic interaction assays to confirm the relationship between PDLP6 and a candidate protein SUS6.

Reviewer #3 (Comments for the Author):

The manuscript after second revision has significantly improved. After this third round of reviewing, I do not have further comments, as the authors addressed the issues I raised previously. I would only suggest that the title reflects the PDLP5 data as well.

We appreciate the reviewer's thorough assessment of our manuscript and are pleased to have addressed all raised concerns. Regarding the title, we maintain our preference for "PLASMODESMATA-LOCATED PROTEIN 6 regulates plasmodesmal function in Arabidopsis vasculature" as it succinctly encapsulates the primary findings of our study. Our manuscript focuses on illustrating the role of PDLP6 in regulating plasmodesmal function, from initial functional characterization through overexpression analyses to the elucidation of molecular mechanisms via identification of protein partners. The differences between PDLP5 and PDLP6 in terms of expression patterns and molecular mechanisms governing callose accumulation represents a significant aspect of our work, underscoring the distinct functions of these two proteins in different cellular contexts. However, the molecular mechanisms including the protein partners of PDLP5 in regulating plasmodesmal function were not characterized in our manuscript. Together, we decided to highlight solely on PDLP6 in the title to maintain clarity and relevance to the main scope of our study without reflecting PDLP5 data in the title.

Dear Dr. Kyaw Aung:

We are pleased to inform you that your paper entitled "PLASMODESMATA-LOCATED PROTEIN 6 regulates plasmodesmal function in Arabidopsis vasculature" has been accepted for publication in The Plant Cell, pending a final editorial review by a science editor. At this stage, your manuscript will be evaluated by a science editor with respect to its presentation of scientific content, compliance with journal policies, and presentation for a broad readership. The Plant Cell has contracted with Plant Editors (planteditors.com) to provide this service to our authors, and you will soon receive additional information on this process.

Please note that each author needs to link their ORCID identifier to their account in the system before your manuscript can be published. If any authors do not have an ORCID linked to their account, they will receive a message with a link to complete this task. Please ensure that ALL of your coauthors have completed this task as soon as possible.

ASPB offers an OPEN option that allows authors to have their online articles available for free to all users immediately upon publication. For more information about the ASPB OPEN option, refer to the Final Submission Checklist Form.

The Plant Cell and The Arabidopsis Information Resource (TAIR) are collaborating to collect functional annotation data about Arabidopsis genes from authors. This includes information about the gene's molecular function (e.g., kinase activity, ATP synthetase activity), the biological process/es it is involved in (e.g., endosperm development, threonine biosynthesis), its subcellular location (e.g., nucleus, ER), anatomical or developmental expression pattern (e.g., leaf, ovule, flower stage 10, seedling stage), or its partner in a protein-protein interaction (e.g., AT1G01010 interacts with AT1G01020).

If your paper contains results falling into one or more of these categories for Arabidopsis genes, we request that you now submit these data for inclusion in TAIR by filling in the form provided at the following URL: https://www.arabidopsis.org/doc/submit/functional_annotation/123. If you need further clarification on what types of data can be submitted please contact curator@arabidopsis.org.

Finally, we encourage your submission of artwork for the journal cover. Monthly issues will have an online cover image and selected covers will be used for posters, other promotional items, and "wallpaper" for mobile devices. For more information, refer to Cover Submission in the Instructions for Authors https://tpc.msubmit.net/cgi-bin/main.plex?form_type=display_auth_instructions.

We look forward to seeing your paper published.

Sincerely,

The Plant Cell Editorial Board

=====
IMPORTANT REMINDER: PEER REVIEW REPORTS
=====

If you opted to publish a peer review report along with your article during the original submission process, it will be prepared by the editorial staff and publicly posted with your manuscript, inside the zip file that contains any other supplemental material. As a reminder, the peer review report is a public record of all comments from editors and reviewers, as well as your prior responses, as you received them in the decision letters for each draft of your manuscript. If you agreed to publish this report and have changed your mind, or are not sure if you selected this option, please contact the editorial office as soon as possible before signing the license agreement from our publisher.

----FOR ASPB OFFICE USE ONLY (DO NOT EDIT)----
MSID: 35679
Corresponding Author: Dr. Aung
