# Supplementary material for: PLASMODESMATA-LOCATED PROTEIN 6 regulates plasmodesmal function in Arabidopsis vasculature
Source: Plant Cell. 2024 Jun 6;36(9):3543–61. doi: 10.1093/plcell/koae166 (PMC11371196; doi:10.1093/plcell/koae166)
Supplement: koae166_Supplementary_Data [file koae166_supplementary_data.zip › tpc.23.00745Supplementary Figures and tables.pdf]

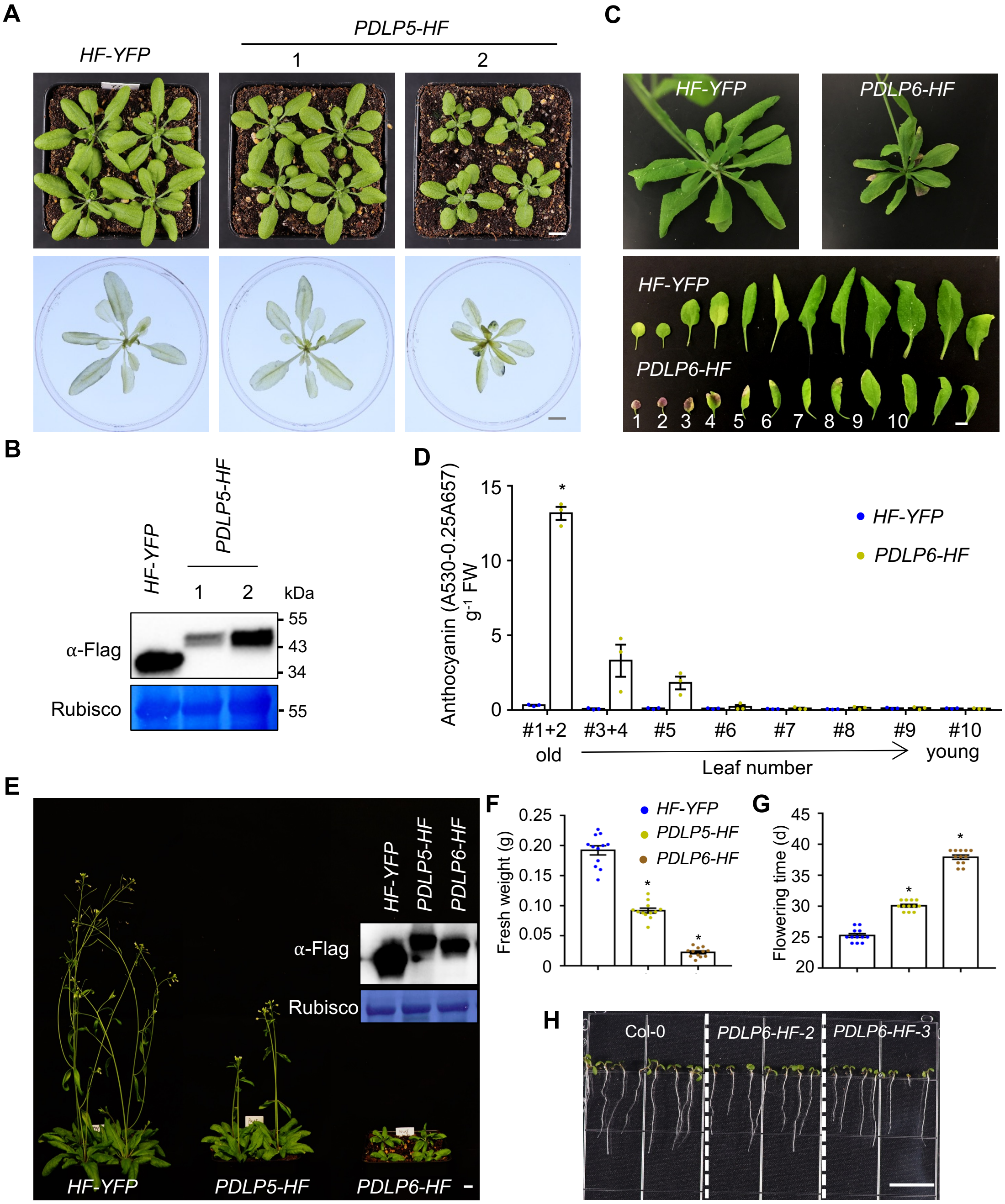

Supplementary Figure S1. Growth phenotypes of *PDLP5-HF* and *PDLP6-HF* (Supports Figure 1 and 2). **A**) The overexpression of PDLP5 leads to stunted plant growth (top panel). HF-YFP serves as a control. Images were taken from 25-day-old plants growing under a standard light condition (110 µmol m-2 s-1) using the same magnification. Tissues were harvested at the end of the night and stained using Lugol's iodine solution (lower panel). Scale bar = 1 cm. **B**) Immunoblot analysis detects the expression of PDLP5-HF in the two transgenic lines. An anti-Flag antibody was used to detect the expression of Flag-fusion proteins. Rubisco serves as a loading control. Numbers on the side indicate molecular weights in kilodaltons (kDa). **C**) Older leaves of *PDLP6-HF-2* exhibit a purple color. Scale bar = 1 cm. **D**) Anthocyanin accumulation in leaves shown in **C**). Anthocyanins were extracted from different leaves (old to young) and measured at absorbance 530 nm and 657 nm using a microplate reader. Leaves from two plants were combined to form a single replicate. The plot shows the mean with SEM (n = 3). Asterisks indicate statistically significant differences analyzed with a two-paired *t test* (*, *P* < 0.01). **E**) Late-flowering phenotypes of *PDLP5-HF* and *PDLP6-HF*. *HF-YFP* serves as a control. Images were taken from 35-day-old plants using the same magnification. An anti-Flag antibody was used to detect the expression of PDLP5-HF and PDLP6-HF. Rubisco serves as a loading control. Scale bar = 1 cm. **F**) Quantification of plant growth by fresh weight. The above-ground tissues of 25-day-old plants were quantified. The plot shows the mean with SEM (n = 12). Asterisks indicate statistically significant differences (*t*-Test; two-paired; *P* < 0.01). **G**) Quantification of flowering time. The flowering time of *PDLP5-HF*, *PDLP6-HF*, and *HF-YFP* was measured as the number of days at bolting. The plot shows the mean with SEM (n = 12). Asterisks indicate statistically significant differences analyzed with a two-paired *t* test (*, *P* < 0.01). **H**) 7-day-old Seedlings of Col-0, *PDL6-HF-2*, and *PDLP6-HF-3* grown on ½ LS (+1% sucrose) were photographed. Scale bar = 1 cm.

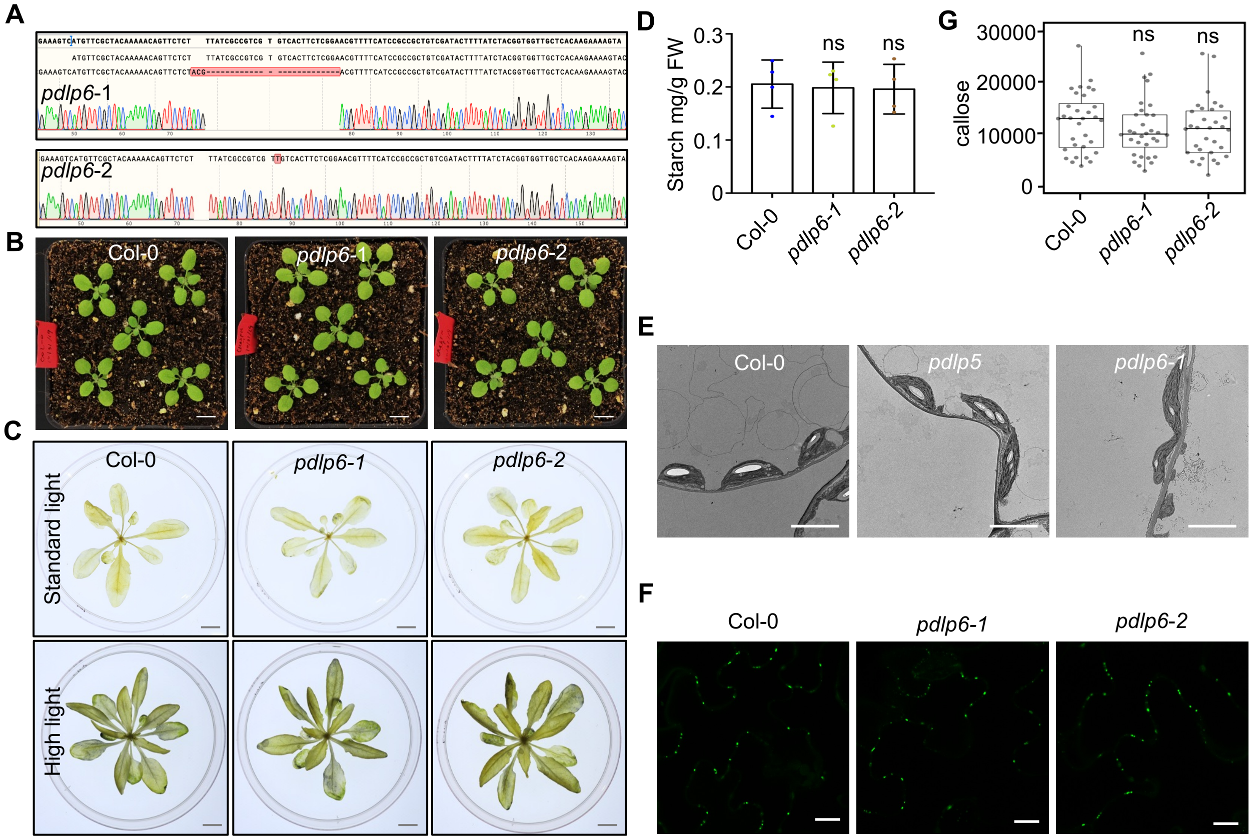

Supplementary Figure S2. Characterization of *pdlp6* mutants (Supports Figure 1). **A**) Genotyping of *pdlp6-1* and *pdlp6-2* mutants generated by CRISPR/Cas9 technology. *pdlp6-1* and *pdlp6-2* carry 26 bp deletion and a G to T mutation around the gRNA target sites, respectively. **B**) 2-week-old Arabidopsis plants were grown under a standard Arabidopsis growth condition. Images were taken using the same magnification. Scale bars = 1 cm. **C**) Starch staining of *pdlp6* mutants. Tissues were harvested at the end of the night and stained using Lugol's iodine solution. Images were taken using the same magnification. Scale bars = 1 cm. **D**) Quantification of starch in leaves of high-light treated plants at the end of the night. Mature leaves from three plants were collected and combined to form a single replicate. The plot shows the mean with SD (n = 4). ns: no significance (Mann-Whitney *U* Test; two-paired; $P < 0.05$). **E**) TEM images of chloroplasts from mesophyll cells of Col-0, *pdlp5*, and *pdlp6-1* mutants. Samples for TEM were collected from 5-week-old high-light treated plants. **F**) Callose accumulation between leaf epidermal cells. Scale bars = 10 µm. **G**) Quantification of callose shown in **F**). Each dot represents the total signal intensity from one image. The box plots show the mean with SD. n=32. Images were captured from six leaves, with two obtained from each plant. ns: no significance (Mann-Whitney *U* Test; two-paired; $P < 0.05$).

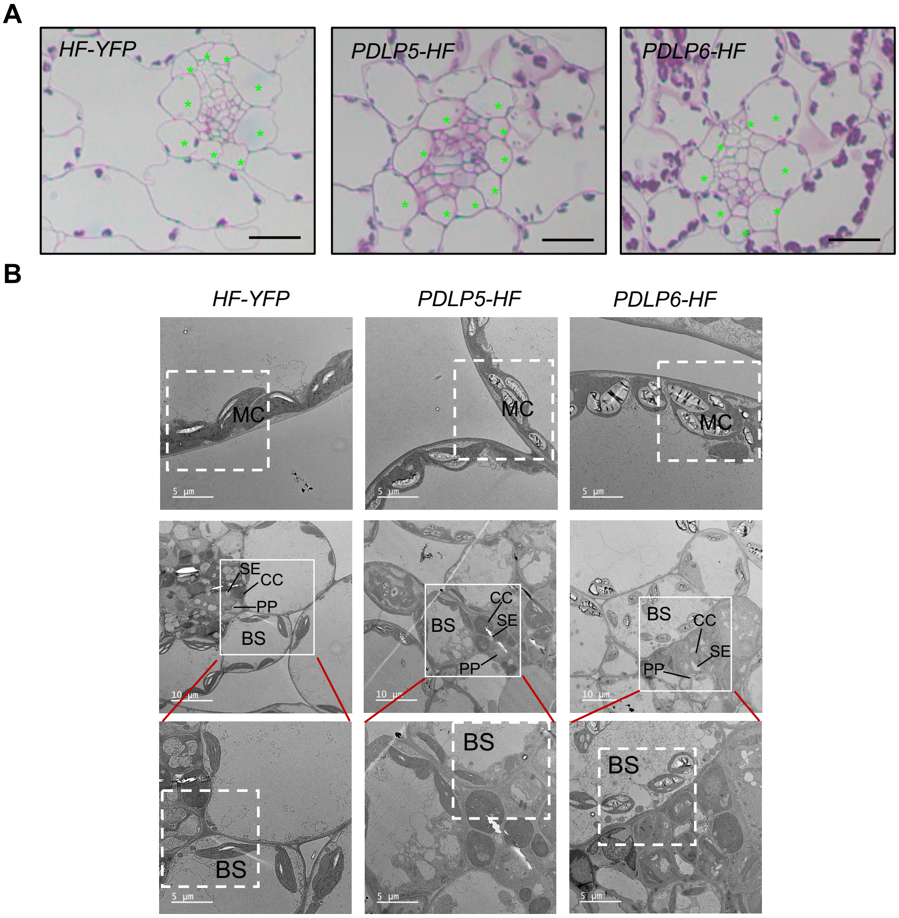

Supplementary Figure S3. Starch accumulation in mature leaves of different Arabidopsis genotypes (Supports Figure 2). **A**) Histological sections of Arabidopsis leaves. Mature leaves of 5-week-old high light-treated plants were subjected to sectioning and staining with periodic acid/Schiff reagent, which stains polysaccharides in the cell wall and starch grains in chloroplasts. Asterisks mark bundle sheath cells. Scale bars = 20 μm. **B**) TEM images show the starch granules in chloroplasts in different cells of mature leaves. White dashed frames indicate the images in **Figure 2F**. MC: mesophyll cell; BS: bundle sheath cell; PP: phloem parenchyma cells; CC: companion cells; and SE: sieve elements. Scale bars = 5 μm or 10 μm.

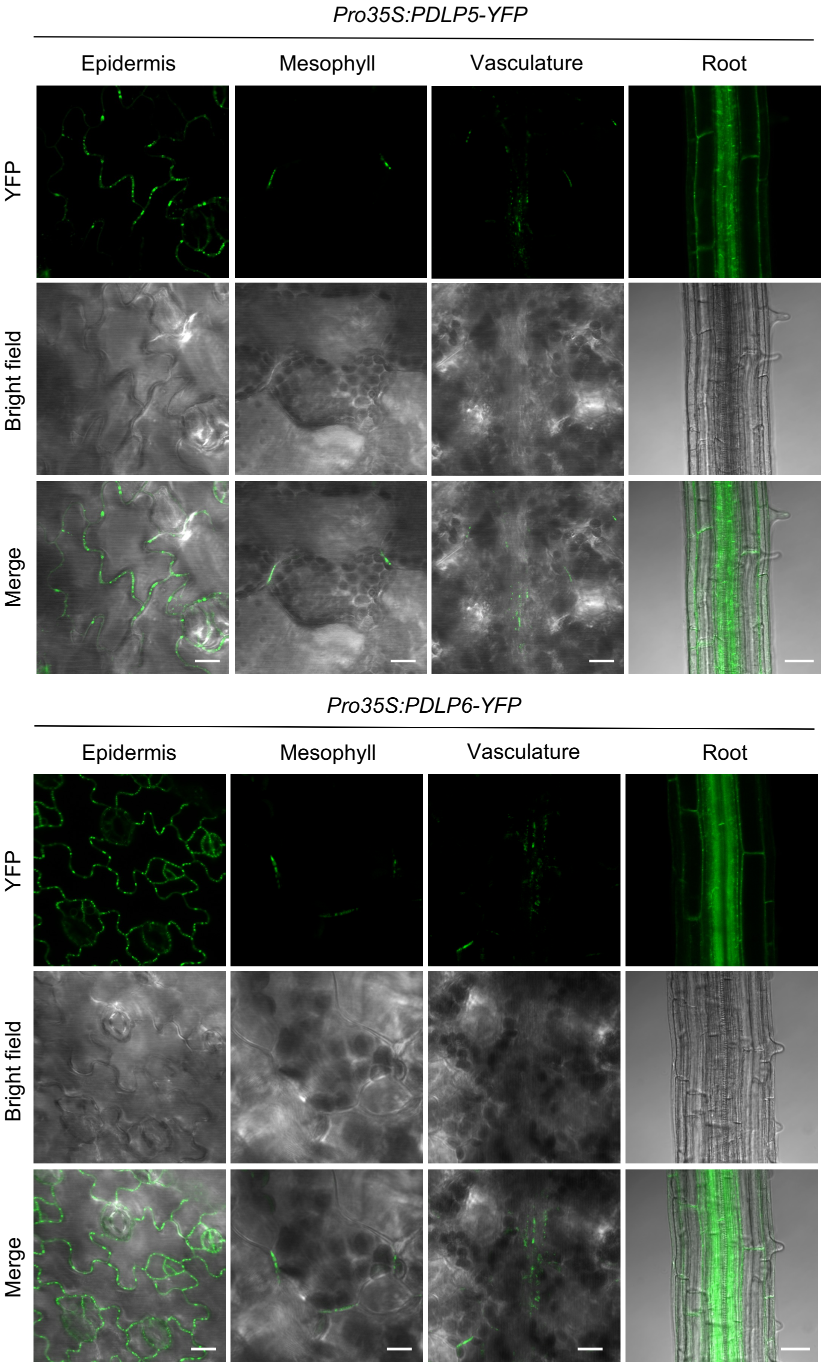

Supplementary Figure S4. Ubiquitous expression of PDLP5-YFP and PDLP6-YFP fusion proteins in *Pro35S:PDLP5-YFP* and *Pro35S:PDLP6-YFP* transgenic plants (Supports Figure 3 and 4). The expression of the fusion proteins was detected in epidermal cells, mesophyll cells, vasculatures in leaves, and most cell types in roots. Confocal images were captured from 2-week-old Arabidopsis seedlings. Green signals represent the expression of YFP fusion proteins. Scale bars for epidermis, mesophyll, and vasculature = 10 μm. Scale bars for root = 50 μm.

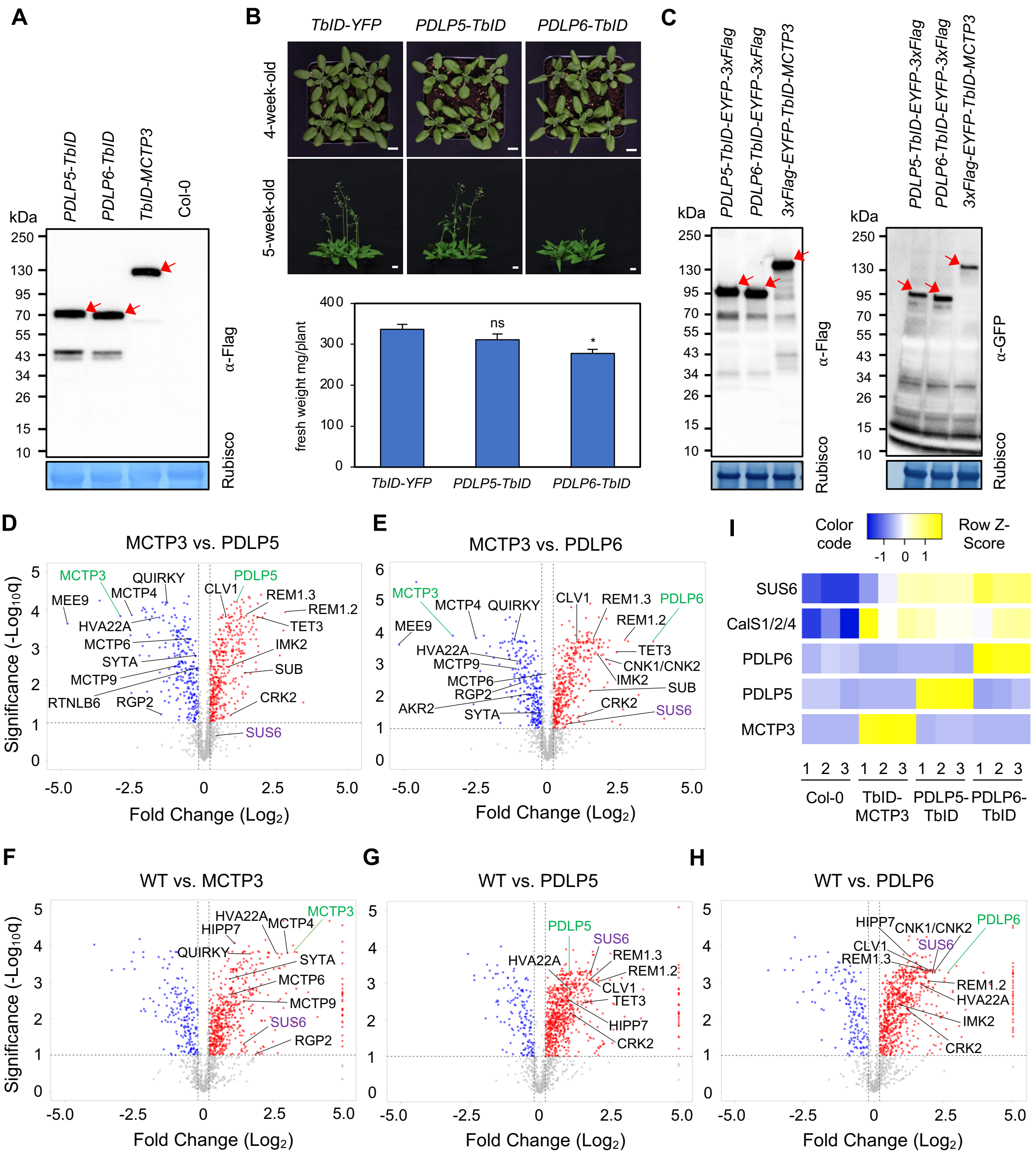

Supplementary Figure S5. The expression of PDLP5-TbID, PDLP6-TbID, and TbID-MCTP3 and their enriched proteins (Supports Figure 6). **A**) Immunoblot analysis shows the expression of PDLP5-TbID, PDLP6-TbID, and TbID-MCTP3. 2-week-old seedlings of wild-type Col-0 and Arabidopsis transgenic plants were subjected to immunoblot analysis using an anti-Flag antibody. Rubisco serves as a loading control. Red arrows indicate the TbID fusion proteins. Numbers on the side indicate molecular weights in kilodaltons (kDa). **B)** Growth phenotype of transgenic plants expressing TbID fusion proteins. The plot displays the fresh weight of 4-week-old plants and the mean and standard deviation (n = 14). Asterisks denote statistically significant differences (*t*-Test; two-tailed; P < 0.01), while "ns" indicates no significance. **C**) Immunoblot analysis shows the expression of PDLP5-TbID-EYFP, PDLP6-TbID-EYFP, and EYFP-TbID-MCTP3. Agrobacteria harboring *ProUBQ10:PDLP5-TurboID-EYFP-3xFlag* (PDLP5-TbID-EYFP-3xFlag), *ProUBQ10:PDLP6-TurboID-EYFP-3xFlag* (PDLP6-TbID-EYFP-3xFlag), and *ProUBQ10:3xFlag-EYFP-TurboID-MCTP3* (3xFlag-EYFP-TbID-MCTP3) were infiltrated into *N. benthamiana* to transiently overexpress the EYFP fusion proteins. Anti-Flag and anti-GFP antibodies were used. Rubisco serves as a loading control. Numbers on the side indicate molecular weights in kilodaltons (kDa). **D-H**) Volcano plots show significantly enriched proteins in MCTP3, PDLP5, and PDLP6 samples. Candidates were filtered using cutoffs $log_2FC > 0.2$ or < -0.2 and $q < 0.1$. Plots were generated using VolcaNoseR. A few of the known and putative PD-associated proteins are labeled. **I**) A heatmap diagram illustrates the differential enrichment of SUS6 and CalS1 by PDLPs and MCTP3. Each protein is color-coded to represent the extent of enrichment, indicated as z-scores. The samples, denoted by numbers, are displayed in distinct columns corresponding to different genotypes.

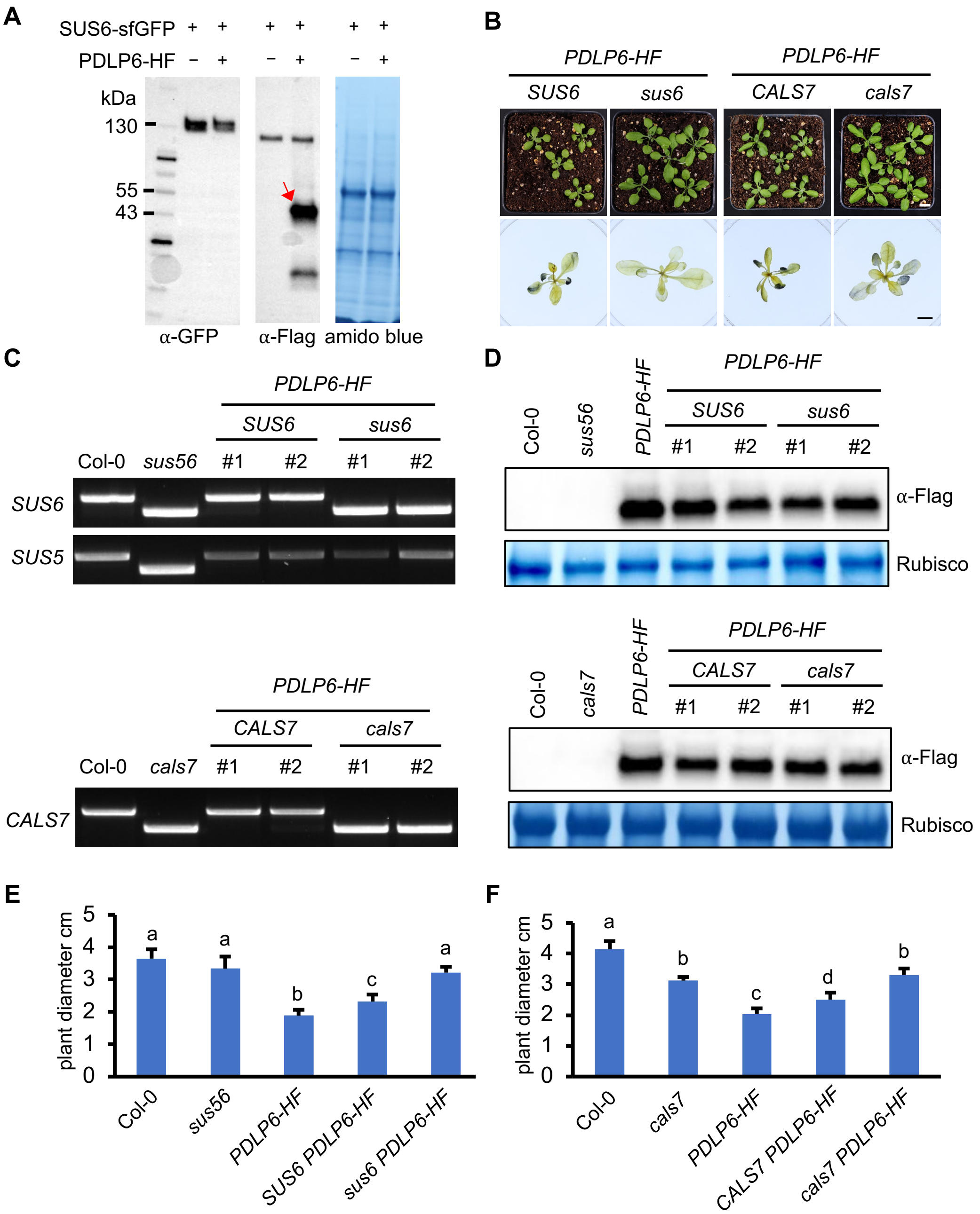

Supplementary Figure S6. Determination of genetic interactions among PDLP6, SUS6, and CALS7 (Supports Figure 7). **A)** Immunoblot analysis detects the expression of SUS6-sfGFP and PDLP6-HF transiently expressed in *N. benthamiana*. Anti-Flag and anti-GFP antibodies were used to detect the expression of Flag- and GFP-fusion proteins, respectively. Red arrow indicates PDLP6-HF proteins. Rubisco serves as a loading control. Numbers on the side indicate molecular weights in kilodaltons. **B**) Independent F_3 progenies of *sus56* crossing *PDLP6-HF-2* and *cals7* crossing *PDLP6-HF-2 are* shown in **Fig. 6**. Images were taken using the same magnification. Scale bars = 1 cm. Plants were collected at the end of the night and subjected to starch staining. **C**) PCR Genotyping analysis. A pair of primers (LP and RP) was used to amplify genomic DNA and another pair of primers (SALK LBb1.3 and RP) was used to amplify the presence of T-DNA for each genotype. Upper bands indicate the PCR products of genomic amplifications. The lower bands indicate the presence of T-DNA insertions in both chromosomes (homozygous lines). **D**) Immunoblot analysis detects the expression of PDLP6-HF. An anti-Flag antibody was used to detect the expression of Flag-fusion proteins. Rubisco serves as a loading control. **E-F)** Quantitative analysis of plant growth of progenies of the genetic crosses of *sus56* with PDLP6-HF-2 and *cals7* with PDLP6-HF-2. The diameter of plants shown in Figures 7F and 7G was measured. The plots show the mean with SD (n = 6). Different letters on the bar indicate statistically significant differences analyzed with one-way ANOVA ($P < 0.0001$).

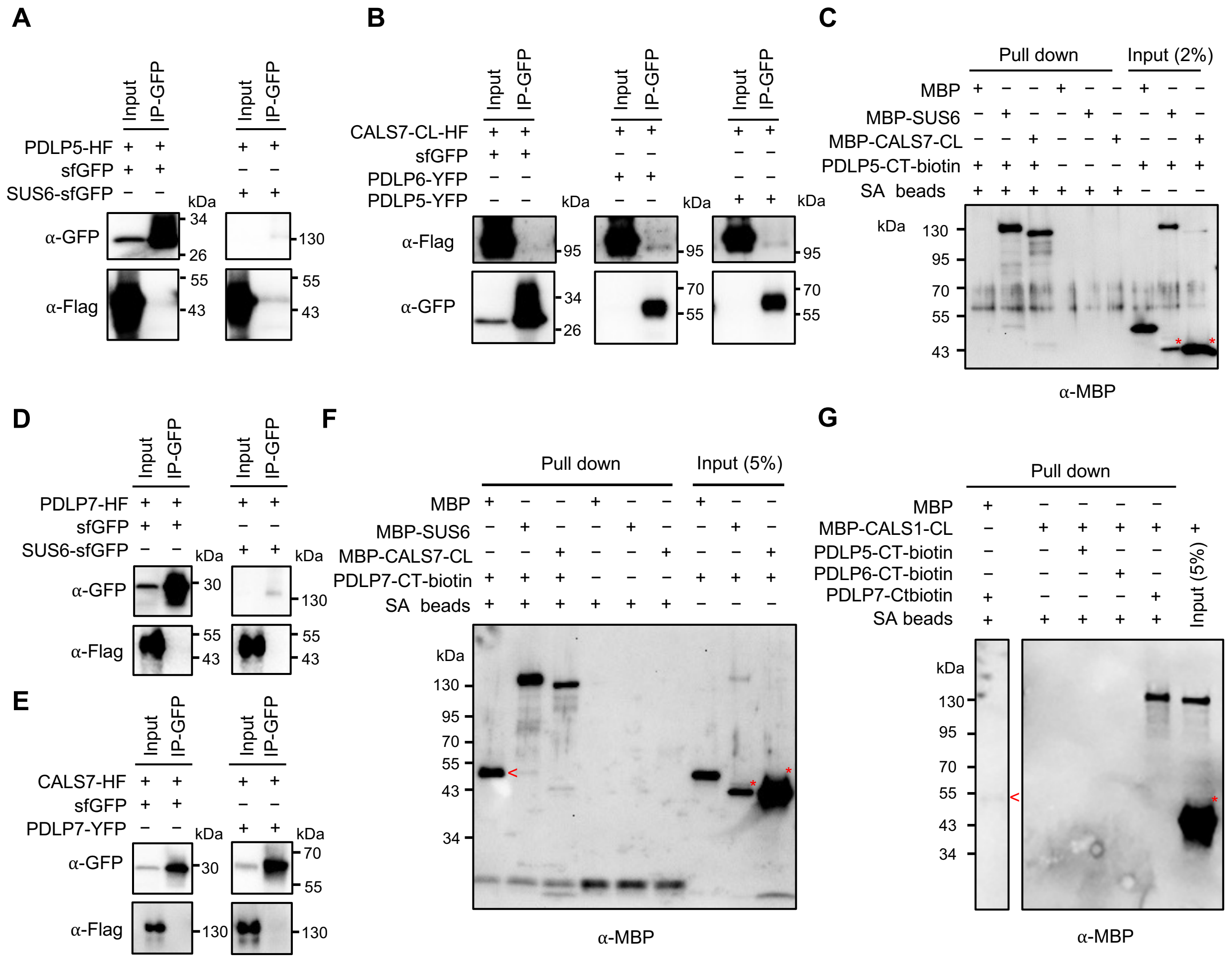

Supplementary Figure S7. Interaction among PDLPs, SUS6, and CALSs (supports Figure 7). **A-B**) Co-IP assays detect the presence of PDLP5-SUS6 and PDLP5-CALS7 protein complexes. Agrobacteria harboring different plasmids were co-infiltrated into *N. benthamiana*. Samples were collected 2 days after infiltration and subjected to Co-IP. A GFP antibody was used to detect the expression of sfGFP or YFP fusion protein and the enrichment of sfGFP or YFP fusion proteins by GFP-Trap. A Flag antibody was used to detect the expression of PDLP5-HF fusion protein and the interaction between HF and sfGFP fusion proteins. A free sfGFP fusion protein is included as a negative control. Numbers on the side indicate molecular weights in kilodaltons (kDa). **C**) In vitro pull-down assay detects the direct interaction between PDLP5-SUS6, and PDLP5-CALS7. The biotinylated C-terminal tail of PDLP5 (PDLP5-CT-biotin) was incubated with recombinant proteins, MBP, MBP-SUS6, or MBP-CALS7. Magnetic beads coupled with streptavidin (SA beads) were used to pull down PDLP5-CT-biotin and the interacting proteins. A MBP antibody was used to detect the interaction between PDLP5 and MBP fusion proteins. Asterisks indicated non-specific bands for MBP-SUS6 and MBP-CALS7-CL. Numbers on the side indicate molecular weights in kilodaltons (kDa). **D-E**) Co-IP assays detect the presence of PDLP7-SUS6 and PDLP7-PDLP7 protein complexes. The co-IP assay was conducted as mentioned above. **F**) In vitro pull-down assay detects the direct interaction between PDLP7-SUS6 and PDLP7-CALS7. The in vitro pull-down assay was conducted as mentioned above. An arrowhead indicated the binding of PDLP7-CT-biotin with a free MBP. Asterisks indicated non-specific bands for MBP-SUS6 and MBP-CALS7-CL. **G**) In vitro pull-down assay shows no direct interaction between PDLP5-CALS1 and PDLP6-CALS1. The in vitro pull-down assay was conducted as mentioned above. An arrow indicated the binding of PDLP7-CT-biotin with a free MBP. Asterisks indicated non-specific bands for MBP-SUS6 and MBP-CALS7-CL.

Supplementary Table S1. Primers used for mutant genotyping in this study.

Primer	Sequence	For genotyping
SALK_048921_LP	TGGCAAGAATAGATCCTGACG	*cals7*
SALK_048921_RP	ATGGATGGTTTTCCTATTGGC	
SALK_152944_LP	ATTTCCCTTTTACCGCACAAG	*sus5*
SALK_152944_RP	TTGTTTGGCCAGTTTCTGATC	
SALK_107491_LP	TGACACGGTTAATACCGGAAG	*sus6*
SALK_107491_RP	ATCCATCTGAATTTCCCCTTG	
LBb1.3	ATTTTGCCGATTTCGGAAC	SALK mutants
LB2	ATCTTCCCAAATTACCAATACA	SAIL mutants
CRISPR-PDLP6-F	AGTCGCGACGTTCTCATTCT	CRISPR lines
CRISPR-PDLP6-R	CGAACCACCACCGTCTTATC	

Supplementary Table S2. Primers used for plasmid construction in this study.

Primer	Sequence	For cloning
attB1-PDLP5-F	ggggacaagtttgtacaaaaaagcaggcttcATGATCAAGACAAAGACGACG	*Pro35S:PDLP5-HF, ProUBQ10:PDLP5-TbID-3xFlag, and Pro35S:PDLP5-YFP*
attB2-PDLP5-R	ggggaccactttgtacaagaaagctgggtcTTTACACCATTTCTCATCTTG	
attB1-PDLP6-F	ggggacaagtttgtacaaaaaagcaggcttcATGTTCGCTACAAAAACAGTTC	*Pro35S:PDLP6-HF, ProUBQ10:PDLP6-TbID-3xFlag, and Pro35S:PDLP6-YFP*
attB2-PDLP6-R	ggggaccactttgtacaagaaagctgggtcCTTCCCACCTTTACCTCTCTC	
attB1-PDLP7-F	ggggacaagtttgtacaaaaaagcaggcttcATGCCAATGGCAAAACTAAGG	*Pro35S:PDLP7-YFP*
attB2-PDLP7-R	ggggaccactttgtacaagaaagctgggtcAATAAGAGTAAAACTATGTAAGATAG	
attB1-MCTP3-F	ggggacaagtttgtacaaaaaagcaggcttcATGCAGAGACCACCTCCTG	*ProUBQ10:TbID-3xFlag-MCTP3*
attB2-MCTP3-R	ggggaccactttgtacaagaaagctgggtcTCAGAGCATGCAATCAGTTCTTG	
attB1-EYFP-F	ggggacaagtttgtacaaaaaagcaggcttcATGGTGAGCAAGGGCG	*Pro35S:HF-YFP*
attB2-EYFP-R	ggggaccactttgtacaagaaagctgggtcTTACTTGTACAGCTCGTCC	
CRISPR-PDLP6	acagctagagtcgaagtagtgattgCGTTCCGAGAAGTGACACGAgttttagagctagaaatagcaagtt	*pKIR-CRISPR-PDLP6*
attB1-PDLP5-Pro-F	ggggacaagtttgtacaaaaaagcaggcttcctaaatgattacggatccac	*ProPDLP5:PDLP5-YFP and ProPDLP5:GUS*
attB2-PDLP5-ORF-R	ggggaccactttgtacaagaaagctgggtcTTTACACCATTTCTCATctgC	
attB1-PDLP6-Pro-F	ggggacaagtttgtacaaaaaagcaggcttcgttgagcctctcttgctaatg	*ProPDLP6:PDLP6-YFP, ProPDLP6:GUS and ProPDLP6:1xYFP*
attB2-PDLP6-ORF-R	ggggaccactttgtacaagaaagctgggtcCTTCCCACctgatatattaaaaac	*ProPDLP6:PDLP6-YFP*
attB2-PDLP5-Pro-R	ggggaccactttgtacaagaaagctgggtcggttactttttgttttgagag	*ProPDLP5:GUS*
attB2-PDLP6-Pro-R	ggggaccactttgtacaagaaagctgggtcgactttcgacgattgcttc	*ProPDLP6:GUS, ProPDLP6:1xYFP*
YFP-3'UTR-F	gacgagctgtacaaggcttagagctcATAACGTTATGGGACTTTTGACT	*ProPDLP6:PDLP6-YFP-3'UTR-Ter*
3'UTR-Ter-R	ttgaacgatcggggaaattcgagctcTCGATGTTGAGTCTAACTATATTCTTTCA	
GW-HiFi-F	GGAAACAGCTATGACCATGATTACGcctgcaggtcgactctagag	*ProPDLP6:1xYFP*
EYFP-HiFi-R	CGAACGAAAGCTCTGCAGGTCGACTgagctctaCCTAGGagccttgtacagctcgtcc	
pUBQ10_3'end-F	gattaacagaagggcgaattcgac	*ProUBQ10:PDLP5-TbID-EYFP-3xFlag, ProUBQ10:PDLP6-TbID-EYFP-3xFlag*
TbID-linker-R	ccgccgcttccaccgcctccCTGCAGCTTTTCGGCAGAC	
Linker-F	ggaggcggtggaagcgg	
EYFP- FLAG-R	atcaccgtcatggtctttgtagtccCTtctagaCTTGTACAGCTCGTCCATG	
EYFP -FLAG-R	atcaccgtcatggtctttgtagtccCTactagtCTTGTACAGCTCGTCCATGC	
FLAG-EYFP-F	gattacaaggatgacgatgacaagctcgagATGGTGAGCAAGGGC	*ProUBQ10:3xFlag-EYFP-TbID-MCTP3*
EYFP-L-R	tccgccgcttccaccgcctccCTTGTACAGCTCGTCCATG	
L-TbID-F	gtggaagcggcggaggttccGCTAGCAAAGACAATACTGTGC	
TbID-attR-R	aGCTTTTTTGTACAAACTTGTgatctcgagCTGCAGCTTTTCGGCAGACC	
attB1-SWEET13-F	ggggacaagtttgtacaaaaaagcaggcttccaatttgtgctttaagtagtgttc	*ProSWEET13:SWEET13-mCherry*
attB2-SWEET13-R	ggggaccactttgtacaagaaagctgggtcAACTTGACTTTGTTTCTGGAC	
attB1-SERO2-F	ggggacaagtttgtacaaaaaagcaggcttccacgcacataaaaaaaagtgtataag	*ProSEOR2:SEOR2-mCherry*
attB2-SEOR2-R	GGGGACCACTTTGTACAAGAAAGCTGGGTCctcaaggcagcattggtaca	
proSUC2-F	cctctatctttacgatctaggaaggaagttcgaagatagttaattatttgggggatatttgaattattaatgt	

Primer	Sequence	For cloning
proSUC2-R	cgttcagcttttttgtacaaacttgtgatctcgagatttgacaaaccaagaaagtaagaaaaaaaag	*ProSUC2:PP2A1 -mCherry*
attB1-PP2A1-F	GGGGACAAGTTTGTACAAAAAAGCAGGCTTCatgtttagaaaccaagactcgaaatact	
attB2-PP2A1-R	GGGGACCACTTTGTACAAGAAAGCTGGGTCctgtttgggacgaattgcaac	
attB1-SUS6-F	ggggacaagtttgtacaaaaaagcaggcttcATGTCATCTTCATCTCAAGC	*Pro35S:SUS6-sfGFP, Pro35S:SUS6-HF*
attB2-SUS6-R	ggggaccactttgtacaagaaagctgggtcATACTCTTGAGCCGAGTTAG	
MBP-SUS6-F	aacctgtacttccagggtcatatgATGTCATCTTCATCTCAAGCTATG	*pET-MBP-SUS6*
MBP-SUS6-R	actggcggccgttactagtggatccTTAATACTCTTGAGCCGAGTTAGC	
MBP-CalS1-CL-F	aacctgtacttccagggtcatatgGAGATCCGAACACTTGGAA	*pET-MBP-CalS1-CL*
MBP-CalS1-CL-R	actggcggccgttactagtggatccTCAATAACAAGACAGCATCCG	
MBP-CalS7-CL-F	aacctgtacttccagggtcatatgATACGCACACTTGGAATGT	*pET-MBP-CalS7-CL*
MBP-CalS7-CL-R	actggcggccgttactagtggatccTCAGAATGAAAGCATCCTGTAAAAAT	

Supplementary Table S3. Plasmids used in this study.

Plasmids	Vector	Reference
Pro35S:PDLP5-HF	pB7-HFC	Lee et al., 2017
Pro35S:PDLP6-HF	pB7-HFC	Lee et al., 2017
Pro35S:SUS6-HF	pB7-HFC	This study
Pro35S:HF-YFP	pB7-HFN-Stop	Lee et al., 2017
Pro35S:PDLP5-YFP	pGW2-YFP	Reumann et al., 2009
Pro35S:PDLP6-YFP	pGW2-YFP	Reumann et al., 2009
Pro35S:SUS6-sfGFP	pEG-35S-GW-sfGFP	This study
ProPDLP5:PDLP5-YFP	pGWB540	Nakagawa et al., 2007
ProPDLP6:PDLP6-YFP	pGWB540	Nakagawa et al., 2007
ProPDLP6:PDLP6-YFP-3'UTR-Ter	ProPDLP6:PDLP6-YFP	This study
ProPDLP6:1xYFP	GW-YFP	This study
ProSWEET13:SWEET13-mCherry	pEG-GW-mCherry	This study
ProSUC2:PP2A1-mCherry	pEG-GW-mCherry	This study
ProSEOR2:SEOR2-mCherry	pEG-GW-mCherry	This study
ProPDLP5:GUS	pMDC163	Curti and Grossniklaus et al., 2003
ProPDLP6:GUS	pMDC163	Curti and Grossniklaus et al., 2003
pKIR-CRISPR-PDLP6	pKIR1.1	Tsutsui and Higashiyama, 2017
ProUBQ10:PDLP5-TbID-3xFlag	pEG-ProUBQ10-GW-TurboID-3xFLAG	This study
ProUBQ10:PDLP6-TbID-3xFlag	pEG-ProUBQ10-GW-TurboID-3xFLAG	This study
ProUBQ10:3xFlag-TbID-MCTP3	pEG-ProUBQ10-3xFLAG-TurboID-GW	This study
ProUBQ10:PDLP5-TbID-EYFP-3xFlag	ProUBQ10:PDLP5-TbID-3xFlag	This study
ProUBQ10:PDLP6-TbID-EYFP-3xFlag	ProUBQ10:PDLP6-TbID-3xFlag	This study
ProUBQ10:3xFlag-EYFP-TbID-MCTP3	ProUBQ10: 3xFlag-TbID-MCTP3	This study
pET-MBP-SUS6	pET17-HMR	Aung et al., 2020
pET-MBP-CalS1-CL	pET17-HMR	Aung et al., 2020
pET-MBP-CalS7-CL	pET17-HMR	Aung et al., 2020

References

Aung, K., Kim, P., Li, Z., Joe, A., Kvitko, B., Aflano, J.R., & He, S.Y. Pathogenic Bacteria Target Plant Plasmodesmata to Colonize and Invade Surrounding Tissues. *Plant Cell* 32, 595-611 (2020).

Lee, C.M., Adamchek, C., Feke, A., Nusinow, D.A. & Gendron, J.M. Mapping Protein-Protein Interactions Using Affinity Purification and Mass Spectrometry. *Methods Mol. Biol.* 1610, 231-249 (2017).

Nakagawa, T. et al. Improved Gateway binary vectors: high-performance vectors for creation of fusion constructs in transgenic analysis of plants. *Biosci. Biotechnol. Biochem.* 71(8), 2095-100 (2007).

Curtis, M.D & Grossniklaus, U. A gateway cloning vector set for high-throughput functional analysis of genes in planta. *Plant Physiol.* 133(2), 462-9 (2003).

Reumann, S. et al. In-depth proteome analysis of Arabidopsis leaf peroxisomes combined with in vivo subcellular targeting verification indicates novel metabolic and regulatory functions of peroxisomes. *Plant Physiol.* 150, 125–143 (2009).

Tsutsui, H. & Higashiyama, T. pKAMA-ITACHI Vectors for Highly Efficient CRISPR/Cas9-Mediated Gene Knockout in Arabidopsis thaliana. *Plant Cell Physiol.* 58(1), 46-56 (2017).
